# NHE Inhibition Does Not Improve Na^+^ or Ca^2+^ Overload During Reperfusion: Using Modeling to Illuminate the Mechanisms Underlying a Therapeutic Failure

**DOI:** 10.1371/journal.pcbi.1002241

**Published:** 2011-10-20

**Authors:** Byron N. Roberts, David J. Christini

**Affiliations:** Greenberg Division of Cardiology and Department of Physiology, Biophysics and Systems Biology, Weill Cornell Medical College, New York, New York, United States of America; University of California San Diego, United States of America

## Abstract

Reperfusion injury results from pathologies of cardiac myocyte physiology that develop when previously ischemic myocardium experiences a restoration of normal perfusion. Events in the development of reperfusion injury begin with the restoration of a proton gradient upon reperfusion, which then allows the sodium-proton exchanger (NHE) to increase flux, removing protons from the intracellular space while importing sodium. The resulting sodium overload drives increased reverse-mode sodium-calcium exchanger (NCX) activity, creating a secondary calcium overload that has pathologic consequences. One of the attempts to reduce reperfusion-related damage, NHE inhibition, has shown little clinical benefit, and only when NHE inhibitors are given prior to reperfusion. In an effort to further understand why NHE inhibitors have been largely unsuccessful, we employed a new mathematical cardiomyocyte model that we developed for the study of ischemia and reperfusion. Using this model, we simulated 20 minutes of ischemia and 10 minutes of reperfusion, while also simulating NHE inhibition by reducing NHE flux in our model by varying amounts and at different time points. In our simulations, when NHE inhibition is applied at the onset of reperfusion, increasing the degree of inhibition increases the peak sodium and calcium concentrations, as well as reducing intracellular pH recovery. When inhibition was instituted at earlier time points, some modest improvements were seen, largely due to reduced sodium concentrations prior to reperfusion. Analysis of all sodium flux pathways suggests that the sodium-potassium pump (NaK) plays the largest role in exacerbated sodium overload during reperfusion, and that reduced NaK flux is largely the result of impaired pH recovery. While NHE inhibition does indeed reduce sodium influx through that exchanger, the resulting prolongation of intracellular acidosis paradoxically increases sodium overload, largely mediated by impaired NaK function.

## Author Summary

Myocardial ischemia, commonly observed when arteries supplying the heart become occluded, results when cardiac tissue receives inadequate blood perfusion. In order to minimize the amount of cardiac damage, ischemic tissue must be reperfused. However, reperfusion can result in deleterious effects that leave the heart muscle sicker than if the ischemia had been allowed to continue. Examples of these reperfusion injuries include lethal arrhythmias and an increased region of cell death. Some of the early events that result in reperfusion injury include changes in pH and an overload of sodium inside the cell. During reperfusion, the sodium-proton exchanger (NHE) removes protons from the cell in an effort to restore normal pH, in turn importing sodium ions. Many strategies have been attempted to prevent reperfusion injury, including inhibition of the NHE, with little clinical effect. Using a mathematical model that we developed to study ischemia and reperfusion in cardiac cells, we found that NHE inhibition produces more severe sodium overload, largely due to adverse consequences of the delayed pH recovery produced by NHE inhibition. These results suggest that NHE inhibition alone may not be a viable strategy, and that therapies which prolong intracellular acidosis may be problematic.

## Introduction

Ischemia-reperfusion (IR) injury represents a constellation of pathological events that occur when previously ischemic myocardium experiences a restoration of normal tissue perfusion. IR injury, which can manifest as dangerous arrhythmias such as ventricular tachycardias and fibrillation, reduced myocardial force development, or an increased region of cell death, is likely to become even more clinically relevant in coming years owing to an aging population and the impact of aging on susceptibility to ischemia/reperfusion injury [1]. As such, it is desirable to develop an ability to effectively treat and prevent such phenomena.

**Figure 1:**
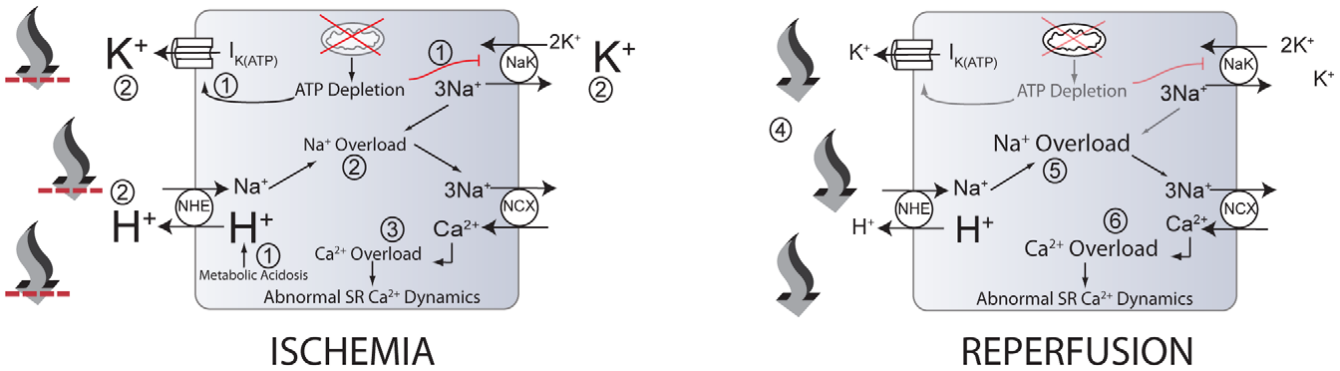
Some events that occur during myocardial ischemia and reperfusion. During ischemia, ATP depletion leads to inhibition of the sodium-potassium exchanger (NaK) and increased efflux through the ATP-regulated potassium channel (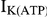) (1). Also, increased anaerobic metabolism produces a metabolic acidosis (1). Increased 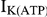 and decreased NaK flux contribute to the accumulation of extracellular potassium (2) (larger font). In addition, intracellular acidosis drives increased flux through the sodium-proton exchanger (NHE), contributing to extracellular acidosis (larger font) and intracellular sodium accumulation (2), worsened by decreased NaK flux. Increased intracellular sodium results in the sodium-calcium exchanger (NCX) operating more in the reverse mode, contributing to increased myoplasmic calcium concentration (3). High intracellular calcium concentrations can lead to abnormal sarcoplasmic reticulum calcium cycling and proarrhythmic phenomena. Upon reperfusion, washout of acidotic, hyperkalemic extracellular fluid occurs (4), reducing the concentrations of extracellular potassium and protons (smaller font). The resulting proton gradient allows increased flux through the NHE, resulting in exacerbations of intracellular sodium (5) and calcium (6) overloads (larger font) and additional proarrhythmic phenomena. Note that numbers in this legend correspond to encircled numbers in figure, not references.

Because of the danger that ischemia-reperfusion related events pose, there has been great interest in this problem for several decades. A large number of studies, directed at furthering the understanding of ischemia-reperfusion injury and examining many potential therapeutic targets, have been undertaken [2]–[4]. As a result of these studies, significant insight into the mechanisms of IR injury has been obtained. Figure 1 illustrates a chain of events that are believed to play a prominent role in ischemia-reperfusion injury [3]–[6]:

1. During ischemia, as the available oxygen is depleted, cells switch to anaerobic metabolism, with reduced ability to synthesize ATP. As anaerobic metabolism progresses, metabolic acidosis develops. This acidosis is exacerbated by the rise in the partial pressure of carbon dioxide in the ischemic region [7], [8]. The sodium-proton exchanger (NHE) attempts to correct intracellular pH (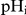) by exporting protons and importing sodium in exchange. Concurrently, decreasing concentrations of ATP lead to decreased flux through the sodium-potassium pump (NaK) and increased current through ATP-inactivated potassium efflux channels (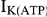).
2. Increased NHE flux and decreased NaK activity conspire to produce an overload of intracellular sodium (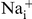). Also, decreased NaK flux and increased efflux through 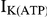 channels produce an elevated concentration of extracellular potassium (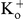), which carries proarrhythmic consequences such as shortening of the action potential duration and depolarization of the resting membrane potential (RMP). 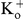 accumulation is also exacerbated by decreased extracellular volume (not shown).
3. The 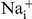 overload is partially relieved by the sodium-calcium exchanger (NCX) spending more time in reverse mode. However, increased reverse mode produces a secondary intracellular calcium (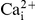) overload. 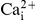 overload has been shown to be associated with pro-arrhythmic phenomena such as alternans [9] and spontaneous calcium releases [10]. Additionally, calcium overload can result in hypercontracture and cell death [4]. Complicating this scenario are the effects of acidosis and decreased [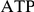] on multiple components of the calcium handling system. Decreased intracellular pH and/or [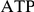] result in decreased flux through SERCA [8], [11]–[13], decreased ryanodine receptor release [8], [14], and decreased NCX flux [8], [11], [15].
4. Upon reperfusion, a washout of the acidic extracellular fluid occurs, restoring a proton gradient and allowing for increased NHE activity [2], [4].
5. Increased NHE activity results in a second spike in 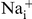, exacerbating the 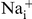 overload that developed during ischemia.
6. As during ischemia, this second 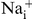 overload results in a second 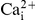 overload, especially as inhibition of the calcium-handling machinery is relaxed. In addition, [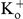] returns to normal [5] as tissue is reperfused, resulting in a return to normal resting membrane potential.

While much has been determined about the pathogenesis of reperfusion injury, we argue that a comprehensive understanding remains elusive. The most salient point is that despite numerous studies investigating many potential therapeutic agents to prevent reperfusion injury, efforts at translation of potential therapies to clinical practice have been largely unsuccessful [2], [3], [16].

One approach to treating ischemia-reperfusion injury has been inhibition of the NHE [2], [3], which is attractive for several reasons. The NHE is potentially responsible for a large amount of sodium permeability in cardiomyocytes [17]; the rationale is that if NHE flux is reduced during reperfusion, sodium overload can be mitigated and hence the secondary calcium overload can be reduced as well. In addition to reducing sodium and calcium-mediated reperfusion injury, NHE inhibition has been thought to play another beneficial role. It is believed that decreased ATP availability leads to activation of proteases and phospholipases that result in damage to the cell membrane, but that these enzymes have reduced activity under the relatively acidotic environment present during ischemia. Thus, when pH recovers during reperfusion, these enzymes increase in activity and cause damage. Therefore, NHE inhibition may reduce injury caused by proteases and phospholipases [17]. Despite some success in preclinical studies [4], NHE inhibition has had little beneficial effect in the clinical setting – when any beneficial effect is observed, it is when NHE inhibitors are administered prior to the onset of reperfusion [3], [4].

While there are several possible reasons for the failure to develop an effective treatment for reperfusion injury thus far [3], the most fundamental may be that cellular systems, not to mention myocardial tissue consisting of a large number of coupled cells, are highly coupled systems containing a large number of components, many exhibiting nonlinear relationships between them. Such systems are frequently robust to perturbations, often behave non-intuitively, and likely require observation and/or perturbation of more than just a couple of components in order to gain an adequate understanding of processes of interest.

Because of the aforementioned complexities, we aimed to develop a mathematical model that would enable us and others to gain insight into events that occur during myocardial ischemia and reperfusion, and to make predictions about possible therapeutic strategies. While models of ischemia already exist [6], [8], [18], none capture all of the relevant processes discussed above. In addition, these models were designed to address ischemia only, not reperfusion. Therefore, based upon experimental data from the literature, we have developed an improved mathematical model of the cardiomyocyte, and an accompanying protocol, that allows us to more realistically simulate myocardial ischemia and reperfusion. We then used this model to examine NHE inhibition as a therapeutic strategy. In our simulations, we observed that NHE inhibition produced no significant improvement in terms of sodium and calcium overload during reperfusion relative to control, and in some cases the amount of sodium and calcium overload was worse than control. In addition, we observed that NHE inhibition inhibited the recovery from intracellular acidosis and that the effects of prolonged acidosis on some subcellular components played a role in the failure to attenuate sodium overload.

## Methods

### Mathematical Model

**Figure 2:**
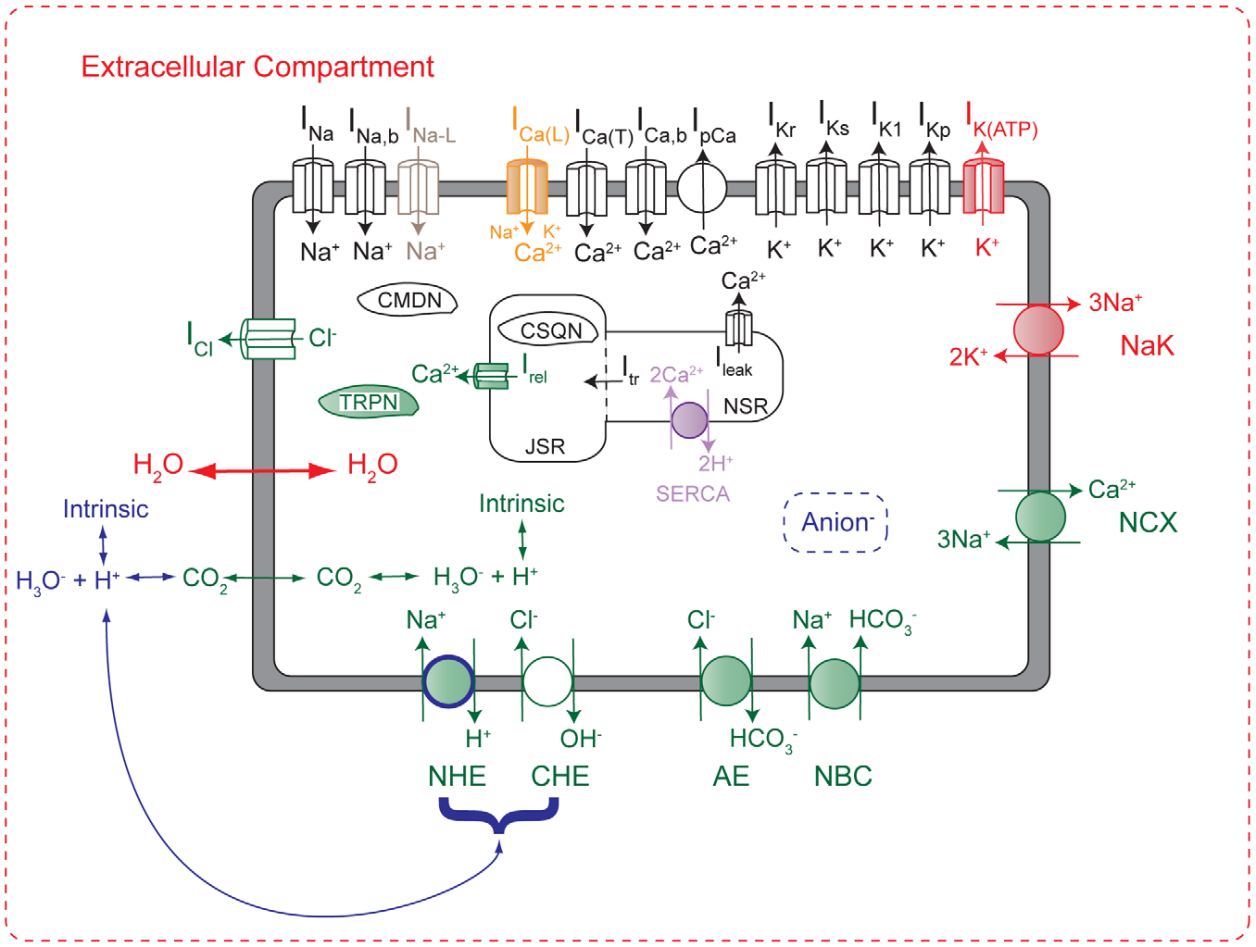
Mathematical model schematic. The model is an LRd model (black), with additions and modifications that were implemented from the CS (green) [8] and TCS [6] models (red), as well as an improved SERCA pump from [13] (purple), late sodium current from [27] (grey), the implementation of ATP-modified L-type calcium channel availability from [26] (orange), and novel additions by the authors (blue). Shaded components represent those that are regulated by pH and/or phosphometabolites. The model includes systems of equations that regulate concentrations of ATP, ADP, AMP, inorganic phosphate, creatine, and phosphocreatine, as well as impermeant metabolites that affect water flux. Abbreviations: Anion: generic anion species produced during ischemia; NCX, sodium-calcium exchanger; NHE, sodium-hydrogen exchanger; NBC, sodium-bicarbonate symporter; CHE, chloride-hydroxide exchanger; AE, anion exchanger; CMDN, calmodulin; TRPN, troponin C; CSQN, calsequestrin; JSR, junctional sarcoplasmic reticulum; NSR, network sarcoplasmic reticulum.

**Figure 3:**
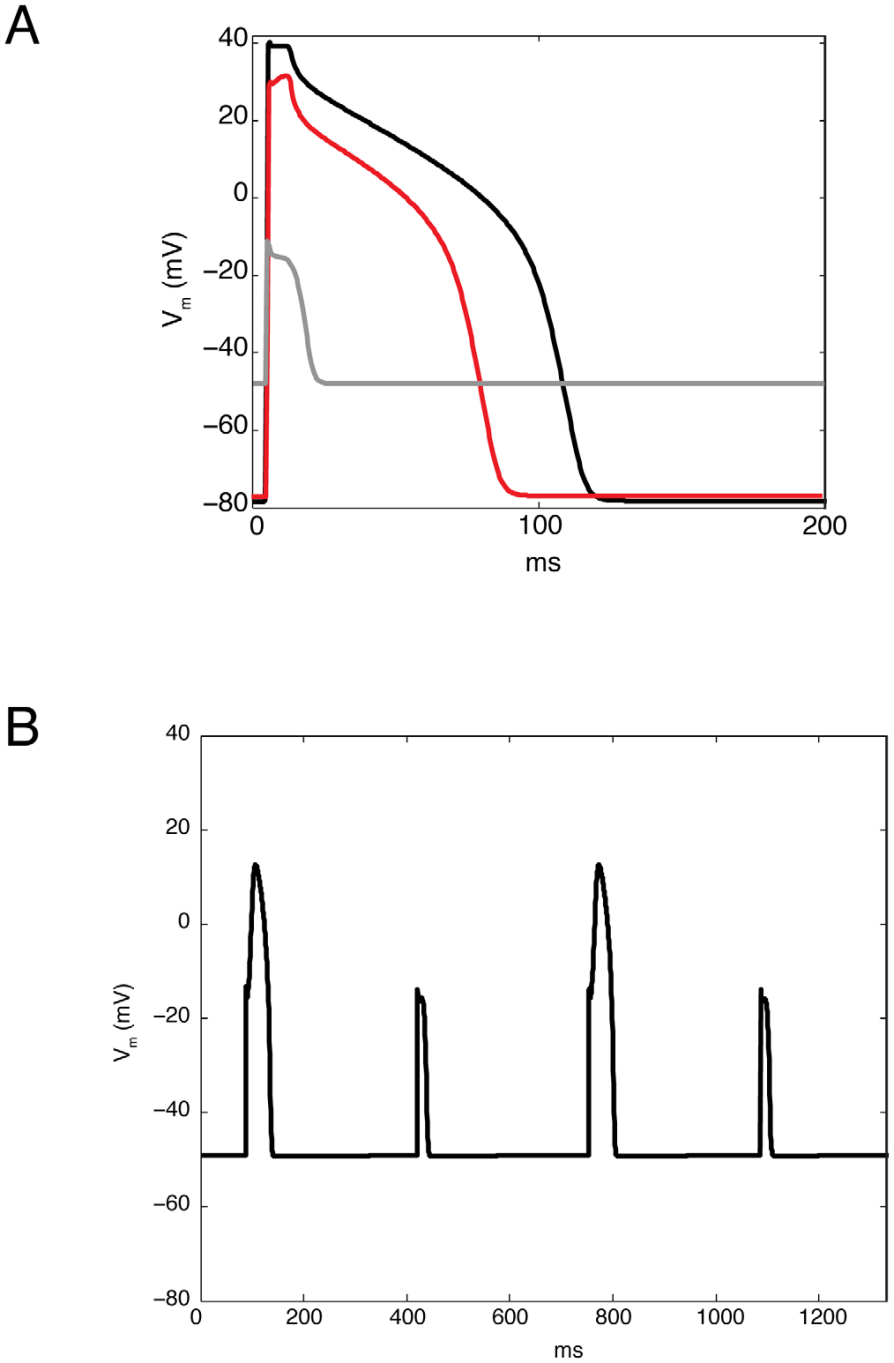
Representative action potentials from the control simulation (no NHE inhibition). In (A), the last action potential prior to the onset of ischemia (black), at the end of ischemia (gray), and after ten minutes of reperfusion (red) are shown. Panel (B) shows four responses to stimulation during late ischemia, approximately half a minute before the onset of reperfusion. The resting membrane potential is highly depolarized, and only every other stimulus generates an action potential.

This model (Figure 2) predominantly represents guinea pig physiology. Representative action potentials are shown in Figure 3. The starting point for this work was a model previously published by Crampin and Smith (CS model) [8], which is a charge-difference implementation of the Luo-Rudy dynamic (LRd) guinea pig ventricular myocyte model [19] where membrane voltage is calculated based upon changes in ion concentrations rather than the transmembrane currents [20], [21]. Modifications to the LRd designed to simulate the effects of respiratory acidosis (Figure 2 (black and green)) were introduced in the CS model, including the flux of species that are important for pH regulation across the plasma membrane, as well as dynamic intracellular pH that is subject to both bicarbonate and “intrinsic” buffering. However, extracellular pH changes in the CS model depend only on prescribed extracellular carbon dioxide (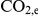) and static extracellular bicarbonate (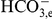) concentrations. In our model, both 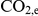 and 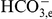 concentrations are dynamic during ischemia and reperfusion, as discussed below.

**Figure 4:**
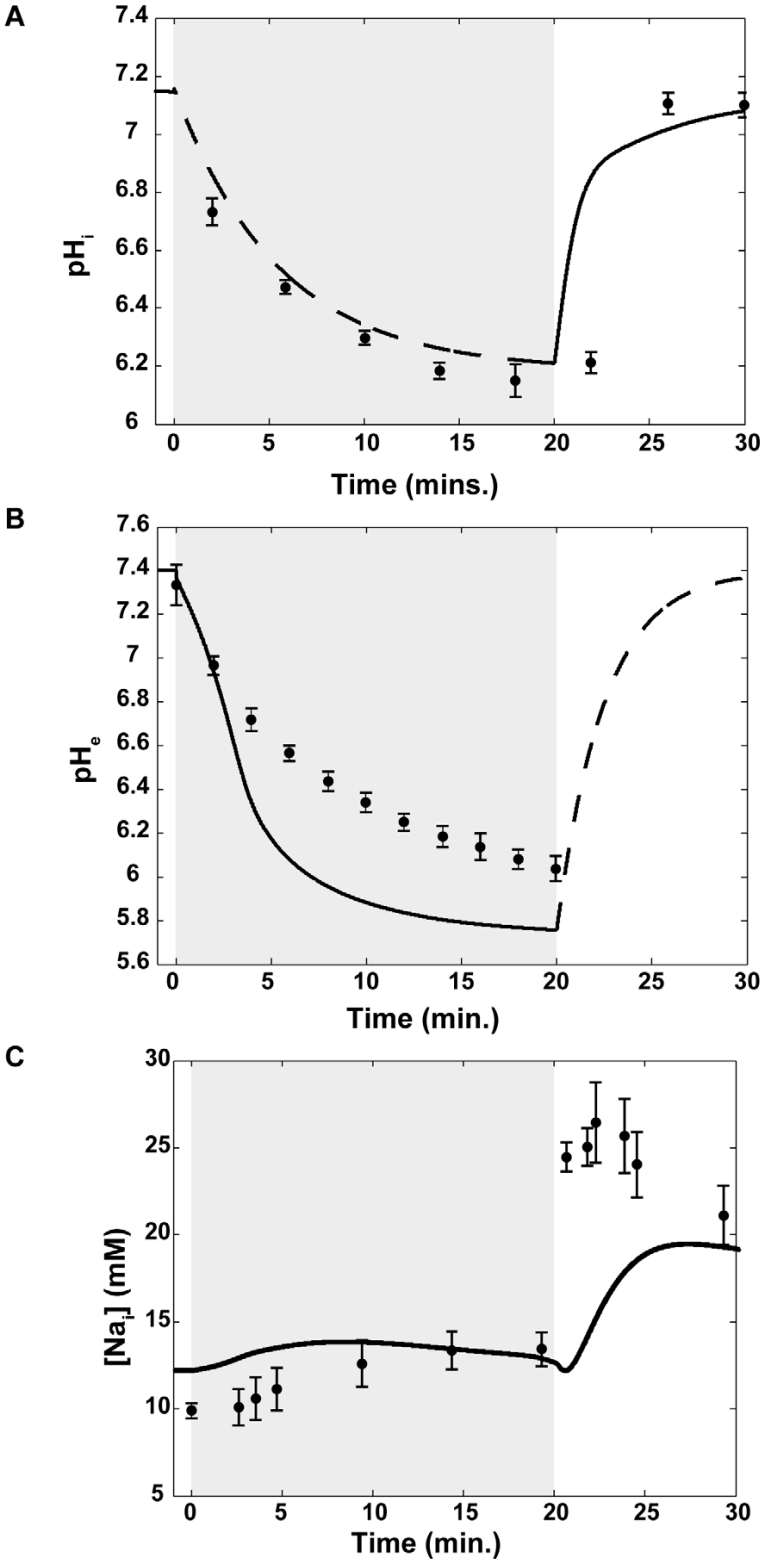
Comparison of mathematical model behavior to experimental data. Changes in (A) intracellular pH (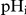), (B) extracellular pH (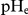), and (C) intracellular sodium (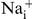) concentration during experimental (circles) and simulated (lines) ischemia (gray region) and reperfusion. For (A), the dashed line denotes the part of the simulation in which 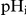 was prescribed by the ischemia-reperfusion protocol (Eq. 3). The solid line represents the part of the simulation in which 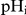 evolved according to the model equations (Eq. 29). Data points and error bars were extracted from [30] using the DigitizeIt software (share-it!, Denmark). For (B), the dashed line denotes the part of the simulation in which 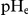 was prescribed by the ischemia-reperfusion protocol (Eq. 28). The solid line represents the part of the simulation in which 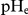 evolved according to the model equations (Eq. 4). Data points and error bars were extracted from [32] using the DigitizeIt software (share-it!, Denmark). For (C), data points and error bars were extracted from [33] using the DigitizeIt software (share-it!, Denmark).

The formulation of the NHE introduced in the CS model includes allosteric regulation whereby the exchanger is stimulated by the binding of intracellular protons. There is also the possibility [22] of additional allosteric regulation whereby the binding of extracellular protons inhibits the NHE, though whether this is truly the case is unclear. We have implemented this additional regulation in our model, as it improves model behavior in terms of extracellular acidosis that develops during ischemia. Total allosteric regulation of the NHE is calculated with the following equation:
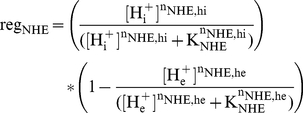
(1)The Hill coefficient for allosteric regulation by extracellular protons, 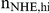, was reported to be about 1, which is the value that we use. Introducing extracellular allosteric increases the minimum extracellular pH during ischemia, resulting in a better fit to the data shown in Figure 4B, but also results in a worse fit to what is seen in terms of intracellular sodium concentration during ischemia. As depicted in Figure 4C, intracellular sodium accumulates during ischemia, and in late ischemia the rate of sodium accumulation may decrease. Introduction of extracellular allosteric NHE regulation creates a situation in which intracellular sodium concentration starts to decrease in late ischemia, approaching the pre-ischemia value. Increasing the value of 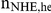 raises the minimum extracellular pH that is reached after 20 minutes of simulated ischemia, but also further decreases the intracellular sodium concentration. Therefore, we have chosen a value for 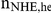 that is near what was reported experimentally, allows for intracellular sodium overload during ischemia, and results in a degree of extracellular acidosis that is relatively close to what has been observed experimentally. It should be noted that here we use the same value for the binding constant of protons, 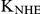, for both intracellular and extracellular regulation. The value used here is the same as that used in the CS model, and near what can be extracted for intracellular allosteric regulation from the data in [23]. When we extracted the binding constant for extracellular regulation from the same data and used this value for extracellular regulation, we saw a much worse fit in terms of the intracellular sodium profile.

As mentioned above, intracellular sodium accumulation falls off during late ischemia, and in a previous version of the model, sodium levels would approach or fall below pre-ischemic values. This is largely due to inhibition of the sodium-calcium exchanger, which is the primary mover of sodium into the cell. The sodium-potassium exchanger, which removes sodium, is also strongly inhibited during late ischemia, but there is still a net loss of sodium. In order to improve upon this, creating the sustained elevation of sodium above pre-ischemic levels throughout ischemia that is observed experimentally, we modified the pH dependence of the sodium-calcium exchanger. Specifically, the binding constant and Hill coefficient for proton binding to the sodium-calcium exchanger were changed to 7.0 and 0.75, respectively, from their published values of 7.37 and 0.99 [8].

The model, as originally implemented, exhibited high concentrations of intracellular sodium at steady state under pre-ischemic conditions. In addition, the relationship between steady state sodium concentration and pacing frequency was steeper than both experimental measurements and simulated experiments using the LRd model. In order to bring sodium homeostasis in our model in line with the LRd and closer to what has been seen experimentally, we decreased the scaling factor for the sodium-calcium exchanger, c1, by 40 percent from the value published in [8]. The current sodium-pacing frequency relationship is provided in Figure S4 in Text S1. Decreasing baseline sodium-calcium exchange flux reduces steady state sodium load, but also increases intracellular calcium. In order to compensate for this, it was necessary to increase maximum calcium pump current, 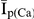, from 1.15 [8] to 1.65 uA/uF.

In order to simulate the effects and proximal causes of extracellular potassium (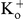) accumulation during ischemia, we implemented components that were introduced in a model that extended the LRd model with the addition and/or modification of components to study extracellular potassium accumulation during ischemia [6]. We will henceforth refer to this model as the Terkildsen-Crampin-Smith (TCS) model. Note that the TCS model is not an extension of the CS model, as it does not include a pH regulation system (i.e. equilibration with carbon dioxide, bicarbonate and intrinsic buffering), acid transporters, or the inhibitory effects of acidosis on various components of the calcium handling system. Among other additions, our new model incorporates all CS and TCS components, save for those relating to simulating the development of tension in the CS model. The TCS additions (Figure 2 (red)) allow the sodium-potassium exchange pump to behave in a more realistic fashion, as it is sensitive to changes in the concentration of sodium and potassium on both sides of the plasma membrane, as well as phosphometabolites and 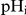. The TCS model also incorporates 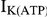 and dynamic intra- and extracellular volumes coupled to water flux. These potassium-relevant processes have been incorporated into our model, with changes.

First, we have modified the function for calculating the amount of water flux to include concentrations of intracellular and extracellular chloride ([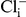] and [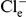], respectively):
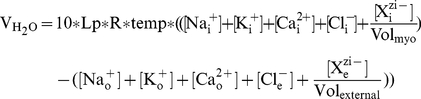
(2)where Lp is the hydraulic conductivity of the membrane, R is the universal gas constant, temp is the temperature, [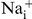], [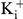], [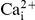], [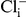] are the intracellular concentrations of sodium, potassium, calcium and chloride, respectively, [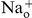], [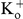], [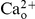], [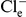] are the extracellular concentrations of sodium, potassium, calcium and chloride, respectively, [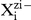] and [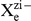] are the concentrations of non-permeable osmolytes in the intra- and extracellular compartments, respectively, and 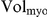 and 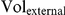 are the volumes of the myoplasm and extracellular compartment, respectively. [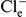] is used when initializing [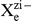], and [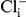] is used when initializing and updating [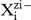]. Second, the equation that prescribes 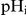 during ischemia has been slightly modified to match a slightly different value for 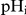 at the transition from simulated pre-ischemia to ischemia. During ischemia:
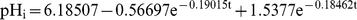
(3)where t is the ischemic time in minutes. It should be noted that the first term of the 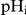 ischemia equation may need to be modified if the model is used with different initial conditions.

As the dynamic nature of extracellular acid status is believed to play a prominent role in reperfusion arrhythmogenesis, it is highly desirable to employ a mathematical model that includes a dynamic extracellular pH system that is as realistic as possible. To this end, we have made several significant improvements to the representation of extracellular pH over preexisting models (Figure 2 (blue)). First, we have introduced intrinsic buffering in the extracellular compartment. Second, as mentioned previously, [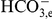] is dynamic during ischemia and reperfusion. Third, flux through NHE and CHE affects not only 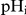, but also 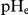. Thus, 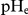 during simulated ischemia is influenced by a combination of equilibration with dynamic 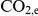 and 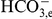 concentrations, as well as an intrinsic buffering system, and flux of protons and hydroxide ions across the plasma membrane. The change in extracellular pH at each time step is:
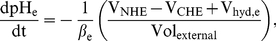
(4)where
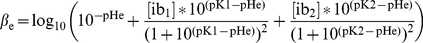
(5)is the intrinsic buffering power in the extracellular compartment and
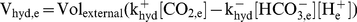
(6)represents equilibration between protons, bicarbonate and carbon dioxide in the extracellular compartment. Eqs. 4–6 are nearly the same as their intracellular counterparts, as provided in [8] save for the reversal of signs for 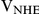 and 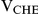 and the substitution of extracellular for intracellular species concentrations and volumes. Concentrations and pK values of the intrinsic buffers are the same as in the intracellular compartment. Equations for 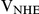 and 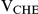 are provided in Text S1 and [8].

Because the concentrations of protons are explicitly represented in our model, and because part of the ischemia simulation protocol is to impose a progressive intracellular acidosis on the system during ischemia, it is necessary to account for conjugate anions that are produced as the metabolic acidosis progresses in order to maintain charge conservation. This is accomplished by the introduction of a variable representing the concentration of generic monovalent anions produced in a 1∶1 stoichiometry with the surplus protons that appear as 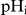 decreases.
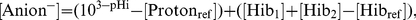
(7)where
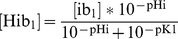
(8)and
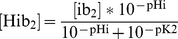
(9)are the concentrations of protons bound to the two generic intrinsic buffers, 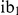 and 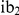, and
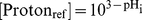
(10)and
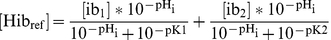
(11)are the concentrations of free intracellular protons and protons bound to the intrinsic buffers, respectively, at the beginning of a simulation, such that [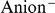] is initially equal to zero. Eqs. 8 and 9 are from [8].

Experiments have shown that the availability of L-type calcium channels (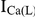) is modified by the concentration of ATP [24], [25]. Accordingly, we have included a term to represent this change in channel availability (Figure 2 (orange)) [26]:
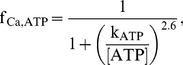
(12)where [ATP] is the concentration of ATP and 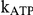 is the 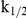 for the binding of ATP to 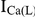 channels.

Finally, we implemented a newer version, relative to that used in the CS model, of the SERCA pump (Figure 2 (purple)) from [13]. The cycling rate of SERCA, 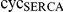, (see Text S1 or [13]) is translated to calcium flux via the following equation:
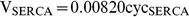
(13)The conversion factor of 0.00820 was derived by first implementing SERCA such that it was unidirectionally coupled (i.e. could respond to changes in 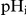, [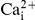], [ATP], etc., but could not affect the remainder of the system) and simulating normal pre-ischemic conditions. Flux through the CS model SERCA and 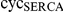 were recorded at the peak of the intracellular calcium transient at pre-ischemic 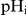. These values were used in our model to scale the raw 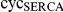 value to produce 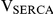 output that results in appropriate 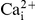 transients.

### Ischemia and Reperfusion Simulation Protocols

Throughout all simulations, the cell was constantly stimulated at a rate of 3 Hz using pulses of −80.0 uA/uF and lasting 0.5 ms. Starting from the initial conditions provided in Table 1, the cell was paced for five minutes of simulated time, well past the required time for the model to reach steady state, before the onset of simulated ischemia. During pre-ischemia, the concentrations of all extracellular species are held constant, the assumption being that the extracellular compartment has access to, and is in equilibrium with, a pool that is many orders of magnitude larger than the volume of the immediate extracellular space. 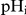, on the other hand, is dynamic and is updated per Eq. 29 below, although it fluctuates very little under these conditions.

**Table 1:** Initial conditions.

Parameter	Value
V	−77.00
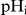	7.15
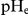	7.40
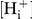	0.000070795
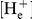	0.000039811
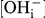	0.000141254
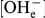	0.000251189
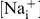	14.34
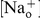	149.02
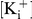	109.10
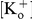	5.40
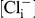	33.77
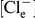	146.7
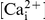	0.000335
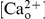	1.80
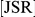	0.605236
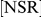	2.352399
m	0.005751
h	0.894526
j	0.889565
d	0.000022
f	0.990176
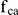	0.640341515
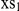	0.059092
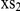	0.108548
xr	0.005171
b	0.002925
g	0.899522
p	0.018649
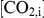	0.741684
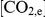	0.741684
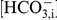	7.90
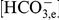	11.90
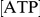	7.216
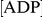	0.036
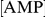	0.000184
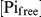	0.8
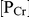	13.3
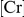	8.9
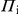	310.0
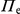	310.0
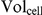	0.00003801
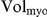	0.000025847
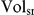	0.000002281
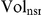	0.000002098
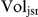	0.000000182
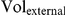	0.000005172
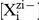	0.00393598
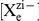	0.000036618

In this study we have simulated an abrupt-onset total ischemia. During ischemia, as oxygen availability falls and the cell switches to anaerobic metabolism, metabolic acidosis develops. The metabolic byproducts responsible for this acidosis are not represented in our model, and so we impose metabolic acidosis on the intracellular compartment via Eq. 3. Also, during ischemia the extracellular compartment becomes more acidic due to flux through the acid exchangers and the accumulation of carbon dioxide. In our model the extracellular compartment is considered to be completely isolated, such that gases and ions accumulate or deplete via flux through ion channels and transporters. While we must impose pH changes on the intracellular compartment during simulated ischemia, pH changes in the extracellular compartment are allowed to respond to acid exchanger flux and carbon dioxide accumulation, and are calculated per Eq. 4. Accumulation of carbon dioxide in the extracellular space is modeled by:
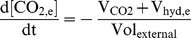
(14)where 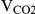 is transport of 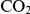 across the plasma membrane and 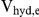 represents the concentration of 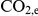 produced or consumed from equilibration with 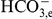 in the extracellular compartment (Eq. S.167 in Text S1).

[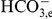], [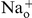], [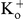], and [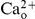], which are no longer fixed as these species are accumulating or being depleted from the extracellular compartment while it is isolated during simulated ischemia, are updated per the following equations:
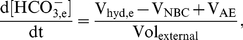
(15)

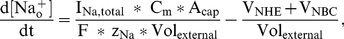
(16)

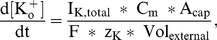
(17)and
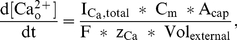
(18)where 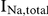, 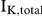, and 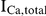 represent the total current through relevant ion channels, the NaK exchanger and NCX exchanger for 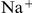, 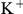, and 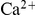, respectively. Eqs. 15–18 are derived from the equations for updating intracellular species concentrations in [8].

During ischemia, sodium channel conductance, 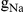, is decreased from 16.0 to 14.4 mS/uF. In addition, late sodium current, defined by [27]:
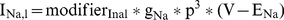
(19)is increased by increasing the value of 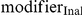 from 0.0007 to 0.00018.

Ischemia results in decreased availability of ATP and phosphocreatine. We impose decreasing concentrations of ATP and PCr on the system during simulated ischemia, which then affect concentrations of the other phosphometabolites, per the following equations, from [6]:
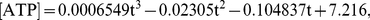
(20)

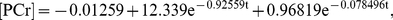
(21)

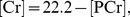
(22)

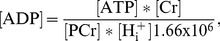
(23)

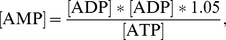
(24)and
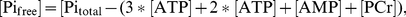
(25)where t is the ischemic time in minutes and [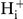] = 1000*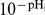. Note that Eq. 20 has been modified from [6].

Finally, as described in [6], a variable representing intracellular osmolarity is linearly increased during ischemia:
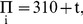
(26)where t is the ischemic time in minutes. 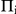 is subsequently used to update [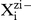] at each time step:
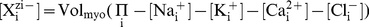
(27)for use in calculating water flux (see Text S1).

As discussed above, it is believed that acidic extracellular fluid is washed out during reperfusion and replaced with fluid from elsewhere that is at normal pH, carrying away accumulated protons and carbon dioxide. Also, oxygen concentration increases, reducing the cell's dependence on anaerobic metabolism and abolishing the source of metabolic acidosis. Therefore, as opposed to simulated ischemia, during which 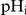 is prescribed and 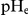 evolves according to Eq. 4, during simulated reperfusion 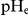 is prescribed according to Eq. 28 in order to simulate washout and 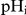 is allowed to evolve according to Eq. 29 below. 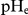 is prescribed according to the following formula:
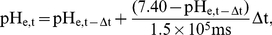
(28)where 7.40 is the pre-ischemic 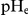 and 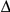
t is the length of the time step. When using a time step of 0.005 ms, the time constant of 1.5×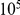 ms produces a recovery to pre-ischemic 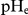 in about 10 minutes. We have attempted to choose a recovery rate that is consistent with what has been observed experimentally, but data regarding the 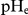 recovery profile during reperfusion is scarce. In one porcine experiment [28], pre-ischemic 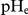 was reached after about 10 minutes, following 10 minutes of ischemia. In another experiment performed on canines, recovery took 20–30 minutes following 90 minutes of ischemia [29]. We have chosen a recovery time of approximately 10 minutes because it produces a better fit to experimental data for other parameters, such as 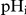 and [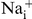].

The change in 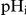 at each time step is calculated according to the following formula:
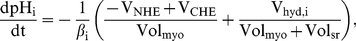
(29)where
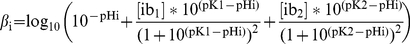
(30)is the intrinsic buffering power in the intracellular compartment, 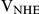 and 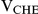 represent flux through the NHE and CHE, respectively, 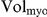 and 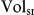 represent the volumes of the myoplasm and sarcoplasmic reticulum, respectively, and
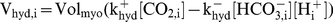
(31)represents equilibration between protons, bicarbonate and carbon dioxide in the intracellular compartment. Eqs. 29–31 are from [8]. Equations for 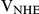 and 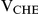 are provided in Text S1 and [8].

It is assumed that other extracellular species (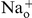, 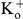, 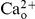, 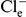, 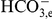, and 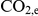) will return to their normal concentrations during reperfusion, now that they are again in equilibrium with a vastly larger pool. We have chosen a time constant of 3.75×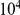 ms, such that the concentration of a particular ion or molecule is updated according to the following equation:
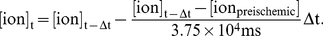
(32)This produces a recovery to preischemic concentrations in approximately two minutes, consistent with what has been observed during reperfusion in Langendorff-perfused tissue [5]. The idea here is to simulate the wash out (or wash in, as the case may be) of extracellular species as the fluid in the previously isolated extracellular compartment is replaced with fluid replenished by blood arriving from outside the ischemic region.

Based upon experimental evidence, it appears that ATP and PCr can recover to as much as 40 and 75 percent of their preischemic concentrations following ischemia in Langendorff-perfused guinea pig hearts [30]. It also appears that most recovery happens within the first few minutes of reperfusion. Therefore, [ATP] and [PCr] are calculated according to the following equations during simulated ischemia:
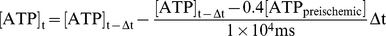
(33)

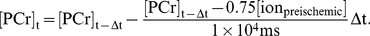
(34)The concentrations of all other phosphometabolites are calculated as they were during ischemia.

Finally, intracellular osmolarity is returned to normal according to the following equation:
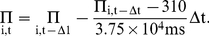
(35)


### NHE Inhibition

Flux through the NHE is computed using the following equation:
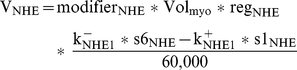
(36)(see Text S1 for preceding equations). In order to simulate the effects of agents that inhibit the NHE, we reduced the value of 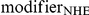 from its default value of 1.0 to one of two values: 0.50 or 0.0. In addition, we introduced each of these degrees of NHE inhibition at one of three time points: the end of 20 minutes of ischemia (denoted reperfusion in figures), after 10 minutes of ischemia (denoted mid-ischemia in figures), or at the onset of ischemia (denoted ischemia in figures).

Differential equations were solved using the forward Euler method. Model code is available as a supplemental download.

## Results

### Membrane Voltage

Figure 3A illustrates action potentials before (black) and at the end (gray) of ischemia, as well as following 10 minutes of reperfusion (red), from the control simulation. Note that after ten minutes of reperfusion, the action potential duration and amplitude are still smaller than before the onset of ischemia, consistent with what has been observed in guinea pig experiments [31]. Figure 3B illustrates responses to four stimulus events during late ischemia. Note that the resting membrane potential is depolarized to a degree similar to that which has been observed in guinea pig experiments [5]. Also, cell excitability is extremely impaired, with only every other stimulus eliciting an action potential.

### pH and Ion Concentrations

Figure 4 provides a comparison of experimental data to results obtained when we simulated 20 minutes of ischemia (grey shading) followed by 10 minutes of reperfusion. The simulation results in all panels of Figure 4 (solid lines) are from the control simulation, in which no NHE inhibition was instituted. Comparing simulation results to experimental data from [30] in Figure 4A, it can be seen that our model closely reproduces the behavior of intracellular pH observed in guinea pig myocytes during ischemia and reperfusion. As discussed above, 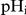 during simulated ischemia evolves according to a fixed equation (Eq. 3) implemented to reproduce what occurs experimentally. However, 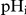 during reperfusion, which is calculated by Eq. 29, is not pre-determined.

In Figure 4B, results from the same control simulation are compared to experimentally measured 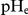 during 20 minutes of guinea pig ischemia [32]. Our model and simulation protocol produce a 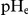 profile that closely reproduces what is observed during early ischemia, but continues with a steep decline for a longer period of time, producing an extracellular pH after 20 minutes of ischemia that is about 0.2 units lower than what was seen in the guinea pig heart.

Figure 4C compares results from the same control simulation to [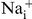] measured in guinea pig hearts during ischemia and reperfusion [33]. In both cases, there is an accumulation of sodium inside the cell during ischemia, although our model shows a more modest increase. This is largely due to the higher starting concentration, which is determined from a steady state value that is heavily dependent upon NaK pump density and the amount of allosteric regulation by extracellular protons at the NHE. Also, both the experimental and simulated results show a second, more profound, sodium overload upon reperfusion. It should be noted that in the experiment cited here, reperfusion was preceded by 30 minutes of ischemia, not 20, as is the case with the other experimental comparisons.

**Figure 5:**
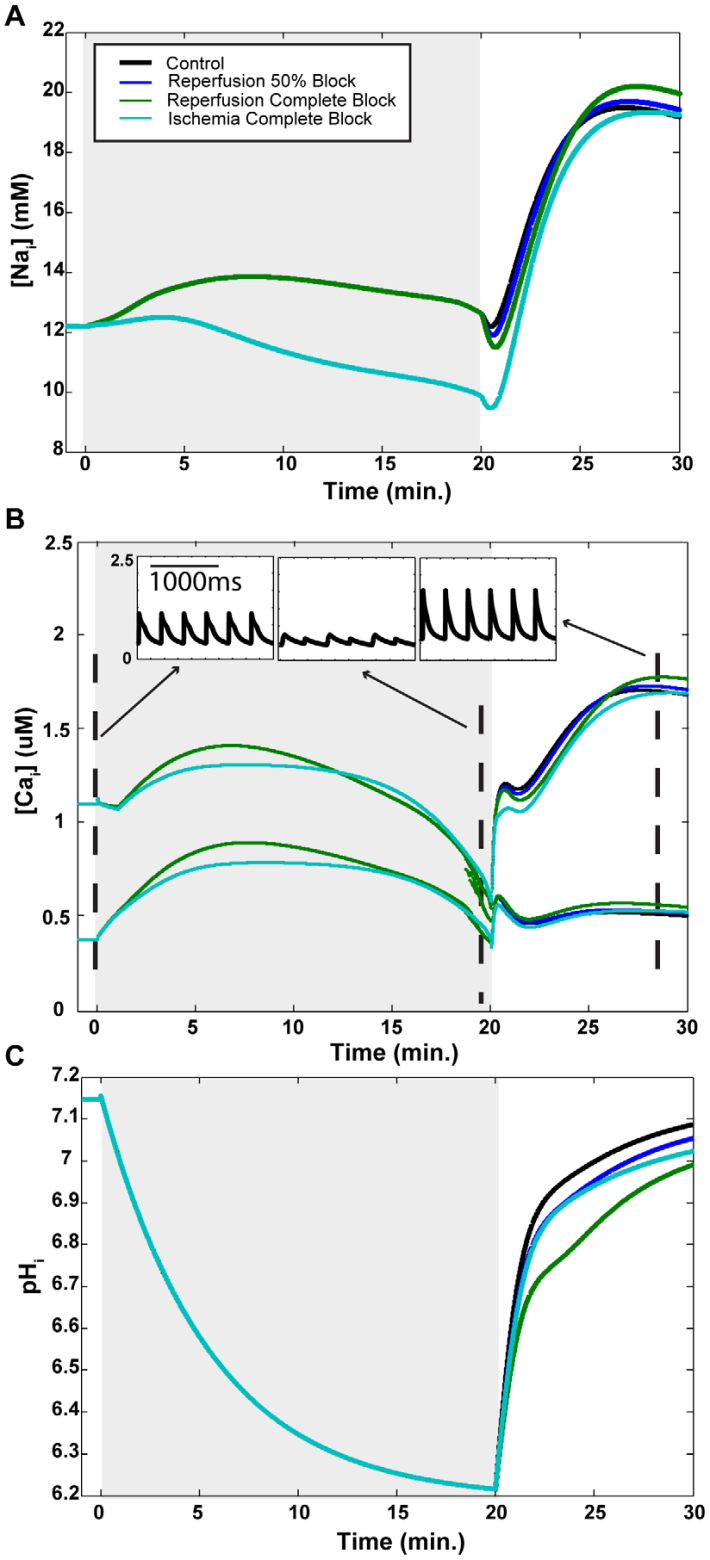
Changes in ion concentrations and pH during simulated reperfusion. (A) intracellular sodium (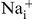), (B) maximum and minimum intracellular calcium (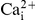), and (C) intracellular pH (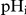) during simulated ischemia (gray region) and reperfusion during four simulations: Control (with no NHE inhibition) (black), NHE inhibition beginning with reperfusion at 50 percent (blue) or 100 percent (green) reduction, and 100 percent NHE blockade beginning with ischemia (light blue). In (B), the three insets, plotted on the same vertical axis as the main figure, show 2000 ms of intracellular calcium transients corresponding to the regions denoted by the vertical dashed lines: just prior to the onset of ischemia (left); during late ischemia, demonstrating alternans (middle); and late in the observed reperfusion window (right).

**Figure 6:**
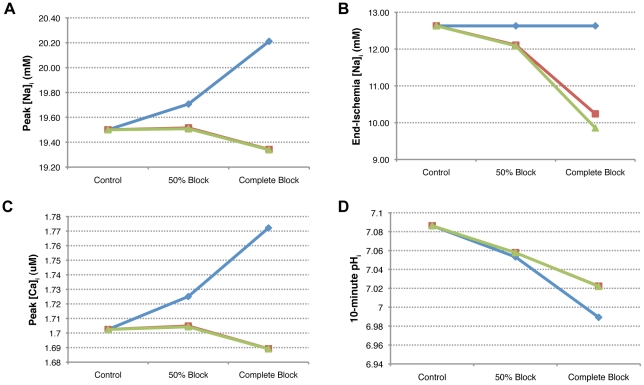
Changes in ion concentrations and pH: peak intracellular sodium during reperfusion (A), end-ischemic intracellular sodium (B), peak intracellular calcium during reperfusion (C), and intracellular pH after 10 minutes of reperfusion (D). In addition to the control simulation (no NHE inhibition), simulations were performed in which NHE flux was either reduced by 50 percent or completely blocked beginning at the onset of reperfusion (blue), half-way through ischemia (red), or at the onset of ischemia (green).

Figure 5 shows intracellular sodium, calcium, and pH profiles from a subset of simulations. The black lines representing the control simulations in this and subsequent figures are the same as shown in Figure 4. In addition to the control simulation, results from the following three simulations are shown: either a 50 or 100 percent reduction in NHE flux beginning at the onset of reperfusion, and a 100 percent reduction at the onset of ischemia (denoted as R50, R0, and I0 in tables). In Figure 5A, the intracellular sodium concentration throughout each of the four aforementioned simulations can be seen. Table 2 provides peak and mean intracellular sodium concentrations during reperfusion, the concentration of sodium at the end of ischemia, and ratios of peak to end-ischemic sodium concentrations for all simulations. Peak and end-ischemia sodium concentrations for all simulations are shown in Figures 6A and 6B, respectively. One obvious feature of Figures 5A and 6A is that there is minimal, if any, benefit in terms of peak sodium concentration during reperfusion, regardless of when and how much NHE inhibition is imposed. In fact, when NHE inhibition is instituted at reperfusion, there is a worsening of peak sodium concentration during reperfusion relative to control (green and blue lines). In simulations where NHE inhibition was started at earlier time points, there is a reduction in [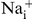] prior to the onset of reperfusion. However, this appears to have a relatively small impact on the concentrations of sodium at the end of the observed reperfusion period.

**Table 2:** Intracellular sodium.

	Control	R50	R0	M50	M0	I50	I0
Peak [ 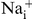 ]	19.50	19.71	20.21	19.52	19.34	19.51	19.34
Mean [ 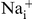 ]	17.48	17.47	17.46	17.24	16.58	17.23	16.47
End-ischemic [ 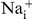 ]	12.63	12.63	12.63	12.11	10.24	12.09	9.85
Peak/End [ 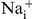 ] Ratio	1.54	1.56	1.60	1.61	1.89	1.61	1.96
Normalized Ratio	1.00	1.01	1.04	1.04	1.22	1.05	1.27

Concentrations in mM.

Figure 5B plots the maximum and minimum myoplasmic calcium concentrations for each beat in the same four simulations. The three insets show calcium transients, plotted on the same vertical axis as panel B, for 2000 ms in the regions marked by dashed vertical lines. Table 3 provides peak and mean concentrations during reperfusion and at the end of ischemia, as well as ratios, as in Table 2. Peak 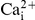 concentrations for all simulations are graphed in Figure 6C. The largest calcium concentrations were seen during the R0 (complete NHE block starting at reperfusion) simulation, as was also the case for sodium (Figure 6A and Table 2).

**Table 3:** Intracellular calcium.

	Control	R50	R0	M50	M0	I50	I0
Peak [ 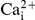 ]	0.001702	0.001725	0.001772	0.001705	0.001689	0.001704	0.001689
Mean [ 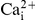 ]	0.000738	0.000751	0.000780	0.000738	0.000729	0.000739	0.000730
End-ischemic [ 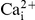 ]	0.000400	0.000400	0.000400	0.000394	0.000412	0.000471	0.000451
Peak/End [ 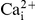 ] Ratio	4.26	4.31	4.43	4.33	4.10	3.62	3.75
Normalized Ratio	1.00	1.01	1.04	1.02	0.96	0.85	0.88

Concentrations in mM.

Intracellular pH profiles for the same four simulations are shown in Figure 5C. The values of intracellular pH after 10 minutes of reperfusion (graphed in Figure 6D), as well as the mean 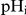 during reperfusion, are provided in Table 4. Relative to the control simulation, 10-minute and mean 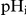 values are lower regardless of when and how much NHE inhibition was imposed. The lowest pH values were observed during the R0 simulation, which is also the simulation in which the most severe sodium and calcium overloads were observed.

**Table 4:** Intracellular pH.

	Control	R50	R0	M50	M0	I50	I0
End 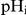	7.09	7.05	6.99	7.06	7.02	7.06	7.02
Mean 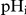	6.93	6.89	6.80	6.90	6.88	6.90	6.87

### Sodium Flux

**Figure 7:**
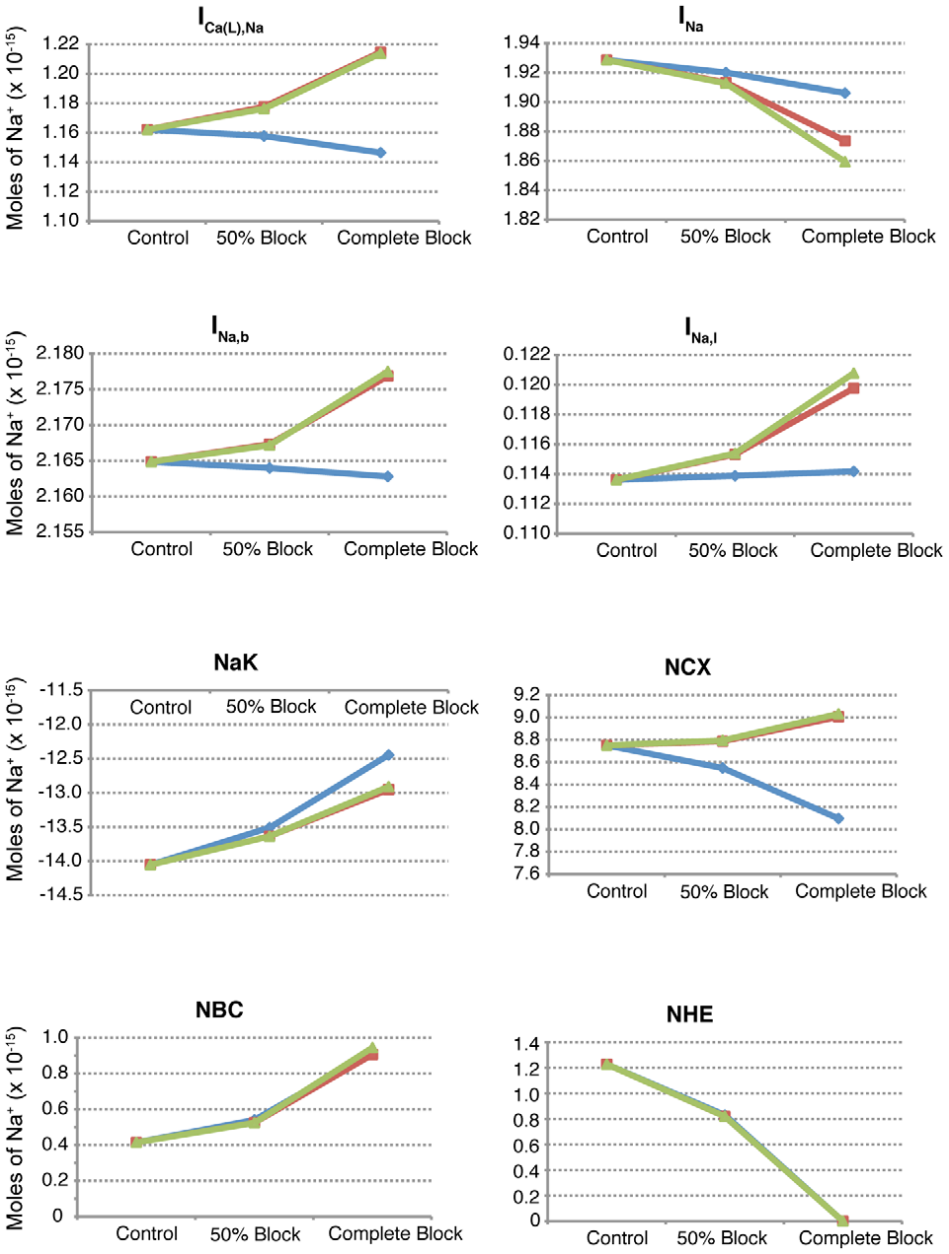
Number of sodium ions moved during each simulated reperfusion by each of eight sodium components: the L-type calcium channel ( 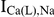
 **), rapid sodium current (** 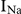 **), background sodium current (** 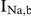 **), late sodium current (** 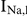 **), sodium-potassium exchanger (NaK), sodium-calcium exchanger (NCX), sodium-bicarbonate symporter (NBC), and sodium-exchanger (NHE).** In addition to the control simulation (no NHE inhibition), simulations were performed in which NHE flux was either reduced by 50 percent or completely blocked beginning at the onset of reperfusion (blue), half-way through ischemia (red), or at the onset of ischemia (green).

In our model, there are eight components through which sodium can enter and/or leave the cell: the sodium-hydrogen exchanger (NHE) and sodium-bicarbonate symporter (NBC), two of the so-called acid exchangers, the sodium-calcium exchanger (NCX), sodium-potassium exchanger (NaK), fast-sodium current (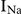), background sodium current (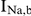), late sodium current (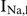), and sodium movement through the L-type calcium channel (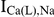). In order to develop an understanding of the relative importance of each of these eight sodium flux pathways to sodium overload, we analyzed current through each of these during the ten minutes of simulated reperfusion. Table 5 and Figure 7 provide the net number of moles of sodium moved through each component during the ten minutes of reperfusion. This value was calculated as per Eq. 37 for NHE and NBC and Eq. 38 for the remaining six ion channels and exchangers:
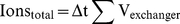
(37)

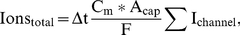
(38)where 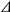
t is the time step, 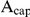 is the capacitive area, and F is Faraday's constant, as provided in Text S1. 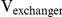 is the flux through either the NHE or NBC at a given time step, and 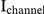 is the current through one of the six ion channels or exchangers at a given time step. For the NCX and NaK, the value calculated by Eq. 38 is multiplied by 3. Note that this is not the true number of moles of ions moved. Mathematical simulations are discretized into finite time steps, and data was not written at every time step in order to keep data file sizes within manageable limits. Nonetheless, these values provide a standard with which the contributions of each of the eight sodium components can be compared across different simulations. In these simulations, data was sampled every tenth time step. The only sodium pathway which has a net negative inward sodium movement is the NaK. The NCX, which functions in both forward (sodium in) and reverse (sodium out) modes, increasingly favors the reverse mode during sodium overload,

**Table 5:** Net number of sodium moles moved (×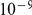).

	Control	R50	R0	M50	M0	I50	I0
NHE	1.23E−06	8.30E−07	0.00E+00	8.19E−07	0.00E+00	8.20E−07	0.00E+00
NBC	4.14E−07	5.40E−07	9.19E−07	5.25E−07	9.06E−07	5.24E−07	9.45E−07
NCX	8.75E−06	8.55E−06	8.10E−06	8.79E−06	9.00E−06	8.80E−06	9.03 E−06
NaK	−1.40E−05	−1.35E−05	−1.24E−05	−1.36E−05	−1.30E−05	−1.36E−05	−1.29E−05
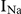	1.93E−06	1.92E−06	1.91E−06	1.91E−06	1.87E−06	1.91E−06	1.86E−06
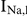	1.14E−07	1.14E−07	1.14E−07	1.15E−07	1.20E−07	1.15E−07	1.21E−07
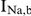	2.16E−06	2.16E−06	2.16E−06	2.17E−06	2.18E−06	2.17E−06	2.18E−06
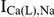	1.16E−06	1.16E−06	1.15E−06	1.18E−06	1.22E−06	1.18E−06	1.21E−06
Total	2.98E−05	2.88E−05	2.68E−05	2.91E−05	2.82E−05	2.91E−05	2.83E−05

We also calculated the proportion of total moles of sodium moved for each of the eight sodium pathways, presented in Table 6. The proportion values were calculated by taking the absolute value of the total ion movement for a given component and simulation, and dividing by the sum of the absolute values for all eight components in the same simulation. As can be seen in Table 6, the NaK and NCX are responsible for most of the sodium movement into and out of the cell.

**Table 6:** Proportion of total number of sodium moles moved.

	Control	R50	R0	M50	M0	I50	I0
NHE	0.041	0.029	0.000	0.028	0.000	0.028	0.000
NBC	0.014	0.019	0.034	0.018	0.032	0.018	0.033
NCX	0.293	0.297	0.302	0.302	0.319	0.302	0.320
NaK	0.471	0.469	0.465	0.468	0.458	0.468	0.457
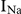	0.065	0.067	0.071	0.066	0.066	0.066	0.066
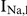	0.004	0.004	0.004	0.004	0.004	0.004	0.004
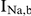	0.073	0.075	0.081	0.074	0.077	0.074	0.077
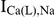	0.039	0.040	0.043	0.040	0.043	0.040	0.043

As additional model validation, we performed additional simulations and compared the results to an analysis of sodium fluxes in rabbit myocytes [34]. The model was allowed to reach steady state either when paced at a rate of 1 Hz under pre-ischemic conditions, left at rest under pre-ischemic conditions, or left at rest in mild acidosis (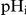 set to 6.9). Sodium flux through all seven components that produce net inward sodium movement were analyzed for the last minute of simulated time (please see Figure S1 in Text S1 for results). As expected, and consistent with data summarized in [34], there was less sodium influx when the cell was at rest than when paced, due to reduced current through the sodium channels and NCX. However, also as expected and consistent with experimental observations, there was more sodium influx at rest when the cell was experiencing mild acidosis than when the resting cell was at normal pH. This was due, at least in part, to increased flux through NHE and NBC. During acidosis there was decreased NCX flux in both the simulated and experimental preparations, but this is not surprising as the NCX experiences inhibition at lower pH.

### The Sodium-Potassium Exchanger and Acidosis

Given that the NaK is one of the dominant sodium flux sources and that it is inhibited by low 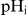, we wanted to examine the effects of removing this acidotic inhibition during reperfusion. For this simulation, our model was modified such that a separate variable for intracellular pH was created and incorporated into the equations for calculating NaK flux. During the pre-ischemic and ischemic phases of the simulation, this variable equals the value of the general 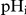 variable. However, during reperfusion, the value of this NaK-specific pH variable is returned to normal, while the rest of the model responds to the values of 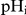 such as those in Figure 5C.

**Figure 8:**
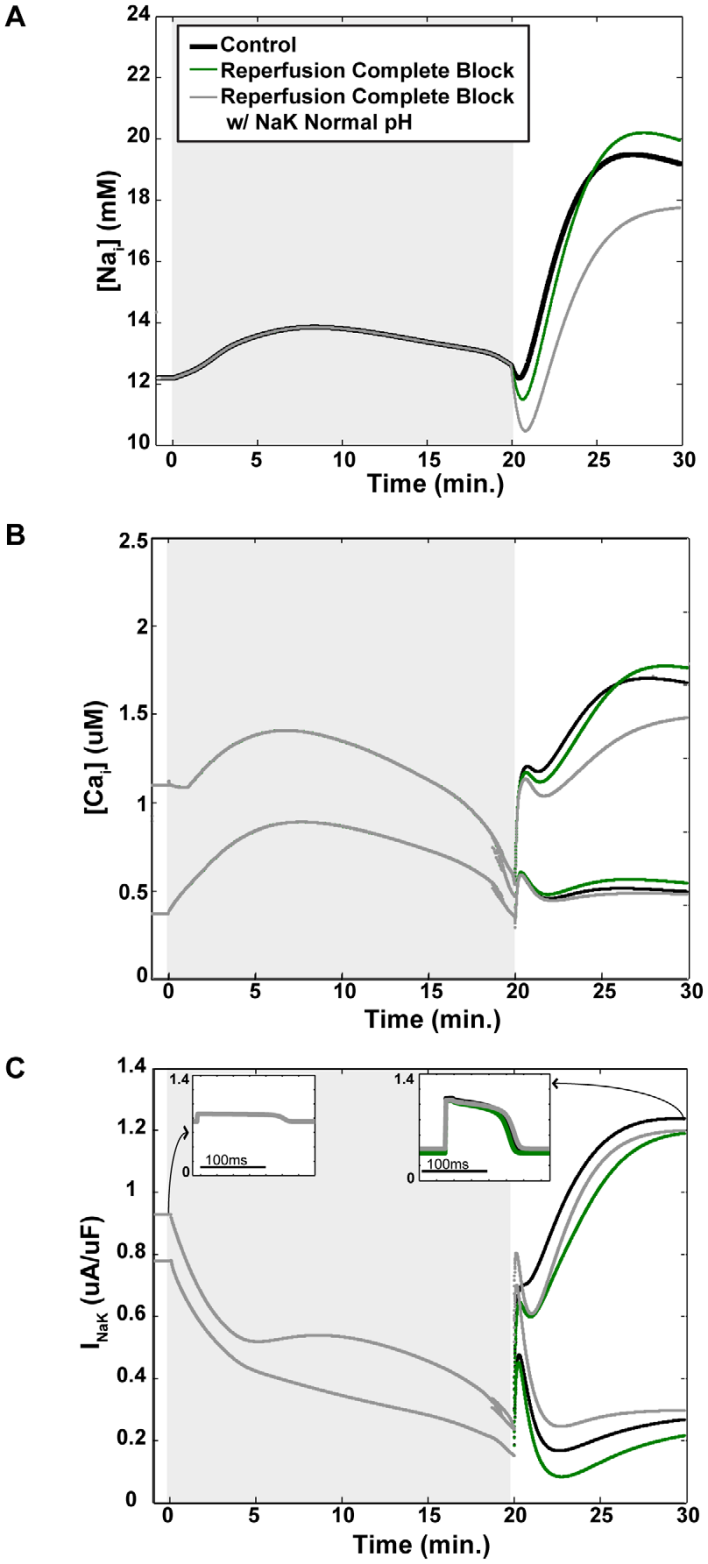
Removing pH-induced inhibition of sodium-potassium exchange decreases sodium and calcium overload during simulated reperfusion. Ischemic phase of simulations denoted by gray shading. The three simulations shown are: Control (with no NHE inhibition) (black), R0 (complete NHE block instituted at the beginning of reperfusion) (green), and R0, but with the NaK responding to a normal pH as opposed to an acidotic pH being sensed by the rest of the subcellular components (gray). Intracellular sodium concentrations are shown in (A), maximum and minimum intracellular calcium concentrations are shown in (B), and maximum and minimum current through the sodium-potassium exchanger (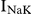) during each beat are shown in (C). If the sodium-potassium exchanger is not inhibited by intracellular acidosis, significant reductions in sodium and calcium overload during reperfusion are observed, due to increased ability of the exchanger to remove sodium from the cell. The left inset in (C) shows 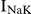 over 150 ms, plotted on the same vertical axis as the main figure, during the last beat prior to the onset of ischemia. The right inset shows current traces, on the same scale, during the last beat of the simulation.

We repeated the R0 simulation with this NaK-specific pH variable. The effects on sodium and calcium concentrations can be seen in Figures 8A and 8B, respectively. Removing acidosis-induced NaK inhibition reduced the peak [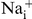] over 2 mM to just under 18 mM and the peak [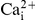] was reduced to 0.00125 mM.

Figure 8C compares the maximum and minimum values of 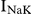 during each beat from the control and R0 simulations to the modified R0 simulation with acidotic inhibition removed. The two insets capture 150 ms, plotted on the same vertical axis as the main figure, at the end of pre-ischemia (left) and at the end of reperfusion (right). If the NaK is not impaired by acidosis during reperfusion, it experiences greater minimum current. Maximum current is still suppressed during early reperfusion, but improves during middle to late reperfusion.

## Discussion

We have developed a model that is capable of simulating the key initial events of myocardial reperfusion: the washout of acidotic and hyperkalemic extracellular fluid, restoring a proton gradient leading to intracellular sodium and calcium overload. A previous model [8] was able to simulate the creation of a proton gradient upon reperfusion, leading to sodium and calcium overload, but that model was developed to simulate respiratory acidosis, not ischemia. As such, the intra- and extracellular pH regulation systems were not fully coupled (i.e. flux of protons and hydroxide through the acid exchangers did not have any effect on the extracellular pH). In addition, our new model incorporates pH sensitivity of the NaK, which was not included in the CS model. Given the important role that phosphometabolites such as ATP play in the development of ischemic pathology, which affects reperfusion events, including the flux of sodium and calcium, we sought to incorporate the ATP dependence of various cellular components into our model. Finally, we wanted to represent dynamic concentrations of as many extracellular species as possible. In particular, modeling dynamic extracellular potassium is important, as it affects sodium concentration via the NaK, as well as the depolarized resting membrane potential that develops during ischemia.

NHE inhibitors reduce the influx of sodium through the NHE when a proton gradient is restored upon reperfusion. However, NHE inhibitors also reduce the efflux of protons through this same exchanger [4], which is a significant means of proton removal from cardiac cells [35]. Given the role that sodium overload plays in the pathophysiology of reperfusion injury, NHE inhibition would appear to be a viable therapy. NHE inhibitors have had some success at reducing the incidence of reperfusion arrhythmias in animal studies [4], as well as reducing the concentration of intracellular sodium when given prior to the onset of ischemia [33]. However, as discussed earlier, NHE inhibitors have not demonstrated any real utility in the clinical setting.

One possible reason for the lack of clinical efficacy is that NHE inhibition may not in fact produce the desired reductions in sodium and calcium overloads. Our simulation results indicate that NHE inhibition, when applied with varying degrees and at various time points during the ischemia-reperfusion event, has a relatively minor impact on the development of sodium and calcium overload during reperfusion. This has been observed experimentally: in a study of rat myocytes exposed to anoxic conditions or metabolic inhibition, NHE inhibition failed to attenuate sodium and calcium overload [36].

In our simulations, the scenarios that produced the lowest peak and mean sodium concentrations during reperfusion were those that exhibited the lowest sodium concentrations at the end of ischemia (Table 2 and Figures 6A and B). NHE inhibition during or prior to the onset of ischemia reduces the intracellular sodium load by the time reperfusion occurs, which appears to confer some benefit in terms of how much sodium accumulates when the proton gradient is restored. This is consistent with what was observed in the aforementioned guinea pig ischemia experiment [33], where inhibiting the NHE prior to and during ischemia suppressed sodium load during the ischemic phase and attenuated (although did not abolish) sodium overload upon reperfusion.

However, the amount of sodium overload during reperfusion is not only dependent upon the concentrations at the end of ischemia, as can be seen by examining the normalized ratios in Table 2. These ratios highlight the interesting fact that the peak sodium concentrations during reperfusion are all similar (within 0.87 mM), despite a wider range of concentrations at the beginning of reperfusion (2.78 mM).

pH-related dynamics appear to play a significant role in setting the approximate peak sodium concentration during reperfusion. While the peak sodium (and calcium) overloads are similar in all simulations, these overloads are clearly the most severe in the R0 simulation, which also manifested the lowest mean and 10-minute reperfusion 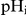 values (Table 4). When complete NHE inhibition was instituted at earlier time points (M0 and I0 simulations), more pH recovery was permitted during reperfusion. This appears to be due to the fact that lower end-ischemia sodium concentrations favor NHE flux, partially compensating for the imposed NHE inhibition.

Examining the two simulations in which NHE inhibition began at reperfusion is helpful, in that prior to the onset of inhibition all simulations experienced the same history. As NHE inhibition severity is increased from 50 percent (R50) to 100 percent (R0), the peak sodium concentrations during reperfusion increased from 19.71 to 20.21 mM (Table 2) and mean 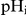 decreased from 7.05 to 6.99 (Table 4), suggesting that there is a relationship between sodium overload and the amount of pH recovery that is permitted. This relationship is the opposite of what one would expect if thinking only in terms of the NHE, which should reduce pH recovery but also reduce sodium overload when inhibited. Therefore, pH-dependent effects on other subcellular components must be responsible for the persistent sodium overload. It should be noted that this relationship does not always hold in the other simulations where NHE inhibition starts at earlier time points. This is largely due to the lower end-ischemic values of [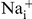] in these simulations.

Given the counterintuitive relationship between pH recovery and sodium overload, the next question we asked was which subcellular components appear to be responsible? There are 8 components though which sodium moves in and out of the cell in our model, and their relative contributions in each simulation are displayed in Figure 7 and Tables 5 and 6. The two biggest contributors are the NCX and NaK, transporting approximately 29 and 47 percent of the total moles of sodium during reperfusion (control simulation), respectively. Both of these exchangers are sensitive to changes in 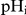 in that they are inhibited by acidosis. Examination of the mean sodium concentrations during reperfusion in Table 2 and the total number of sodium ions moved in Table 5 reveals that NCX (which moves more sodium into the cell than out) is not likely to be responsible for the sodium overloads observed during reperfusion. During the R-series simulations, where mean sodium concentration increases as the severity of NHE inhibition increases, total NCX ion movement decreases. The opposite is true of the M- and I-series simulations, where increasing NHE inhibition is associated with decreased sodium concentration but increased NCX current. The NCX, although it is inhibited to some degree by acidosis, appears to be largely responding to changes in sodium concentration. Examination of the mean 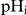 values in Table 4 and the number of moles moved in Table 5 reveals that the two are sometimes directly related, but at other times the relationship is inverse.

On the other hand, NaK current, as presented in Table 5, is always directly related to mean pH (Table 4) but not always with mean sodium concentration (Table 2). Suppression of pH recovery appears to inhibit NaK activity, thereby reducing the number of sodium ions removed from the cell during reperfusion. This theory appears valid if examining the results of the R-series experiments, but appears to break down when examining the mean and peak sodium concentrations of the other simulations, where peak and mean sodium overload decrease and pH recovery decreases. However, in these simulations there are also progressively decreasing end-ischemic sodium concentrations. If one considers the amount of sodium that enters the cell during reperfusion in relation to what was present at the end of ischemia (as represented by the ratios in Table 2), suppressed 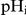, and NaK current correlate with sodium overload. When we removed the effects of pH-dependent inhibition upon the NaK during reperfusion, we observed significant improvements in terms of sodium and calcium overload (Figures 8A and 8B, respectively) that appear to be mediated by increased NaK current (Figure 8C).

It is interesting to compare these findings with those from another modeling study in which acid-loading was employed as an analog to ischemia-reperfusion [18]. As in our study, Ch'en *et al*, found that NHE inhibition impaired intracellular pH recovery. However, sodium overload following the acid load was reduced, not increased, as was the case with our NHE inhibition simulations. This fundamentally different behavior is largely the result of adding pH dependence to the NaK, which appears to be absent in the older model.

The only other sodium flux source that appeared to play a role in sodium overload during reperfusion was the NBC, which moves sodium, as well as bicarbonate, into the cell. We observed that as the strength of NHE inhibition was increased, which suppresses pH recovery, intracellular bicarbonate concentrations were decreased (Table 7) and NBC flux increased (Table 5). Keeping intracellular pH low would be expected to produce lower bicarbonate concentrations, leading to a gradient that favors NBC import of bicarbonate, and sodium along with it.

**Table 7:** Intracellular bicarbonate.

	Control	R50	R0	M50	M0	I50	I0
End 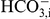	6.85	6.37	5.53	6.44	5.97	6.44	5.97
Mean 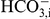	5.72	5.22	4.32	5.40	5.03	5.41	4.98

Concentrations in mM.

Given the apparent failure of NHE inhibition to attenuate sodium overload during reperfusion, and the prominent role that NaK plays in intracellular sodium regulation, we investigated whether the NaK could be manipulated in order to improve sodium overload. To this end, we performed two additional simulations, the results of which are provided in Figure S2 in Text S1. In the first, we doubled the amount of NaK current starting at the moment of initial reperfusion. This produced a dramatic reduction in overload during reperfusion, with a peak concentration of about 12 mM. However, there is no known way to directly increase NaK activity in such a selective fashion. NaK activity can likely be stimulated indirectly by making more ATP available. In the second simulation, we allowed ATP concentration to recover to the preischemic value during reperfusion, as opposed to limiting recovery to 40 percent of the preischemic value. This approach also produced a dramatic reduction in sodium overload, resulting in a peak concentration similar to that observed in the first simulation. Delivery of ATP encapsulated in liposomes [37] has been shown to reduce the extent of ischemia-reperfusion injury *in vitro* and *in vivo*. Whether such improvement was the result of increased NaK activity remains to be shown, but the prospect appears promising nonetheless.

NHE inhibition and the resulting suppression of pH recovery during reperfusion appears to worsen sodium load, and in simulations where we removed pH-related inhibition of the NaK, there was a reduction in sodium load relative to control (Figure 8A). However, there was still a substantial overload during reperfusion (gray line). This, combined with the results of simulations discussed in the preceding paragraph and Figure S2 in Text S1, suggest another possible reason why NHE inhibition has thus far failed to be effective. Not only does NHE inhibition appear to adversely affect multiple cellular components by decreasing pH, but it may be the case that NHE inhibition alone is not sufficient and that ATP manipulation is a more effective approach.

Earlier it was noted that there are several possible reasons for the failure thus far in developing an effective therapy for the treatment of ischemia-reperfusion injury. Within the context of NHE inhibition, it is important to remember that many of the subjects involved in clinical trials are afflicted with conditions such as cardiac hypertrophy and heart failure. There is evidence linking increased NHE activity to heart failure, both in the clinical setting and in experimental models [35]. In addition, there is evidence suggesting that NHE inhibition therapy can reverse cardiac hypertrophy in animal models. It is reasonable to expect that, in diseased cardiomyocytes with increased NHE activity, this exchanger would play a more prominent role in sodium and pH regulation. This may prove to be a liability for patients afflicted with heart failure if the conclusions of this study are true.

The preceding discussion concerning sodium and calcium overload has been of a qualitative nature, but a logical followup question is exactly how much sodium and/or calcium overload is pathological? This is a difficult question to answer, as it likely depends on the state of many other components in the cell [38]. In addition, profiles of experimentally measured sodium and calcium concentrations in ischemic and reperfused myocardium vary depending on experimental technique [33] and species, among other factors. However, some light can be shed by examining recent results examining relationships between sodium, calcium, and cardiac dysfunction in guinea pig cells [39]. In this study, ouabain was used to inhibit NaK function, resulting in elevated intracellular sodium concentrations. When cells were paced at a rate of 1 Hz, [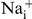] rose to a concentration of approximately 8 mM within 5 minutes of ouabain treatment at a concentration of 0.1 uM, and remained at about 8 mM for the rest of the experiment. When a concentration of 0.5 uM was used, [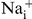] reached a concentration between 8 and 9 mM within 5 minutes, further climbing to a concentration of about 15 mM 3 minutes later. Further experiments were performed using ouabain at a concentration of 0.25 uM, so the sodium concentrations in these experiments were likely between 8 and 15 mM. In the same study, peak systolic calcium concentrations reached approximately 2.5 and 3 uM 5 and 8 minutes after 0.25 uM ouabain treatment, respectively (compared to approximately 1.4 uM in control). At the same time, the probability of an action potential being triggered by a delayed afterdepolarization was in excess of 0.3 (compared to less than 0.1 for control), the rate of oxygen consumption nearly doubled, and reduced NADH availability fell to approximately one-third of control levels.

Again, these numbers provide only a rough guide, and the results of the ouabain study did not occur in the setting of true ischemia. However, what we found particularly interesting in the present study was that at best NHE inhibition resulted in a very small improvement in peak [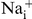] during reperfusion (less than 1 percent), and that this adverse outcome was the result of inhibiting intracellular pH recovery.

### Limitations

The model presented here was used for the simulation of abrupt, complete stop-flow ischemia and abrupt-onset reperfusion. However, with some relatively simple modifications to the simulation protocols, it is possible to simulate ischemia of varying severity and following different time courses (e.g. episodes of ischemia punctuated with intervals of reperfusion).

This model is by no means an attempt to capture all of the mechanisms of ischemic pathophysiology. For example, 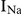 [40], [41] and 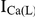 [41], [42] have been reported to be modified by acidosis. However, these sensitivities are not represented in the model, as they did not change model performance in a significant way and because of possible confounding factors in the experimental evidence with regard to the 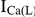 data. A discussion of the issues relating to the 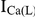 current can be found in [8], [43]. Also, there has been work centered around the role of other components and processes in the development of reperfusion arrhythmias (for example, see [44] and [45]). Explicit mitochondrial physiology would be an interesting addition to the model, particularly in the context of mitochondrial NHE function and proton and calcium homeostasis. In the current version of the model, the equations dictating phosphometabolite concentrations serve as a proxy for mitochondrial failure, a choice we made based upon computational time considerations. However, we expect to incorporate more detailed mitochondrial physiology in the future. Despite the many facets of ischemia physiology that are not included in our model, we believe that it is an important step towards a comprehensive ischemia-reperfusion model, and one that reproduces electrical and chemical changes that have been seen *in vitro*. Reactive oxygen species generation is also believed to play a major role, as is apoptosis. These will be interesting potential additions in the future once suitable mathematical representations are devised, especially if longer reperfusion times are to be modeled, where apoptosis likely plays a more significant role.

Comparison of our simulation results with experimental data in Figure 4 reveals some discrepancies in behavior. The level of extracellular acidosis produced by our model under these simulation conditions is more profound than what is typically observed. In addition, the initial conditions and parameters that we have chosen yield higher sodium concentrations during ischemia and a slower rate of sodium accumulation during reperfusion than observed in the cited experiment. These are the result of choices we have made in tuning many parameters to fit data from many different groups, performed under different conditions and at different points in time. For example, an improved match to the cited sodium data can be achieved with the model, but at the expense of a decreased fit to extracellular pH and potassium data. While there is still room for improvement, we believe that this model provides a good fit to phenomena that occur during myocardial reperfusion and can yield useful insight into this complex problem.

Many calcium-handling components are sensitive to changes in cellular pH, and some of these relationships are represented in our model. In addition, our model includes ATP-dependencies of components that directly and indirectly affect sodium and calcium balance, such as the NaK, SERCA, and ATP-inactivated potassium efflux channels. The importance of these influences have been alluded to above and are being explored in current work.

### Conclusion

The pathophysiology of ischemia-reperfusion injury is very complex, taking place within the context of a system that is highly coupled with nonlinear relationships between many of the components. Because such systems frequently exhibit nonintuitive behavior, we believe that the model developed in this study will prove useful in illuminating underlying mechanisms. For example, here we have used the model to propose a possible explanation for why NHE inhibition, a seemingly straightforward approach to limiting sodium-mediated reperfusion injury, has shown no meaningful clinical efficacy to date, and what efficacy has been observed requires the administration of NHE blockers prior to the onset of ischemia. Our investigations indicate that NHE inhibition can paradoxically exacerbate sodium and calcium overload. NHE inhibition suppresses intracellular pH recovery during reperfusion, which decreases the ability of the sodium-potassium exchanger to remove sodium from the cell and favors influx of sodium through the sodium-bicarbonate symporter. Most of any observed benefit of NHE inhibition appears to come from decreased sodium load resulting from reduced NHE activity prior to the onset of ischemia. Suppressing pH recovery during reperfusion is also likely to negatively impact other cellular systems, such as those responsible for handling calcium, that we have not examined here.

## Supporting Information

Text S1 Contains names and constant values (where applicable) for all model variables, all model equations, additional information regarding experiments used to inform model parameters, a summary of the ischemia-reperfusion simulation protocol, and additional figures pertaining to simulations relevant to model validation.
(PDF)

## References

[1] Ferdinandy P, Schulz R, Baxter GF (2007). Interaction of cardiovascular risk factors with myocardial ischemia/reperfusion injury, preconditioning, and postconditioning.. Pharmacol Rev.

[2] Yellon DM, Hausenloy DJ (2007). Myocardial reperfusion injury.. N Engl J Med.

[3] Dirksen MT, Laarman GJ, Simoons ML, Duncker DJGM (2007). Reperfusion injury in humans: a review of clinical trials on reperfusion injury inhibitory strategies.. Cardiovasc Res.

[4] Moens AL, Claeys MJ, Timmermans JP, Vrints CJ (2005). Myocardial ischemia/reperfusion-injury, a clinical view on a complex pathophysiological process.. Int J Cardiol.

[5] Kleber AG (1983). Resting membrane potential, extracellular potassium activity, and intracellular sodium activity during acute global ischemia in isolated perfused guinea pig hearts.. Circ Res.

[6] Terkildsen JR, Crampin EJ, Smith NP (2007). The balance between inactivation and activation of the na+-k+ pump underlies the triphasic accumulation of extracellular k+ during myocardial ischemia.. Am J Physiol Heart Circ Physiol.

[7] Cascio WE, Yan GX, Kléber AG (1992). Early changes in extracellular potassium in ischemic rabbit myocardium. the role of extracellular carbon dioxide accumulation and diffusion.. Circ Res.

[8] Crampin EJ, Smith NP (2006). A dynamic model of excitation-contraction coupling during acidosis in cardiac ventricular myocytes.. Biophys J.

[9] Lakireddy V, Baweja P, Syed A, Bub G, Boutjdir M (2005). Contrasting effects of ischemia on the kinetics of membrane voltage and intracellular calcium transient underlie electrical alternans.. Am J Physiol Heart Circ Physiol.

[10] Orchard CH, Cingolani HE (1994). Acidosis and arrhythmias in cardiac muscle.. Cardiovasc Res.

[11] Orchard CH, Kentish JC (1990). Effects of changes of ph on the contractile function of cardiac muscle.. Am J Physiol.

[12] Mandel F, Kranias EG, Grassi de Gende A, Sumida M, Schwartz A (1982). The effect of ph on the transient-state kinetics of ca2+-mg2+-atpase of cardiac sarcoplasmic reticulum. a comparison with skeletal sarcoplasmic reticulum.. Circ Res.

[13] Tran K, Smith NP, Loiselle DS, Crampin EJ (2009). A thermodynamic model of the cardiac sar-coplasmic/endoplasmic ca(2+) (serca) pump.. Biophys J.

[14] Xu L, Mann G, Meissner G (1996). Regulation of cardiac ca2+ release channel (ryanodine receptor) by ca2+, h+, mg2+, and adenine nucleotides under normal and simulated ischemic conditions.. Circ Res.

[15] Doering AE, Lederer WJ (1993). The mechanism by which cytoplasmic protons inhibit the sodium-calcium exchanger in guinea-pig heart cells.. J Physiol.

[16] Gottlieb RA, Mentzer RM, Sellke FW (2010). Cme multimedia activity: Mechanisms and consequences of cardiac ischemia-reperfusion injury: Insights and evidence to improve outcomes.. Am J Cardiol.

[17] Karmazyn M, Gan XT, Humphreys RA, Yoshida H, Kusumoto K (1999). The myocardial na(+)- h(+) exchange: structure, regulation, and its role in heart disease.. Circ Res.

[18] Ch'en FF, Vaughan-Jones RD, Clarke K, Noble D (1998). Modelling myocardial ischaemia and reperfusion.. Prog Biophys Mol Biol.

[19] Hund TJ, Kucera JP, Otani NF, Rudy Y (2001). Ionic charge conservation and long-term steady state in the luo-rudy dynamic cell model.. Biophys J.

[20] Varghese A, Sell GR (1997). A conservation principle and its e_ect on the formulation of na-ca exchanger current in cardiac cells.. J Theor Biol.

[21] Endresen LP, Hall K, Hoye JS, Myrheim J (2000). A theory for the membrane potential of living cells.. Eur Biophys J.

[22] Vaughan-Jones RD, Wu ML (1990). Extracellular h+ inactivation of na(+)-h+ exchange in the sheep cardiac purkinje fibre.. J Physiol.

[23] Vaughan-Jones RD, Spitzer KW (2002). Role of bicarbonate in the regulation of intracellular ph in the mammalian ventricular myocyte.. Biochem Cell Biol.

[24] Yazawa K, Kameyama A, Yasui K, Li JM, Kameyama M (1997). Atp regulates cardiac ca2+ channel activity via a mechanism independent of protein phosphorylation.. Pugers Arch.

[25] O'Rourke B, Backx PH, Marban E (1992). Phosphorylation-independent modulation of l-type calcium channels by magnesium-nucleotide complexes.. Science.

[26] Michailova A, Saucerman J, Belik ME, McCulloch AD (2005). Modeling regulation of cardiac katp and l-type ca2+ currents by atp, adp, and mg2+.. Biophys J.

[27] Gaur N, Rudy Y, Hool L (2009). Contributions of ion channel currents to ventricular action potential changes and induction of early afterdepolarizations during acute hypoxia.. Circ Res.

[28] Fleet WF, Johnson TA, Graebner CA, Gettes LS (1985). Effect of serial brief ischemic episodes on extracellular k+, ph, and activation in the pig.. Circulation.

[29] Kitakaze M, Takashima S, Funaya H, Minamino T, Node K (1997). Temporary acidosis during reperfusion limits myocardial infarct size in dogs.. Am J Physiol.

[30] Befroy DE, Powell T, Radda GK, Clarke K (1999). Osmotic shock: modulation of contractile function, phi, and ischemic damage in perfused guinea pig heart.. Am J Physiol.

[31] Shigematsu S, Sato T, Abe T, Saikawa T, Sakata T (1995). Pharmacological evidence for the persistent activation of atp-sensitive k+ channels in early phase of reperfusion and its protective role against myocardial stunning.. Circulation.

[32] Sato R, Sakamoto K, Yamazaki J, Nagao T (2002). Differences in protective profiles of diltiazem isomers in ischemic and reperfused guinea pig hearts.. Eur J Pharmacol.

[33] An J, Varadarajan SG, Camara A, Chen Q, Novalija E (2001). Blocking na(+)/h(+) exchange reduces [na(+)](i) and [ca(2+)](i) load after ischemia and improves function in intact hearts.. Am J Physiol Heart Circ Physiol.

[34] Bers DM, Barry WH, Despa S (2003). Intracellular na+ regulation in cardiac myocytes.. Cardiovasc Res.

[35] Karmazyn M, Kilić A, Javadov S (2008). The role of nhe-1 in myocardial hypertrophy and remodelling.. J Mol Cell Cardiol.

[36] Russ U, Balser C, Scholz W, Albus U, Lang HJ (1996). Effects of the na+/h+-exchange inhibitor hoe 642 on intracellular ph, calcium and sodium in isolated rat ventricular myocytes.. Pugers Arch.

[37] Levchenko TS, Hartner WC, Verma DD, Bernstein EA, Torchilin VP (2010). Atp-loaded liposomes for targeted treatment in models of myocardial ischemia.. Methods Mol Biol.

[38] Allen DG, Orchard CH (1987). Myocardial contractile function during ischemia and hypoxia.. Circ Res.

[39] Liu T, Brown DA, O'Rourke B (2010). Role of mitochondrial dysfunction in cardiac glycoside toxicity.. J Mol Cell Cardiol.

[40] Kagiyama Y, Hill JL, Gettes LS (1982). Interaction of acidosis and increased extracellular potassium on action potential characteristics and conduction in guinea pig ventricular muscle.. Circ Res.

[41] Shaw RM, Rudy Y (1997). Electrophysiologic effects of acute myocardial ischemia: a theoretical study of altered cell excitability and action potential duration.. Cardiovasc Res.

[42] Irisawa H, Sato R (1986). Intra- and extracellular actions of proton on the calcium current of isolated guinea pig ventricular cells.. Circ Res.

[43] Hulme JT, Orchard CH (1998). Effect of acidosis on ca2+ uptake and release by sarcoplasmic reticulum of intact rat ventricular myocytes.. Am J Physiol.

[44] Akar FG, Aon MA, Tomaselli GF, O'Rourke B (2005). The mitochondrial origin of postischemic arrhythmias.. J Clin Invest.

[45] Aiello EA, Jabr RI, Cole WC (1995). Arrhythmia and delayed recovery of cardiac action potential during reperfusion after ischemia. role of oxygen radical-induced no-reow phenomenon.. Circ Res.

