# Supplementary material for: NHE Inhibition Does Not Improve Na+ or Ca2+ Overload During Reperfusion: Using Modeling to Illuminate the Mechanisms Underlying a Therapeutic Failure
Source: PLoS Comput Biol. 2011 Oct 20;7(10):e1002241. doi: 10.1371/journal.pcbi.1002241 (PMC3197652; doi:10.1371/journal.pcbi.1002241)
Supplement: Text S1 — Contains names and constant values (where applicable) for all model variables, all model equations, additional information regarding experiments used to inform model parameters, a summary of the ischemia-reperfusion simulation protocol, and additional figures pertaining to simulations relevant to model validation. (PDF) [file pcbi.1002241.s001.pdf]

Text S1

Supplementary Material for "NHE Inhibition Does Not Improve Na^+ or Ca^{2+} Overload During Reperfusion: Using Modeling to Illuminate the Mechanisms Underlying a Therapeutic Failure."

Byron N. Roberts and David J. Christini

Symbols and Values

Abbreviation	Parameter	Units	Constant Value
A_{cap}	capacitive area	cm^2	0.0001534 [1]
aa_{AE}	transition rate (AE)	N/A	N/A
aa_{CHE}	transition rate (CHE)	N/A	N/A
aa_{NBC}	transition rate (NBC)	N/A	N/A
aa_{NHE}	transition rate (NHE)	N/A	N/A
[ADP]	ADP concentration	mM	N/A
α_h	opening rate constant for h gate	ms^{-1}	N/A
α_j	opening rate constant for j gate	ms^{-1}	N/A
α_{K1}	rate constant for I_{K1} inactivation	ms^{-1}	N/A
α_m	opening rate constant for m gate	ms^{-1}	N/A
α_p	opening rate constant for p gate	ms^{-1}	N/A
α^{+}_{NaK1}	forward rate constant (NaK)	s^{-1}	N/A
α^{-}_{NaK1}	reverse rate constant (NaK)	s^{-1}	N/A
α^{+}_{NaK2}	forward rate constant (NaK)	s^{-1}	N/A
α^{-}_{NaK2}	reverse rate constant (NaK)	s^{-1}	N/A
α^{+}_{NaK3}	forward rate constant (NaK)	s^{-1}	N/A
α^{-}_{NaK3}	reverse rate constant (NaK)	s^{-1}	N/A
α^{+}_{NaK4}	forward rate constant (NaK)	s^{-1}	N/A
α^{-}_{NaK4}	reverse rate constant (NaK)	s^{-1}	N/A
$\alpha^{+}_{1,SERCA}$	apparent forward rate constant	s^{-1}	N/A
$\alpha^{-}_{1,SERCA}$	apparent reverse rate constant	s^{-1}	N/A
$\alpha^{+}_{2,SERCA}$	apparent forward rate constant	s^{-1}	N/A
$\alpha^{-}_{2,SERCA}$	apparent reverse rate constant	s^{-1}	N/A
$\alpha^{+}_{3,SERCA}$	apparent forward rate constant	s^{-1}	N/A
$\alpha^{-}_{3,SERCA}$	apparent reverse rate constant	s^{-1}	N/A
[AMP]	AMP concentration	mM	N/A
$[Anion^{-}]$	concentration of conjugate anions due to metabolic acidosis	mM	N/A
[ATP]	ATP concentration	mM	N/A
$\widetilde{[ATP]}$	apparent concentration of enzyme-bound ion (NaK)	N/A	N/A
b	$I_{Ca,t}$ activation gate	N/A	N/A
b_∞	steady state value for b gate	N/A	N/A
bb_{AE}	transition rate (AE)	N/A	N/A
bb_{CHE}	transition rate (CHE)	N/A	N/A
bb_{NBC}	transition rate (NBC)	N/A	N/A

Table S1: Note: numbers in brackets refer to citations in the biblography at the end of this supplement.

Abbreviation	Parameter	Units	Constant Value
bb_{NHE}	transition rate (NHE)	N/A	N/A
β_{cai}	Ca^{2+} buffering	N/A	N/A
β_{CMDN}	calmodulin buffering power	N/A	N/A
β_{CSQN}	calsequestrin buffering power	N/A	N/A
β_{e}	extracellular intrinsic (non-CO_2) buffering capacity	N/A	N/A
β_{h}	closing rate constant for h gate	ms^{-1}	N/A
β_{i}	intracellular intrinsic (non-CO_2) buffering capacity	N/A	N/A
β_{j}	closing rate constant for j gate	ms^{-1}	N/A
β_{K1}	rate constant for I_{K1} inactivation	ms^{-1}	N/A
β_{m}	closing rate constant for m gate	ms^{-1}	N/A
β_{p}	closing rate constant for p gate	ms^{-1}	N/A
β_{TRPN}	troponin buffering powering power	N/A	N/A
c_{ADP}	closed prob. because two ADP molecules not bound to one subunit	N/A	N/A
$[Ca_i^{2+}]$	intracellular Ca^{2+} concentration	mM	N/A
$[Ca_o^{2+}]$	extracellular Ca^{2+} concentration	mM	N/A
$\widetilde{Ca}_{i,SERCA}$	apparent Ca^{2+} binding to SERCA in myoplasm	N/A	N/A
$\widetilde{Ca}_{SR,SERCA}$	apparent Ca^{2+} binding to SERCA in SR	N/A	N/A
cc_{AE}	transition rate (AE)	N/A	N/A
cc_{CHE}	transition rate (CHE)	N/A	N/A
cc_{NBC}	transition rate (NBC)	N/A	N/A
cc_{NHE}	transition rate (NHE)	N/A	N/A
$[Cl_e^-]$	extracellular chloride concentration	mM	146.7 [2]
$[Cl_i^-]$	intracellular chloride concentration	mM	N/A
C_m	membrane capacitance	uF/cm^2	1.0 [1]
[CMDN]	concentration of Ca^{2+} bound to calmodulin	mM	N/A
$[\overline{CMDN}]$	max $[Ca^{2+}]$ bound to calmodulin	mM	0.050 [1]
$[CO_{2,e}]$	extracellular carbon dioxide concentration	mM	N/A
CO_{2sol}	carbon dioxide solubility	mM	0.03253 [1]
$[CO_{2,i}]$	intracellular carbon dioxide concentration	mM	N/A
[Cr]	creatine concentration	mM	N/A
[CSQN]	concentration of Ca^{2+} bound to calsequestrin	mM	N/A
csqnth	threshold for Ca^{2+}release from CSQN due to JSR overload	mM	8.75 [1]
$[\overline{CSQN}]$	max $[Ca^{2+}]$ bound to calsequestrin	mM	10.0 [1]
cyc_{NaK}	clockwise steady-state cycle rate (NaK)	s^{-1}	N/A
cyc_{SERCA}	SERCA cycling rate	s^{-1}	N/A
C_0	constant used in calculating voltage by charge diff. method	mM	N/A
c1	scaling factor for I_{NaCa}	uA/uF	0.00015
c2	half-saturation concentration for NaCa exchanger	mM	0.0001 [1]
d	$I_{Ca(L)}$ activation gate	N/A	N/A
d_{∞}	steady state value for d gate	N/A	N/A
dd_{AE}	transition rate (AE)	N/A	N/A
dd_{CHE}	transition rate (CHE)	N/A	N/A
dd_{NBC}	transition rate (NBC)	N/A	N/A
dd_{NHE}	transition rate (NHE)	N/A	N/A
Δt	time step	ms	0.005
$density_{NaK}$	density of NaK pump enzymes in sarcolemma	um^{-2}	7450.0 [3]
$dI_{Ca,total}$	rate of Ca^{2+} flux across membrane	uA/(uF*ms)	N/A
dvdtnew	change in V between time steps	mV/ms	N/A
E_{Ca}	Ca^{2+} reversal potential	mV	N/A
E_K	K^+ reversal potential	mV	N/A

Abbreviation	Parameter	Units	Constant Value
E_{Ks}	K^+ reversal potential for I_{Ks}	mV	N/A
E_{Na}	Na^+ reversal potential	mV	N/A
F	Faraday's constant	C/mol	96485.0 [1]
f	$I_{Ca(L)}$ inactivation gate	N/A	N/A
f_∞	steady state value for f gate	N/A	N/A
f_{ADP}	open probability due to ADP binding	N/A	N/A
f'_{ADP}	open probability due to ADP binding	N/A	N/A
f_{ATP}	fraction of channels open because no ATP binding	N/A	N/A
f_{Ca}	$I_{Ca(L)}$ Ca^{2+} dependent inactivation gate	N/A	N/A
$f_{Ca,ATP}$	relative $I_{Ca(L)}$ channel availability	N/A	N/A
$f_{K(ATP)}$	open probability (no ATP or two ADP bound to subunit)	N/A	N/A
$fac_{H,RYR}$	pH-dependent modification of RYR activity	N/A	N/A
fac_{NaCa}	pH-dependent modification of NaCa activity	N/A	N/A
factorNaKdel	determines voltage dependence of binding reactions	N/A	-0.17952 [3]
flag	Boolean variable used to test for dvdtmax	N/A	N/A
g	$I_{Ca,t}$ inactivation gate	N/A	N/A
g_∞	steady state value for g gate	N/A	N/A
$g_{Ca,b}$	max. $I_{Ca,b}$ conductance	mS/uF	0.003016 [1]
$g_{Ca,t}$	max. $I_{Ca,t}$ conductance	mS/uF	0.05 [1]
$g_{K(ATP)}$	$I_{K(ATP)}$ channel conductance	mS/uF	N/A
g_{Kp}	I_{Kp} channel conductance	mS/uF	0.00552 [1]
g_{Kr}	I_{Kr} channel conductance	mS/uF	N/A
g_{Ks}	I_{Ks} channel conductance	mS/uF	N/A
g_{K1}	I_{K1} channel conductance	mS/uF	N/A
g_{maxrel}	max. rate constant of Ca^{2+} release from JSR	ms^{-1}	37.5 [1]
g_{Na}	max. I_{Na} conductance	mS/uF	16.0 (14.4 ischemia) [4]
$g_{Na,b}$	max. $I_{Na,b}$ conductance	mS/uF	0.002 [1]
$g_{relbarjsrol}$	rate constant of Ca^{2+} release from JSR due to overload	ms^{-1}	4.0 [1]
$g_{reljsrol}$	rate of constant Ca^{2+} release from JSR due to CICR	ms^{-1}	N/A
gacai	intracellular Ca^{2+} activity coefficient	N/A	1.0 [1]
gacao	extracellular Ca^{2+} activity coefficient	N/A	0.341 [1]
gaki	intracellular K^+ activity coefficient	N/A	0.75 [1]
gako	extracellular K^+ activity coefficient	N/A	0.75 [1]
gammas	energy barrier controlling voltage dependence of NaCa	N/A	0.12 [1]
ganai	intracellular Na^+ activity coefficient	N/A	0.75 [1]
ganao	extracellular Na^+ activity coefficient	N/A	0.75 [1]
h	I_{Na} inactivation gate	N/A	N/A
h_∞	steady state value for h gate	N/A	N/A
$[H_e^+]$	extracellular proton concentration	mM	N/A
$[H_i^+]$	intracellular proton concentration	mM	N/A
$\widetilde{H}_{SERCA}$	apparent proton binding to SERCA in SR	N/A	N/A
$\widetilde{H}_{i,SERCA}$	apparent proton binding to SERCA in myoplasm	N/A	N/A
$\widetilde{H}_{1,SERCA}$	apparent proton binding to SERCA in myoplasm	N/A	N/A
$\widetilde{H}_{SR,SERCA}$	apparent proton binding to SERCA in SR	N/A	N/A
$[HCO^-_{3,e}]$	extracellular bicarbonate concentration	mM	N/A
$[HCO^-_{3,i}]$	intracellular bicarbonate concentration	mM	N/A
$[Hib_{ref}]$	concentration of protons bound to intrinsic buffers at start	mM	N/A
$[Hib_1]$	concentration of protons bound to first generic intrinsic buffer	mM	N/A
$[Hib_2]$	concentration of protons bound to second generic intrinsic buffer	mM	N/A

Abbreviation	Parameter	Units	Constant Value
$\bar{I}_{Ca}$	max. Ca^{2+} current through $I_{Ca(L)}$	uA/uF	N/A
$I_{Ca,b}$	background Ca^{2+} current	uA/uF	N/A
$I_{Ca(L)}$	L-type Ca^{2+} current	uA/uF	N/A
$I_{Ca(L),K}$	K^+ flux through $I_{Ca(L)}$	uA/uF	N/A
$I_{Ca(L),Na}$	Na^+ flux through $I_{Ca(L)}$	uA/uF	N/A
$I_{Ca,t}$	T-type Ca^{2+} current	uA/uF	N/A
$I_{Ca,total}$	total Ca^{2+} flux across cell membrane	uA/uF	N/A
I_{Cl}	Cl^- current	uA/uF	N/A
$I_{Cl,total}$	total Cl^- flux across cell membrane	uA/uF	N/A
$\bar{I}_{K}$	max. K^+ current through $I_{Ca(L)}$	uA/uF	N/A
$I_{K(ATP)}$	ATP-inactivated K^+ current	uA/uF	N/A
I_{Kp}	plateau K^+ current	uA/uF	N/A
I_{Kr}	rapid delayed K^+ rectifier current	uA/uF	N/A
I_{Ks}	slow delayed K^+ rectifier current	uA/uF	N/A
I_{K1}	time-independent K^+ rectifier current	uA/uF	N/A
$I_{K,total}$	total K^+ flux across cell membrane	uA/uF	N/A
I_{leak}	Ca^{2+} leakage from NSR to myoplasm	mM/ms	N/A
I_{Na}	fast inward sodium current	uA/uF	N/A
$\bar{I}_{Na}$	max. Na^+ current through $I_{Ca(L)}$	uA/uF	N/A
$I_{Na,b}$	background sodium current	uA/uF	N/A
I_{NaCa}	current through NaCa exchanger	uA/uF	N/A
I_{NaK}	current through the NaK pump	uA/cm^2	N/A
$I_{Na,l}$	late sodium current	uA/uf	N/A
$I_{Na,total}$	total Na^+ flux across cell membrane	uA/uF	N/A
$I_{p(Ca)}$	Ca^{2+} pump current	uA/uF	N/A
$\bar{I}_{p(Ca)}$	max. $I_{p(Ca)}$ current	uA/uF	1.65
$I_{relcicr}$	Ca^{2+} release from JSR to myoplasm due to CICR	mM/ms	N/A
$I_{reljsrol}$	Ca^{2+} release from JSR to myoplasm due to overload	mM/ms	N/A
I_{stim}	stimulus current	uA/uF	-80.0 [1]
I_{tr}	translocation of Ca^{2+} from NSR to JSR	mM/ms	N/A
$\bar{I}_{up}$	max. flux through SERCA	mM/ms	0.00875 [1]
$[ib_1]$	concentration of first generic intrinsic buffer	mM	84.22 [1]
$[ib_2]$	concentration of second generic intrinsic buffer	mM	29.38 [1]
j	I_{Na} inactivation gate	N/A	N/A
j_∞	steady state value for j gate	N/A	N/A
[JSR]	Ca^{2+} concentration in JSR	mM	N/A
jsr_{magrel}	magnitude of Ca^{2+} release	N/A	N/A
jsr_{off}	deactivation of Ca^{2+} release from JSR	N/A	N/A
jsr_{on}	activation of Ca^{2+} release from JSR	N/A	N/A
$k_{AE,cl}$	dissociation constant (AE)	mM	983.50 [1]
$k_{AE,hco3}$	dissociation constant (AE)	mM	110.64 [1]
$K_{AE,he}$	dissociation constant (AE)	mM	0.000312 [1]
$K_{AE,hi}$	dissociation constant (AE)	mM	0.0000267 [1]
${k^+}_{AE1}$	forward rate constant (AE)	ms^{-1}	20,789.9 [1]
${k^-}_{AE1}$	reverse rate constant (AE)	ms^{-1}	21,118.2 [1]
${k^+}_{AE2}$	forward rate constant (AE)	ms^{-1}	21,256.54 [1]
${k^-}_{AE2}$	reverse rate constant (AE)	ms^{-1}	21592.21 [1]
k_{ATP}	$k_{1/2}$ for the binding of ATP to $I_{Ca(L)}$ channels	mM	1.4 [5]
$k_{CHE,cl}$	dissociation constant (CHE)	mM	17,970.36 [1]
$k_{CHE,oh}$	dissociation constant (CHE)	mM	0.0008907 [1]

Abbreviation	Parameter	Units	Constant Value
k^{+}_{CHE1}	forward rate constant (CHE)	ms^{-1}	14,975.6 [1]
k^{-}_{CHE1}	reverse rate constant (CHE)	ms^{-1}	257.4 [1]
k^{+}_{CHE2}	forward rate constant (CHE)	ms^{-1}	4,084.9 [1]
k^{-}_{CHE2}	reverse rate constant (CHE)	ms^{-1}	70.20 [1]
$K_{d,Cai,SERCA}$	K_d for Ca^{2+} binding to SERCA in myoplasm	mM	0.91 [6]
$K_{d,CaSR,SERCA}$	K_d for Ca^{2+} binding to SERCA in SERCA	mM	2.24 [6]
$K_{d,H,SERCA}$	K_d for proton binding to SERCA in SR	mM	0.0000724 [6]
$K_{d,Hi,SERCA}$	K_d for proton binding to SERCA in myoplasm	mM^2	0.00354 [6]
$K_{d,HSR,SERCA}$	K_d for proton binding to SERCA in SR	mM^2	0.0000000105 [6]
$K_{d,H1,SERCA}$	K_d for proton binding to SERCA in myoplasm	mM	0.0000109 [6]
$K_{dNaK,atp}$	dissociation constant (NaK)	mM	2.9263 [3]
$K_{dNaK,ke}$	dissociation constant (NaK)	mM	6.80234 [3]
$K_{dNaK,ki}$	dissociation constant (NaK)	mM	255.13 [3]
$K_{dNaK,kpi}$	inorganic phosphate binding constant (NaK)	mM	292.0 [3]
$K_{dNaK,nae1}$	voltage partitioning of dissociation of 'first' Na^+ ion	mM	N/A
$K_{dNaK,nai1}$	voltage partitioning of dissociation of 'first' Na^+ ion	mM	N/A
$K_{dNaK,nae2}$	voltage-independent dissoc. constant for 'other two' Na^+ ions	mM	356.01 [3]
$K_{dNaK,nai2}$	voltage-independent dissoc. constant for 'other two' Na^+ ions	mM	154.39 [3]
$K_{dNaK,napi}$	inorganic phosphate binding constant (NaK)	mM	224.0 [3]
$K^0_{dNaK,nae}$	dissoc. constant of 'first' Na^+ ion at 0mV	mM	141.27 [3]
$K^0_{dNaK,nai}$	dissoc. constant of 'first' Na^+ ion at 0mV	mM	$0.12x10^{-7}$ [3]
$[\widetilde{K}_e]$	apparent concentration of enzyme-bound ion (NaK)	N/A	N/A
k^{+}_{hyd}	forward reaction rate for CO_2 hyrdolysis	$mM^{-1}s^{-1}$	0.000365 [1]
k^{-}_{hyd}	reverse reaction rate for CO_2 hyrdolysis	$mM^{-1}s^{-1}$	0.481 [1]
$[K^+_i]$	intracellular K^+ concentration	mM	N/A
$[\widetilde{K}_i]$	apparent concentration of enzyme-bound ion (NaK)	N/A	N/A
kin	I_{K1} inactivation	N/A	N/A
k_{leak}	rate constant of Ca^{2+} leakage from NSR	ms^{-1}	N/A
$k_{m,Ca}$	half-saturation concentration ($I_{Ca(L)}$)	mM	0.006 [1]
$k_{m,CMDN}$	equilibrium constant of buffering for calmodulin	mM	0.00238 [1]
$k_{m,CSQN}$	equilibrium constant of buffering for calsequestrin	mM	0.8 [1]
$k_{mp(Ca)}$	half-saturation concentration of sarcolemmal Ca^{2+} pump	mM	0.0005 [1]
$k_{m,TRPN}$	equilibrium constant of buffering for troponin	mM	0.0005 [1]
$k_{m,TRPNapp}$	apparent binding constant	mM	N/A
$k_{NBC,hco3}$	dissociation constant (NBC)	mM	0.008017 [1]
$K_{NBC,he}$	dissociation constant (NBC)	mM	0.0000653 [1]
$K_{NBC,hi}$	dissociation constant (NBC)	mM	0.000183 [1]
$k_{NBC,na}$	dissociation constant (NBC)	mM	4,866.11 [1]
k^{+}_{NBC1}	forward rate constant (NBC)	ms^{-1}	3,597.7 [1]
k^{-}_{NBC1}	reverse rate constant (NBC)	ms^{-1}	598.4 [1]
k^{+}_{NBC2}	forward rate constant (NBC)	ms^{-1}	5.12 [1]
k^{-}_{NBC2}	reverse rate constant (NBC)	ms^{-1}	0.8516 [1]
$k_{NHE,h}$	dissociation constant (NHE)	mM	0.000165 [1]
K_{NHE}	dissociation constant (NHE)	mM	0.0003438 [1]
$k_{NHE,na}$	dissociation constant (NHE)	mM	33.58 [1]
k^{+}_{NHE1}	forward rate constant (NHE)	ms^{-1}	197.5 [1]
k^{-}_{NHE1}	reverse rate constant (NHE)	ms^{-1}	725.5 [1]
k^{+}_{NHE2}	forward rate constant (NHE)	ms^{-1}	44.0 [1]
k^{-}_{NHE2}	reverse rate constant (NHE)	ms^{-1}	161.63 [1]
$[K^+_o]$	extracellular K^+ concentration	mM	N/A

Abbreviation	Parameter	Units	Constant Value
kp	K^+ "plateau" factor	N/A	N/A
$k^+_{1,SERCA}$	forward rate constant (SERCA)	$mM^{-1}s^{-1}$	25,900 [6]
$k^-_{1,SERCA}$	reverse rate constant (SERCA)	$mM^{-1}s^{-1}$	2 [6]
$k^+_{2,SERCA}$	forward rate constant (SERCA)	s^{-1}	2,540 [6]
$k^-_{2,SERCA}$	reverse rate constant (SERCA)	$mM^{-1}s^{-1}$	67,200 [6]
$k^+_{3,SERCA}$	forward rate constant (SERCA)	s^{-1}	20.5 [6]
$k^-_{3,SERCA}$	reverse rate constant (SERCA)	$mM^{-1}s^{-1}$	149 [6]
Lp	hydraulic conductivity of membrane	$L*N^{-1}*s^{-1}$	$1.21x10^{-10}$ [3]
m	I_{Na} activation gate	N/A	N/A
m_∞	steady state value for m gate	N/A	N/A
$modifier_{Inal}$	modifier to determine $I_{Na,l}$ max.conductance	N/A	0.0007
			(0.00018 ischemia) [4]
$modifier_{NHE}$	factor to simulate NHE inhibition	N/A	N/A
$n_{AE,he}$	Hill coefficient for binding of extracellular protons to AE	N/A	1.44 [1]
$n_{AE,hi}$	Hill coefficient for binding of intracellular protons to AE	N/A	5.11 [1]
$n_{H,NaCa}$	Hill coefficient for binding of protons to NaCa exchanger	N/A	0.75
$n_{H,RYR}$	Hill coefficient for protons inhibiting RYR	N/A	1.8668 [1]
$n_{H,TRPN}$	Hill coefficient for binding of protons to troponin	N/A	1.6524 [1]
$n_{NBC,he}$	Hill coefficient for binding of extracellular protons to NBC	N/A	2.18 [1]
$n_{NBC,hi}$	Hill coefficient for binding of intracellular protons to NBC	N/A	2.91 [1]
$n_{NHE,he}$	Hill coefficient for binding of extracellular protons to NHE	N/A	1.00 [7]
$n_{NHE,hi}$	Hill coefficient for binding of intracellular protons to NHE	N/A	3.18 [1]
n_{SERCA}	number of H^+ transported via SERCA for every 2 Ca^{2+}	N/A	2.0 [6]
$[\widetilde{Na}_{e,1}]$	apparent concentration of enzyme-bound ion (NaK)	N/A	N/A
$[\widetilde{Na}_{e,2}]$	apparent concentration of enzyme-bound ion (NaK)	N/A	N/A
$[Na^+_i]$	intracellular Na^+ concentration	mM	N/A
$[\widetilde{Na}_{i,1}]$	apparent concentration of enzyme-bound ion (NaK)	N/A	N/A
$[\widetilde{Na}_{i,2}]$	apparent concentration of enzyme-bound ion (NaK)	N/A	N/A
$[Na^+_o]$	extracellular Na^+ concentration	mM	N/A
[NSR]	Ca^{2+} concentration in NSR	mM	N/A
$[\overline{NSR}]$	max. $[Ca^{2+}]$ in NSR	mM	15.0 [1]
$[OH^-_e]$	extracellular hydroxide concentration	mM	N/A
$[OH^-_i]$	intracellular hydroxide concentration	mM	N/A
p	$I_{Na,l}$ activation gate	N/A	N/A
p_∞	steady state value for p gate	N/A	N/A
p_{atm}	atmospheric pressure	mmHg	760
p_{Ca}	permeability of membrane to Ca^{2+}	cm/s	0.00054 [1]
p_{Cl}	membrane permeability to Cl^- ions	cm/ms	0.0000001 [1]
p_K	permeability of membrane to K^+	cm/s	0.000000193 [1]
p_{Na}	permeability of membrane to Na^+	cm/s	0.000000675 [1]
[PCr]	phosphocreatine concentration	mM	N/A
pH_e	extracellular pH	N/A	N/A
pH_i	intracellular pH	N/A	N/A
pH_{ref}	reference pH value ("normal" pH)	N/A	7.15
$[Pi_{free}]$	concentration of free inorganic phosphate	mM	N/A
$[Pi_{NaK}]$	concentration of free inorganic phosphate bound to NaK	mM	N/A
$[Pi_{total}]$	concentration of total phosphate	mM	35.312184 [3]
$pK_{dNaK,hpi}$	pK for inorganic phosphate binding to NaK	N/A	6.77 [3]
$pK_{H,NaCa}$	pK for protons inhibiting NaCa exchanger	N/A	7.00
$pK_{H,RYR}$	pK for protons inhibiting RYR	N/A	6.6396 [1]

Abbreviation	Parameter	Units	Constant Value
$pK_{H,TRPN}$	pK for protons binding to troponin	N/A	6.7914 [1]
pK1	pK for proton binding to first generic intrinsic buffer	N/A	6.03 [1]
pK2	pK for proton binding to second generic intrinsic buffer	N/A	7.57 [1]
$ppCO_2$	carbon dioxide partial pressure	$mmHg^{-1}$	0.03 [1]
prnak	Na^+/K^+ permeability ratio	N/A	0.01833 [1]
$\prod_e$	extracellular osmolarity	mOsm	310 [3]
$\prod_i$	intracellular osmolarity	mOsm	N/A
$[Proton_{ref}]$	concentration of free intracellular protons at simulation start	mM	N/A
R	universal gas constant	J/(K*kmol)	8314.0 [1]
$rate^+_{NaK1}$	baseline rate constant (NaK)	s^{-1}	1664.12 [3]
$rate^-_{NaK1}$	baseline rate constant (NaK)	$s^{-1}mM^{-1}$	264.15 [3]
$rate^+_{NaK2}$	baseline rate constant (NaK)	s^{-1}	110.42 [3]
$rate^-_{NaK2}$	baseline rate constant (NaK)	s^{-1}	14.04 [3]
$rate^+_{NaK3}$	baseline rate constant (NaK)	s^{-1}	2314.26 [3]
$rate^-_{NaK3}$	baseline rate constant (NaK)	$s^{-1}mM^{-2}$	7599999.5 [3]
$rate^+_{NaK4}$	baseline rate constant (NaK)	s^{-1}	462.38 [3]
$rate^-_{NaK4}$	baseline rate constant (NaK)	s^{-1}	1702.53 [3]
rkinac	time-independent I_{Kr} inactivation gate	N/A	N/A
reg_{AE}	modification of AE flux by extra- and intracellular protons	N/A	N/A
reg_{NBC}	modification of NBC flux by extra- and intracellular protons	N/A	N/A
reg_{NHE}	modification of NHE flux by intracellular protons	N/A	N/A
$s1_{AE}$	state occupancy probability (AE)	N/A	N/A
$s1_{CHE}$	state occupancy probability (CHE)	N/A	N/A
$s1_{NBC}$	state occupancy probability (NBC)	N/A	N/A
$s1_{NHE}$	state occupancy probability (NHE)	N/A	N/A
$s6_{AE}$	state occupancy probability (AE)	N/A	N/A
$s6_{CHE}$	state occupancy probability (CHE)	N/A	N/A
$s6_{NBC}$	state occupancy probability (NBC)	N/A	N/A
$s6_{NHE}$	state occupancy probability (NHE)	N/A	N/A
t	ischemic time	min.	N/A
t_{cicr}	Ca^{2+}-induced Ca^{2+} release timer	ms	N/A
t_{jsrol}	counter for Ca^{2+} release from JSR due to overload	ms	N/A
τ_b	time constant for b gate	ms	N/A
τ_d	time constant for d gate	ms	N/A
τ_f	time constant for f gate	ms	N/A
τ_g	time constant for g gate	ms	N/A
τ_h	time constant for h gate	ms	N/A
τ_j	time constant for j gate	ms	N/A
τ_m	time constant for m gate	ms	N/A
τ_{off}	time constant of deactivation of Ca^{2+} release from JSR	ms	0.5 [1]
τ_{on}	time constant of activation of Ca^{2+} release from JSR	ms	0.5 [1]
τ_p	time constant for p gate	ms	N/A
τ_{tr}	time constant of Ca^{2+} transfer from NSR to JSR	ms	180.0 [1]
τ_{xr}	time constant for xr gate	ms	N/A
τ_{xs1}	time constant for xs1 gate	ms	N/A
τ_{xs2}	time constant for xs2 gate	ms	N/A
temp	temperature	K	310.0 [1]
[TRPN]	concentration of Ca^{2+} bound to troponin	mM	N/A
$[\overline{TRPN}]$	max. $[Ca^{2+}]$ bound to troponin	mM	0.070 [1]
V	membrane voltage	mV	N/A
V_{AE}	flux through anion exchanger	$mM*uL*ms^{-1}$	N/A

Abbreviation	Parameter	Units	Constant Value
V_{CHE}	flux through chloride-hydroxide exchanger	$mM*uL*ms^{-1}$	N/A
V_{CO_2}	carbon dioxide flux across membrane	$mM*uL*ms^{-1}$	N/A
$V_{hyd,e}$	reaction rate for extracellular CO_2 hydrolysis	$mM*uL*ms^{-1}$	N/A
$V_{hyd,i}$	reaction rate for intracellular CO_2 hydrolysis	$mM*uL*ms^{-1}$	N/A
V_{H_2O}	movement of water across membrane	$uL*cm^{-2}*ms^{-1}$	N/A
V_{NBC}	flux through sodium-bicarbonate symporter	$mM*uL*ms^{-1}$	N/A
V_{NHE}	flux through sodium-hydrogen exchanger	$mM*uL*ms^{-1}$	N/A
V_{SERCA}	SERCA flux	mM/ms	N/A
Vol_{cell}	total cell volume	uL	N/A
$Vol_{external}$	extracellular compartment volume	uL	N/A
Vol_{jsr}	JSR volume	uL	N/A
Vol_{myo}	myoplasm volume	uL	N/A
Vol_{nsr}	NSR volume	uL	N/A
Vol_{sr}	SR volume	uL	N/A
Vol_{total}	total cell volume	uL	$43.182x10^{-6}$ [3]
xr	I_{Kr} inactivation gate	N/A	N/A
xr_∞	steady state value for xr gate	N/A	N/A
xs1	I_{Ks} inactivation gate	N/A	N/A
$xs1_\infty$	steady state value for xs1 gate	N/A	N/A
xs2	I_{Ks} inactivation gate	N/A	N/A
$xs2_\infty$	steady state value for xs2 gate	N/A	N/A
$[X_e^{zi-}]$	concentration of extracellular impermeable osmolytes	nM	N/A
$[X_i^{zi-}]$	concentration of intracellular impermeable osmolytes	nM	N/A
z_{Ca}	Ca^{2+} valence	N/A	2.0
z_{Cl}	Cl^- valence	N/A	-1.0
z_K	K^+ valence	N/A	1.0
z_{Na}	Na^+ valence	N/A	1.0

Model Equations

$\mathbf{I_{Na}}$

$$\alpha_{\mathrm{m}} = 0.32 * \frac{\mathrm{V} + 47.13}{1 - \exp(-0.1 * (\mathrm{V} + 47.13))} \tag{S.1}$$

$$\beta_{\mathrm{m}} = 0.08 * \exp\left(\frac{-\mathrm{V}}{11.0}\right) \tag{S.2}$$

$$\tau_{\mathrm{m}} = \frac{1}{\alpha_{\mathrm{m}} + \beta_{\mathrm{m}}} \tag{S.3}$$

$$\mathrm{m}_{\infty} = \alpha_{\mathrm{m}} * \tau_{\mathrm{m}} \tag{S.4}$$

$$\mathrm{m}_{\mathrm{t}} = \mathrm{m}_{\infty} - (\mathrm{m}_{\infty} - \mathrm{m}_{\text{t-}\Delta\mathrm{t}}) * \exp\left(\frac{-\Delta\mathrm{t}}{\tau_{\mathrm{m}}}\right) \tag{S.5}$$

$$\alpha_{\mathrm{h}} = 0.135 * \exp\left(\frac{-\mathrm{V} - 80.0}{6.8}\right) \tag{S.6}$$

$$\beta_{\mathrm{h}} = \frac{7.5}{1 + \exp(-0.1 * (\mathrm{V} + 11.0))} \tag{S.7}$$

$$\tau_{\mathrm{h}} = \frac{1}{\alpha_{\mathrm{h}} + \beta_{\mathrm{h}}} \tag{S.8}$$

$$\mathrm{h}_{\infty} = \alpha_{\mathrm{h}} * \tau_{\mathrm{h}} \tag{S.9}$$

$$\mathrm{h}_{\mathrm{t}} = \mathrm{h}_{\infty} - (\mathrm{h}_{\infty} - \mathrm{h}_{\text{t-}\Delta\mathrm{t}}) * \exp\left(\frac{-\Delta\mathrm{t}}{\tau_{\mathrm{h}}}\right) \tag{S.10}$$

$$\alpha_{\mathrm{j}} = \frac{0.175 * \exp\left(\frac{-\mathrm{V} - 100.0}{23.0}\right)}{1.0 + \exp(0.15 * (\mathrm{V} + 79.0))} \tag{S.11}$$

$$\beta_{\mathrm{j}} = \frac{0.3}{1 + \exp(-0.1 * (\mathrm{V} + 32.0))} \tag{S.12}$$

$$\tau_{\mathrm{j}} = \frac{1}{\alpha_{\mathrm{j}} + \beta_{\mathrm{j}}} \tag{S.13}$$

$$\mathrm{j}_{\infty} = \alpha_{\mathrm{j}} * \tau_{\mathrm{j}} \tag{S.14}$$

$$\mathrm{j}_{\mathrm{t}} = \mathrm{j}_{\infty} - (\mathrm{j}_{\infty} - \mathrm{j}_{\text{t-}\Delta\mathrm{t}}) * \exp\left(\frac{-\Delta\mathrm{t}}{\tau_{\mathrm{j}}}\right) \tag{S.15}$$

$$\mathrm{I}_{\mathrm{Na}} = \mathrm{g}_{\mathrm{Na}} * \mathrm{m}^3 * \mathrm{h} * \mathrm{j} * (\mathrm{V} - \mathrm{E}_{\mathrm{Na}}) \tag{S.16}$$

$I_{Na,b}$

$$I_{Na,b} = g_{Na,b} * (V - E_{Na}) \tag{S.17}$$

$I_{Na,l}$

$$\alpha_p = 19.0 * \exp\left(\frac{V}{16.5}\right) \tag{S.18}$$

$$\beta_p = 0.2 * \exp\left(\frac{-V}{20.0}\right) \tag{S.19}$$

$$\tau_p = \frac{1}{\alpha_p + \beta_p} \tag{S.20}$$

$$p_\infty = \alpha_p * \tau_p \tag{S.21}$$

$$p_t = p_\infty - (p_\infty - p_{t\text{-}\Delta t}) * \exp\left(\frac{-\Delta t}{\tau_p}\right) \tag{S.22}$$

$$I_{Na,l} = \text{modifier}_{Inal} * g_{Na} * p^3 * (V - E_{Na}) \tag{S.23}$$

$I_{Ca(L)}$

$$d_\infty = \frac{1}{1 + \exp\left(\frac{-V - 10}{6.24}\right)} \tag{S.24}$$

$$\tau_d = d_\infty * \frac{1 - \exp\left(\frac{-V - 10}{6.24}\right)}{0.035 * (V + 10)} \tag{S.25}$$

$$d_t = d_\infty - (d_\infty - d_{t\text{-}\Delta t}) * \exp\left(\frac{-\Delta t}{\tau_d}\right) \tag{S.26}$$

$$f_\infty = \frac{1}{1 + \exp\left(\frac{V + 32}{8}\right)} + \frac{0.6}{1 + \exp\left(\frac{50 - V}{20}\right)} \tag{S.27}$$

$$\tau_f = \frac{1}{(0.0197 * \exp(-((0.0337 * (V + 10)) * (0.0337 * (V + 10)))) + 0.02)} \tag{S.28}$$

$$f_t = f_\infty - (f_\infty - f_{t\text{-}\Delta t}) * \exp\left(\frac{-\Delta t}{\tau_f}\right) \tag{S.29}$$

$$f_{Ca,ATP} = \frac{1}{1 + \left(\frac{k_{ATP}}{[ATP]}\right)^{2.6}} \tag{S.30}$$

$$f_{Ca} = \frac{1}{1 + \frac{[Ca_i^{2+}]}{k_{m,Ca}}} \tag{S.31}$$

$$\bar{I}_{Ca} = p_{Ca} * z_{Ca} * z_{Ca} * \frac{V * F * F}{R * temp} * \frac{gacai * [Ca_i^{2+}] * \exp\left(\frac{z_{Ca} * V * F}{R * temp}\right) - gacao * [Ca_o^{2+}]}{\exp\left(\frac{z_{Ca} * V * F}{R * temp}\right) - 1} \tag{S.32}$$

$$\bar{I}_{Na} = p_{Na} * z_{Na} * z_{Na} * \frac{V * F * F}{R * temp} * \frac{ganai * [Na_i^{+}] * \exp\left(\frac{z_{Na} * V * F}{R * temp}\right) - ganao * [Na_o^{+}]}{\exp\left(\frac{z_{Na} * V * F}{R * temp}\right) - 1} \tag{S.33}$$

$$\bar{I}_{K} = p_{K} * z_{K} * z_{K} * \frac{V * F * F}{R * temp} * \frac{gaki * [K_i^{+}] * \exp\left(\frac{z_{K} * V * F}{R * temp}\right) - gako * [K_o^{+}]}{\exp\left(\frac{z_{K} * V * F}{R * temp}\right) - 1} \tag{S.34}$$

$$I_{Ca(L)} = f_{Ca,ATP} * d * f * f_{Ca} * \bar{I}_{Ca} \tag{S.35}$$

$$I_{Ca(L),Na} = f_{Ca,ATP} * d * f * f_{Ca} * \bar{I}_{Na} \tag{S.36}$$

$$I_{Ca(L),K} = f_{Ca,ATP} * d * f * f_{Ca} * \bar{I}_{K} \tag{S.37}$$

$\mathbf{I_{Ca,t}}$

$$b_\infty = \frac{1}{1 + \exp\left(\frac{-V - 14}{10.8}\right)} \tag{S.38}$$

$$\tau_b = 3.7 + \frac{6.1}{1 + \exp\left(\frac{V + 25.0}{4.5}\right)} \tag{S.39}$$

$$\mathrm{b_t = b_\infty - (b_\infty - b_{t\text{-}\Delta t}) * \exp\left(\frac{-\Delta t}{\tau_b}\right)} \tag{S.40}$$

$$\mathrm{g_\infty = \frac{1}{1 + \exp\left(\frac{V + 60.0}{5.6}\right)}} \tag{S.41}$$

If V $\leq$ 0.0:

$$\mathrm{\tau_g = -0.875 * V + 12.0} \tag{S.42}$$

Otherwise:

$$\mathrm{\tau_g = 12.0} \tag{S.43}$$

$$\mathrm{g_t = g_\infty - (g_\infty - g_{t\text{-}\Delta t}) * \exp\left(\frac{-\Delta t}{\tau_g}\right)} \tag{S.44}$$

$$\mathrm{I_{Ca,t} = g_{Ca,t} * b^2 * g * (V - E_{Ca})} \tag{S.45}$$

$\mathbf{I_{p(Ca)}}$

$$\mathrm{I_{p(Ca)} = \frac{\bar{I}_{p(Ca)} * Ca_i^{2+}}{k_{mp(Ca)} + Ca_i^{2+}}} \tag{S.46}$$

$\mathbf{I_{Ca,b}}$

$$\mathrm{I_{Ca,b} = g_{Ca,b} * (V - E_{Ca})} \tag{S.47}$$

JSR

$$\mathrm{I_{tr} = \frac{[nsr] - [jsr]}{\tau_{tr}}} \tag{S.48}$$

$$\mathrm{dI_{Ca,total} = \frac{I_{Ca,total\ t} - I_{Ca,total\ t\text{-}\Delta t}}{\Delta t}} \tag{S.49}$$

$$\mathrm{jsr_{on} = \frac{1}{1 + \exp\left(\frac{-t_{cicr} + 4.0}{\tau_{on}}\right)}} \tag{S.50}$$

$$\mathrm{jsr_{off} = 1 - \frac{1}{1 + \exp\left(\frac{-t_{cicr} + 4.0}{\tau_{off}}\right)}} \tag{S.51}$$

$$\mathrm{jsr_{magrel}} = \frac{1}{1 + \exp\left(\frac{\mathrm{I_{Ca,total}} + 5.0}{0.9}\right)} \tag{S.52}$$

$$\mathrm{fac_{H,RYR}} = \frac{\left(\frac{1 + 10^{\mathrm{n_{H,RYR}}(\text{-}7.4763+\mathrm{pK_{H,RYR}})}}{1 + 10^{\mathrm{n_{H,RYR}}(\text{-}\mathrm{pH_i}+\mathrm{pK_{H,RYR}})}}\right)}{\left(\frac{1 + 10^{\mathrm{n_{H,RYR}}(\text{-}7.4763+\mathrm{pK_{H,RYR}})}}{1 + 10^{\mathrm{n_{H,RYR}}(\text{-}\mathrm{pH_{ref}}+\mathrm{pK_{H,RYR}})}}\right)} \tag{S.53}$$

$$\mathrm{I_{relcicr}} = \mathrm{fac_{H,RYR}} * \mathrm{g_{maxrel}} * \mathrm{jsr_{on}} * \mathrm{jsr_{off}} * \mathrm{jsr_{magrel}} * ([\mathrm{JSR}] - [\mathrm{Ca_i^{2+}}]) \tag{S.54}$$

$$\mathrm{g_{reljsrol}} = \mathrm{g_{relbarjsrol}} * \left(1 - \exp\left(\frac{-\mathrm{t_{jsrol}}}{\mathrm{tau_{on}}}\right)\right) * \exp\left(\frac{-\mathrm{t_{\text{-}tjsrol}}}{\mathrm{tau_{off}}}\right) \tag{S.55}$$

$$\mathrm{I_{reljsrol}} = \mathrm{g_{reljsrol}} * ([\mathrm{JSR}] - [\mathrm{Ca_i^{2+}}]) \tag{S.56}$$

$$\frac{\mathrm{d[JSR]}}{\mathrm{dt}} = \frac{\mathrm{I_{tr}} - \mathrm{I_{relcicr}} - \mathrm{I_{reljsrol}}}{\beta_{\mathrm{CSQN}}} \tag{S.57}$$

NSR

$$\mathrm{k_{leak}} = \frac{\bar{\mathrm{I}}_{\mathrm{up}}}{\overline{[\mathrm{NSR}]}} \tag{S.58}$$

$$\mathrm{I_{leak}} = \mathrm{k_{leak}} * [\mathrm{NSR}] \tag{S.59}$$

$$\frac{\mathrm{d[NSR]}}{\mathrm{dt}} = \mathrm{V_{SERCA}} - \mathrm{I_{leak}} - \mathrm{I_{tr}}\left(\frac{\mathrm{Vol_{jsr}}}{\mathrm{Vol_{nsr}}}\right) \tag{S.60}$$

SERCA

$$\widetilde{\mathrm{Ca}}_{\mathrm{i,SERCA}} = \frac{[\mathrm{Ca_i^{2+}}]}{\mathrm{K_{d,Cai,SERCA}}} \tag{S.61}$$

$$\widetilde{\mathrm{Ca}}_{\mathrm{SR,SERCA}} = \frac{[\mathrm{NSR}]}{\mathrm{K_{d,CaSR,SERCA}}} \tag{S.62}$$

$$\widetilde{\mathrm{H}}_{\mathrm{i,SERCA}} = \frac{[\mathrm{H_i^+}]^{\mathrm{n_{SERCA}}}}{\mathrm{K_{d,Hi,SERCA}}} \tag{S.63}$$

$$\widetilde{\mathrm{H}}_{\mathrm{SR,SERCA}} = \frac{[\mathrm{H_i^+}]^{\mathrm{n_{SERCA}}}}{\mathrm{K_{d,HSR,SERCA}}} \tag{S.64}$$

$$\widetilde{\mathrm{H}}_{\mathrm{1,SERCA}} = \frac{[\mathrm{H}]^+}{\mathrm{K_{d,H1,SERCA}}} \tag{S.65}$$

$$\widetilde{\mathrm{H}}_{\mathrm{SERCA}} = \frac{[\mathrm{H}]^+}{\mathrm{K_{d,H,SERCA}}} \tag{S.66}$$

$$\alpha^+_{1,\mathrm{SERCA}} = \mathrm{k}^+_{1,\mathrm{SERCA}}[\mathrm{ATP}] \tag{S.67}$$

$$\alpha^+_{2,\mathrm{SERCA}} = \frac{\mathrm{k}^+_{2,\mathrm{SERCA}}\widetilde{\mathrm{Ca}}^2_{\mathrm{i,SERCA}}}{\widetilde{\mathrm{Ca}}^2_{\mathrm{i,SERCA}}(1+\widetilde{\mathrm{H}}_{\mathrm{i,SERCA}}) + \widetilde{\mathrm{H}}_{\mathrm{i,SERCA}}(1+\widetilde{\mathrm{H}}_{\mathrm{1,SERCA}})} \tag{S.68}$$

$$\alpha^+_{3,\mathrm{SERCA}} = \frac{\mathrm{k}^+_{3,\mathrm{SERCA}}\widetilde{\mathrm{H}}_{\mathrm{SR,SERCA}}}{\widetilde{\mathrm{H}}_{\mathrm{SERCA}}(1+\widetilde{\mathrm{Ca}}^2_{\mathrm{SR,SERCA}}) + \widetilde{\mathrm{H}}_{\mathrm{SR,SERCA}}(1+\widetilde{\mathrm{H}}_{\mathrm{SERCA}})} \tag{S.69}$$

$$\alpha^-_{1,\mathrm{SERCA}} = \frac{\mathrm{k}^-_1\widetilde{\mathrm{H}}_{\mathrm{i,SERCA}}}{\widetilde{\mathrm{Ca}}^2_{\mathrm{i,SERCA}}(1+\widetilde{\mathrm{H}}_{\mathrm{i,SERCA}}) + \widetilde{\mathrm{H}}_{\mathrm{i,SERCA}}(1+\widetilde{\mathrm{H}}_{\mathrm{1,SERCA}})} \tag{S.70}$$

$$\alpha^-_{2,\mathrm{SERCA}} = \frac{\mathrm{k}^-_{2,\mathrm{SERCA}}[\mathrm{ADP}]\widetilde{\mathrm{Ca}}^2_{\mathrm{SR,SERCA}}\widetilde{\mathrm{H}}_{\mathrm{SR,SERCA}}}{\widetilde{\mathrm{H}}_{\mathrm{SERCA}}(1+\widetilde{\mathrm{Ca}}^2_{\mathrm{SR,SERCA}}) + \widetilde{\mathrm{H}}_{\mathrm{SR,SERCA}}(1+\widetilde{\mathrm{H}}_{\mathrm{SERCA}})} \tag{S.71}$$

$$\alpha^-_{3,\mathrm{SERCA}} = \mathrm{k}^-_{3,\mathrm{SERCA}}[\mathrm{Pi_{free}}] \tag{S.72}$$

$$\begin{aligned}\Sigma_3 = {} & \alpha^+_{2,\mathrm{SERCA}}\alpha^+_{3,\mathrm{SERCA}} + \alpha^-_{1,\mathrm{SERCA}}\alpha^+_{3,\mathrm{SERCA}} + \alpha^-_{1,\mathrm{SERCA}}\alpha^-_{2,\mathrm{SERCA}} + \\ & \alpha^+_{1,\mathrm{SERCA}}\alpha^+_{3,\mathrm{SERCA}} + \alpha^-_{2,\mathrm{SERCA}}\alpha^+_{1,\mathrm{SERCA}} + \alpha^-_{2,\mathrm{SERCA}}\alpha^-_{3,\mathrm{SERCA}} + \\ & \alpha^+_{1,\mathrm{SERCA}}\alpha^+_{2,\mathrm{SERCA}} + \alpha^-_{3,\mathrm{SERCA}}\alpha^-_{1,\mathrm{SERCA}} + \alpha^-_{3,\mathrm{SERCA}}\alpha^+_{2,\mathrm{SERCA}}\end{aligned} \tag{S.73}$$

$$\mathrm{cyc_{SERCA}} = \frac{\alpha^+_{1,\mathrm{SERCA}}\alpha^+_{2,\mathrm{SERCA}}\alpha^+_{3,\mathrm{SERCA}} - \alpha^-_{1,\mathrm{SERCA}}\alpha^-_{2,\mathrm{SERCA}}\alpha^-_{3,\mathrm{SERCA}}}{\Sigma_3} \tag{S.74}$$

$$\mathrm{V_{SERCA}} = 0.00820 * \mathrm{cyc_{SERCA}} \tag{S.75}$$

$\mathbf{Ca^{2+}}$ Buffering

$$k_{m,TRPNapp} = k_{m,TRPN}\left(\frac{1 + 10^{n_{H,TRPN}(\text{-}pH_i + pK_{H,TRPN})}}{1 + 10^{n_{H,TRPN}(\text{-}pH_{ref} + pK_{H,TRPN})}}\right) \tag{S.76}$$

$$\beta_{TRPN} = 1 + \frac{[\overline{TRPN}] * k_{m,TRPNapp}}{([Ca_i^{2+}] + k_{m,TRPNapp})^2} \tag{S.77}$$

$$[TRPN] = [\overline{TRPN}]\left(\frac{[Ca_i^{2+}]}{[Ca_i^{2+}] + k_{m,TRPNapp}}\right) \tag{S.78}$$

$$\beta_{CSQN} = 1 + \frac{[\overline{CSQN}] * k_{m,CSQN}}{([JSR] + k_{m,CSQN})^2} \tag{S.79}$$

$$[CSQN] = [\overline{CSQN}]\left(\frac{[JSR]}{[JSR] + k_{m,CSQN}}\right) \tag{S.80}$$

$$\beta_{CMDN} = 1 + \frac{[\overline{CMDN}] * k_{m,CMDN}}{([Ca_i^{2+}] + k_{m,CMDN})^2} \tag{S.81}$$

$$[CMDN] = [\overline{CMDN}]\left(\frac{[Ca_i^{2+}]}{[Ca_i^{2+}] + k_{m,CMDN}}\right) \tag{S.82}$$

$\mathbf{I_{Kr}}$

$$rkinac = \frac{1}{1 + \exp\left(\frac{V + 9}{22.4}\right)} \tag{S.83}$$

$$g_{Kr} = 0.02614\sqrt{\frac{[K_o^+]}{5.4}} \tag{S.84}$$

$$xr_\infty = \frac{1}{1 + \exp\left(\frac{-V - 21.5}{7.5}\right)} \tag{S.85}$$

$$\tau_{xr} = \frac{1}{\frac{0.00138 * (V + 14.2)}{(1 - \exp(-0.123 * (V + 14.2)))} + \frac{0.00061 * (V + 38.9)}{(\exp(0.145 * (V + 38.9)) - 1)}} \tag{S.86}$$

$$xr_t = xr_\infty - (xr_\infty - xr_{t\text{-}\Delta t}) * \exp\left(\frac{-\Delta t}{\tau_{xr}}\right) \tag{S.87}$$

$$I_{Kr} = g_{Kr} * xr * rkinac * (V - E_K) \tag{S.88}$$

$\mathbf{I_{Ks}}$

$$\mathrm{g_{Ks}} = 0.433\left(1 + \left(\frac{0.6}{1 + \left(\frac{0.000038}{[\mathrm{Ca_i^{2+}}]}\right)^{1.4}}\right)\right) \tag{S.89}$$

$$\mathrm{xs1_{\infty}} = \frac{1}{1 + \exp\left(\frac{-\mathrm{V} + 1.5}{16.7}\right)} \tag{S.90}$$

$$\tau_{\mathrm{xs1}} = \frac{1}{\frac{0.0000719 * (\mathrm{V} + 30)}{(1 - \exp(-0.148 * (\mathrm{V} + 30)))} + \frac{0.000131 * (\mathrm{V} + 30)}{(\exp(0.0687 * (\mathrm{V} + 30)) - 1)}} \tag{S.91}$$

$$\mathrm{xs1_t} = \mathrm{xs1_{\infty}} - (\mathrm{xs1_{\infty}} - \mathrm{xs1_{t\text{-}\Delta t}}) * \exp\left(\frac{-\Delta \mathrm{t}}{\tau_{\mathrm{xs1}}}\right) \tag{S.92}$$

$$\mathrm{xs2_{\infty}} = \frac{1}{1 + \exp\left(\frac{-\mathrm{V} + 1.5}{16.7}\right)} \tag{S.93}$$

$$\tau_{\mathrm{xs2}} = \frac{4}{\frac{0.0000719 * (\mathrm{V} + 30)}{(1 - \exp(-0.148 * (\mathrm{V} + 30)))} + \frac{0.000131 * (\mathrm{V} + 30)}{(\exp(0.0687 * (\mathrm{V} + 30)) - 1)}} \tag{S.94}$$

$$\mathrm{xs2_t} = \mathrm{xs2_{\infty}} - (\mathrm{xs2_{\infty}} - \mathrm{xs2_{t\text{-}\Delta t}}) * \exp\left(\frac{-\Delta \mathrm{t}}{\tau_{\mathrm{xs2}}}\right) \tag{S.95}$$

$$\mathrm{I_{Ks}} = \mathrm{g_{Ks}} * \mathrm{xs1} * \mathrm{xs2} * (\mathrm{V} - \mathrm{E_{Ks}}) \tag{S.96}$$

$\mathbf{I_{K1}}$

$$\alpha_{\mathrm{K1}} = \frac{1.02}{1 + \exp(0.2385(\mathrm{V} - \mathrm{E_K} - 59.215))} \tag{S.97}$$

$$\beta_{\mathrm{K1}} = \frac{0.49124 * \exp(0.08032 * (\mathrm{V} - \mathrm{E_K} + 5.476)) + \exp(0.06175 * (\mathrm{V} - \mathrm{E_K} - 594.31))}{1 + \exp(-0.5143 * (\mathrm{V} - \mathrm{E_K} + 4.753))} \tag{S.98}$$

$$\mathrm{kin} = \frac{\alpha_{\mathrm{K1}}}{\alpha_{\mathrm{K1}} + \beta_{\mathrm{K1}}} \tag{S.99}$$

$$\mathrm{g_{K1}} = 0.75\sqrt{\frac{[\mathrm{K_o^+}]}{5.4}} \tag{S.100}$$

$$\mathrm{I_{K1}} = \mathrm{g_{K1}} * \mathrm{kin} * (\mathrm{V} - \mathrm{E_K}) \tag{S.101}$$

$\mathbf{I_{Kp}}$

$$\mathrm{kp} = \frac{1}{1 + \exp\left(\dfrac{7.488 - \mathrm{V}}{5.98}\right)} \tag{S.102}$$

$$\mathrm{I_{Kp}} = \mathrm{g_{Kp}} * \mathrm{kp} * (\mathrm{V} - \mathrm{E_K}) \tag{S.103}$$

$\mathbf{I_{K(ATP)}}$

$$\mathrm{f_{ATP}} = \left(1 - \frac{[\mathrm{ATP}]}{[\mathrm{ATP}] + 12.0}\right)^4 \tag{S.104}$$

$$\mathrm{f^{'}_{ADP}} = \left(\frac{[\mathrm{ADP}]}{[\mathrm{ADP}] + 421.05}\right)^2 \tag{S.105}$$

$$\mathrm{c_{ADP}} = 1 - \mathrm{f^{'}_{ADP}} \tag{S.106}$$

$$\mathrm{f_{ADP}} = 1 - \mathrm{c^4_{ADP}} \tag{S.107}$$

$$\mathrm{f_{K(ATP)}} = 0.08 * \mathrm{f_{ATP}} * (1 - \mathrm{f_{ADP}}) + 0.89 * \mathrm{f_{ATP}} * \mathrm{f_{ADP}} \tag{S.108}$$

$$\mathrm{g_{K(ATP)}} = 0.4 * \mathrm{f_{K(ATP)}} * \left(\frac{[\mathrm{K^+_o}]}{4.5}\right)^{0.24} \tag{S.109}$$

$$\mathrm{I_{K(ATP)}} = \mathrm{g_{K(ATP)}} * (\mathrm{V} - \mathrm{E_K}) \tag{S.110}$$

Na-Ca Exchange

$$\mathrm{fac_{NaCa}} = \frac{1 + 10^{\mathrm{n_{H,NaCa}(\text{-}pH_{ref} + pK_{H,NaCa})}}}{1 + 10^{\mathrm{n_{H,NaCa}(\text{-}pH_i + pK_{H,NaCa})}}} \tag{S.111}$$

$$\mathrm{I_{NaCa}} = \mathrm{c1} * \mathrm{fac_{NaCa}} * \exp\left(\frac{(\mathrm{gammas} - 1) * \mathrm{V} * \mathrm{F}}{\mathrm{R} * \mathrm{temp}}\right) * \left(\frac{\exp\left(\dfrac{\mathrm{V} * \mathrm{F}}{\mathrm{R} * \mathrm{temp}}\right)[\mathrm{Na^+_i}]^3 * [\mathrm{Ca^{2+}_o}] - [\mathrm{Na^+_o}]^3 * [\mathrm{Ca^{2+}_i}]}{1 + \mathrm{c2} * \exp\left(\dfrac{(\mathrm{gammas} - 1) * \mathrm{V} * \mathrm{F}}{\mathrm{R} * \mathrm{temp}}\right) * \exp\left(\dfrac{\mathrm{V} * \mathrm{F}}{\mathrm{R} * \mathrm{temp}}\right) * [\mathrm{Na^+_i}]^3 * [\mathrm{Ca^{2+}_o}] + [\mathrm{Na^+_o}]^3 * [\mathrm{Ca^{2+}_i}]}\right) \tag{S.112}$$

NaK Pump

$$K_{dNaK,nae1} = K^0_{dNaK,nae} * \exp\left(\frac{(1 + factorNaKdel) * F * V}{R * temp}\right) \tag{S.113}$$

$$K_{dNaK,nai1} = K^0_{dNaK,nai} * \exp\left(\frac{(1 + factorNaKdel) * F * V}{R * temp}\right) \tag{S.114}$$

$$[\widetilde{Na}_{e,1}] = \frac{[Na^+_o]}{K_{dNaK,nae1}} \tag{S.115}$$

$$[\widetilde{Na}_{i,1}] = \frac{[Na^+_i]}{K_{dNaK,nai1}} \tag{S.116}$$

$$[\widetilde{Na}_{e,2}] = \frac{[Na^+_o]}{K_{dNaK,nae2}} \tag{S.117}$$

$$[\widetilde{Na}_{i,2}] = \frac{[Na^+_i]}{K_{dNaK,nai2}} \tag{S.118}$$

$$[\widetilde{K}_e] = \frac{[K^+_o]}{K_{dNaK,ke}} \tag{S.119}$$

$$[\widetilde{K}_i] = \frac{[K^+_i]}{K_{dNaK,ki}} \tag{S.120}$$

$$[\widetilde{ATP}] = \frac{[ATP]}{K_{dNaK,atp}} \tag{S.121}$$

$$[Pi_{NaK}] = \left(\frac{[Pi_{free}]}{1 + \frac{[K^+_i]}{K_{dNak,kpi}} + \frac{[H^+_i]}{10^{3\text{-}pK_{dNaK,hpi}}} + \frac{[Na^+_i]}{K_{dNaK,napi}}}\right) \tag{S.122}$$

$$\alpha^+_{NaK1} = rate^+_{NaK1} * [\widetilde{Na}_{i,1}] * \frac{[\widetilde{Na}_{i,2}]^2}{(1 + [\widetilde{Na}_{i,1}]) * (1 + [\widetilde{Na}_{i,2}])^2 + (1 + [\widetilde{K}_i])^2 - 1} \tag{S.123}$$

$$\alpha^+_{NaK2} = rate^+_{NaK2} \tag{S.124}$$

$$\alpha^+_{NaK3} = rate^+_{NaK3} * \frac{[\widetilde{K}_e]^2}{(1 + [\widetilde{Na}_{e,1}]) * (1 + [\widetilde{Na}_{e,2}])^2 + (1 + [\widetilde{K}_e])^2 - 1} \tag{S.125}$$

$$\alpha^{+}_{\mathrm{NaK4}} = \mathrm{rate}^{+}_{\mathrm{NaK4}} * \frac{[\widetilde{\mathrm{ATP}}]}{1 + [\widetilde{\mathrm{ATP}}]} \tag{S.126}$$

$$\alpha^{-}_{\mathrm{NaK1}} = \mathrm{rate}^{-}_{\mathrm{NaK1}} * [\mathrm{ADP}] \tag{S.127}$$

$$\alpha^{-}_{\mathrm{NaK2}} = \mathrm{rate}^{-}_{\mathrm{NaK2}} * \frac{[\widetilde{\mathrm{Na}}_{\mathrm{e,1}}] * [\widetilde{\mathrm{Na}}_{\mathrm{e,2}}]^2}{(1 + [\widetilde{\mathrm{Na}}_{\mathrm{e,1}}]) * (1 + [\widetilde{\mathrm{Na}}_{\mathrm{e,2}}])^2 + (1 + [\widetilde{\mathrm{K}}_{\mathrm{e}}])^2 - 1} \tag{S.128}$$

$$\alpha^{-}_{\mathrm{NaK3}} = \mathrm{rate}^{-}_{\mathrm{NaK3}} * \frac{[\mathrm{Pi}_{\mathrm{NaK}}] * [\mathrm{H}^{+}_{\mathrm{i}}]}{1 + [\widetilde{\mathrm{ATP}}]} \tag{S.129}$$

$$\alpha^{-}_{\mathrm{NaK4}} = \mathrm{rate}^{-}_{\mathrm{NaK4}} * \frac{[\widetilde{\mathrm{K}}_{\mathrm{i}}]^2}{(1 + [\widetilde{\mathrm{Na}}_{\mathrm{i,1}}]) * (1 + [\widetilde{\mathrm{Na}}_{\mathrm{i,2}}])^2 + (1 + [\widetilde{\mathrm{K}}_{\mathrm{i}}])^2 - 1} \tag{S.130}$$

$$\begin{aligned}\Sigma_{\mathrm{NaK}} = {} & \alpha^{-}_{\mathrm{NaK1}}\alpha^{-}_{\mathrm{NaK2}}\alpha^{-}_{\mathrm{NaK3}} + \alpha^{-}_{\mathrm{NaK1}}\alpha^{-}_{\mathrm{NaK2}}\alpha^{+}_{\mathrm{NaK4}} + \alpha^{-}_{\mathrm{NaK1}}\alpha^{+}_{\mathrm{NaK3}}\alpha^{+}_{\mathrm{NaK4}} + \alpha^{+}_{\mathrm{NaK2}}\alpha^{+}_{\mathrm{NaK3}}\alpha^{+}_{\mathrm{NaK4}} + \\ & \alpha^{-}_{\mathrm{NaK2}}\alpha^{-}_{\mathrm{NaK3}}\alpha^{-}_{\mathrm{NaK4}} + \alpha^{+}_{\mathrm{NaK1}}\alpha^{-}_{\mathrm{NaK2}}\alpha^{-}_{\mathrm{NaK3}} + \alpha^{+}_{\mathrm{NaK1}}\alpha^{-}_{\mathrm{NaK2}}\alpha^{+}_{\mathrm{NaK4}} + \alpha^{+}_{\mathrm{NaK1}}\alpha^{+}_{\mathrm{NaK3}}\alpha^{+}_{\mathrm{NaK4}} + \\ & \alpha^{-}_{\mathrm{NaK1}}\alpha^{-}_{\mathrm{NaK3}}\alpha^{-}_{\mathrm{NaK4}} + \alpha^{+}_{\mathrm{NaK2}}\alpha^{-}_{\mathrm{NaK3}}\alpha^{-}_{\mathrm{NaK4}} + \alpha^{+}_{\mathrm{NaK1}}\alpha^{+}_{\mathrm{NaK2}}\alpha^{-}_{\mathrm{NaK3}} + \alpha^{+}_{\mathrm{NaK1}}\alpha^{+}_{\mathrm{NaK2}}\alpha^{+}_{\mathrm{NaK4}} + \\ & \alpha^{-}_{\mathrm{NaK1}}\alpha^{-}_{\mathrm{NaK2}}\alpha^{-}_{\mathrm{NaK4}} + \alpha^{-}_{\mathrm{NaK1}}\alpha^{+}_{\mathrm{NaK3}}\alpha^{-}_{\mathrm{NaK4}} + \alpha^{+}_{\mathrm{NaK2}}\alpha^{+}_{\mathrm{NaK3}}\alpha^{-}_{\mathrm{NaK4}} + \alpha^{+}_{\mathrm{NaK1}}\alpha^{+}_{\mathrm{NaK2}}\alpha^{+}_{\mathrm{NaK3}}\end{aligned} \tag{S.131}$$

$$\mathrm{cyc}_{\mathrm{NaK}} = \frac{\alpha^{+}_{\mathrm{NaK1}}\alpha^{+}_{\mathrm{NaK2}}\alpha^{+}_{\mathrm{NaK3}}\alpha^{+}_{\mathrm{NaK4}} - \alpha^{-}_{\mathrm{NaK1}}\alpha^{-}_{\mathrm{NaK2}}\alpha^{-}_{\mathrm{NaK3}}\alpha^{-}_{\mathrm{NaK4}}}{\Sigma_{\mathrm{NaK}}} \tag{S.132}$$

$$\mathrm{I}_{\mathrm{NaK}} = .000016 * \mathrm{density}_{\mathrm{NaK}} * \mathrm{cyc}_{\mathrm{NaK}} \tag{S.133}$$

$\mathbf{I_{Cl}}$

$$\mathrm{I}_{\mathrm{Cl}} = \mathrm{p}_{\mathrm{Cl}} * \frac{\mathrm{F}^2 * \mathrm{V}}{\mathrm{R} * \mathrm{temp}} * \frac{[\mathrm{Cl}^{-}_{\mathrm{i}}] - [\mathrm{Cl}^{-}_{\mathrm{e}}] * \exp\left(\frac{\mathrm{F} * \mathrm{V}}{\mathrm{R} * \mathrm{temp}}\right)}{1 - \exp\left(\frac{\mathrm{F} * \mathrm{V}}{\mathrm{R} * \mathrm{temp}}\right)} \tag{S.134}$$

Anion Exchanger

$$\mathrm{aa}_{\mathrm{AE}} = 1 + \frac{\mathrm{k}_{\mathrm{AE,hco3}}}{[\mathrm{HCO}^{-}_{3,\mathrm{e}}]} + \frac{\mathrm{k}_{\mathrm{AE,hco3}} * [\mathrm{Cl}^{-}_{\mathrm{e}}]}{[\mathrm{HCO}^{-}_{3,\mathrm{e}}] * \mathrm{k}_{\mathrm{AE,cl}}} \tag{S.135}$$

$$\mathrm{bb}_{\mathrm{AE}} = 1 + \frac{\mathrm{k}_{\mathrm{AE,cl}}}{[\mathrm{Cl}^{-}_{\mathrm{e}}]} + \frac{\mathrm{k}_{\mathrm{AE,cl}} * [\mathrm{HCO}^{-}_{3,\mathrm{e}}]}{[\mathrm{Cl}^{-}_{\mathrm{e}}] * \mathrm{k}_{\mathrm{AE,hco3}}} \tag{S.136}$$

$$\mathrm{cc_{AE}} = 1 + \frac{\mathrm{k_{AE,cl}}}{[\mathrm{Cl_i^-}]} + \frac{\mathrm{k_{AE,cl}} * [\mathrm{HCO_{3,i}^-}]}{[\mathrm{Cl_i^-}] * \mathrm{k_{AE,hco3}}} \quad (S.137)$$

$$\mathrm{dd_{AE}} = 1 + \frac{\mathrm{k_{AE,hco3}}}{[\mathrm{HCO_{3,i}^-}]} + \frac{\mathrm{k_{AE,hco3}} * [\mathrm{Cl_i^-}]}{[\mathrm{HCO_{3,i}^-}] * \mathrm{k_{AE,cl}}} \quad (S.138)$$

$$\mathrm{s1_{AE}} = \frac{1}{\mathrm{aa_{AE}} + \mathrm{dd_{AE}} * \dfrac{\mathrm{k^+_{AE1}} + \mathrm{k^+_{AE2}}\left(\dfrac{\mathrm{aa_{AE}}}{\mathrm{bb_{AE}}}\right)}{\mathrm{k^-_{AE1}} + \mathrm{k^-_{AE2}}\left(\dfrac{\mathrm{dd_{AE}}}{\mathrm{cc_{AE}}}\right)}} \quad (S.139)$$

$$\mathrm{s6_{AE}} = \frac{1}{\mathrm{dd_{AE}} + \mathrm{aa_{AE}} * \dfrac{\mathrm{k^-_{AE1}} + \mathrm{k^-_{AE2}}\left(\dfrac{\mathrm{dd_{AE}}}{\mathrm{cc_{AE}}}\right)}{\mathrm{k^+_{AE1}} + \mathrm{k^+_{AE2}}\left(\dfrac{\mathrm{aa_{AE}}}{\mathrm{bb_{AE}}}\right)}} \quad (S.140)$$

$$\mathrm{reg_{AE}} = \left(\frac{\mathrm{K_{AE,hi}^{n_{AE,hi}}}}{([\mathrm{H_i^+}]^{\mathrm{n_{AE,hi}}} + \mathrm{K_{AE,hi}^{n_{AE,hi}}})}\right) * \left(\frac{[\mathrm{H_e^+}]^{\mathrm{n_{AE,he}}}}{([\mathrm{H_e^+}]^{\mathrm{n_{AE,he}}} + \mathrm{K_{AE,he}^{n_{AE,he}}})}\right) \quad (S.141)$$

$$\mathrm{V_{AE}} = \mathrm{Vol_{myo}} * \mathrm{reg_{AE}} * \frac{\mathrm{k^-_{AE1}} * \mathrm{s6_{AE}} - \mathrm{k^+_{AE1}} * \mathrm{s1_{AE}}}{60,000} \quad (S.142)$$

Sodium-Bicarbonate Symporter

$$\mathrm{aa_{NBC}} = 1 + \frac{[\mathrm{Na_o^+}]}{\mathrm{k_{NBC,na}}} + \frac{[\mathrm{Na_o^+}] * [\mathrm{HCO_{3,e}^-}]}{\mathrm{k_{NBC,na}} * \mathrm{k_{NBC,hco3}}} \quad (S.143)$$

$$\mathrm{bb_{NBC}} = 1 + \frac{\mathrm{k_{NBC,hco3}}}{[\mathrm{HCO_{3,e}^-}]} + \frac{\mathrm{k_{NBC,na}} * \mathrm{k_{NBC,hco3}}}{[\mathrm{HCO_{3,e}^-}] * [\mathrm{Na_o^+}]} \quad (S.144)$$

$$\mathrm{cc_{NBC}} = 1 + \frac{\mathrm{k_{NBC,hco3}}}{[\mathrm{HCO_{3,i}^-}]} + \frac{\mathrm{k_{NBC,na}} * \mathrm{k_{NBC,hco3}}}{[\mathrm{HCO_{3,i}^-}] * [\mathrm{Na_i^+}]} \quad (S.145)$$

$$\mathrm{dd_{NBC}} = 1 + \frac{[\mathrm{Na_i^+}]}{\mathrm{k_{NBC,na}}} + \frac{[\mathrm{Na_i^+}] * [\mathrm{HCO_{3,i}^-}]}{\mathrm{k_{NBC,na}} * \mathrm{k_{NBC,hco3}}} \quad (S.146)$$

$$\mathrm{s1_{NBC}} = \frac{1}{\mathrm{aa_{NBC}} + \mathrm{dd_{NBC}} * \dfrac{\mathrm{k^+_{NBC1}} + \mathrm{k^+_{NBC2}}\left(\dfrac{\mathrm{aa_{NBC}}}{\mathrm{bb_{NBC}}}\right)}{\mathrm{k^-_{NBC1}} + \mathrm{k^-_{NBC2}}\left(\dfrac{\mathrm{dd_{NBC}}}{\mathrm{cc_{NBC}}}\right)}} \quad (S.147)$$

$$s6_{NBC} = \frac{1}{dd_{NBC} + aa_{NBC} * \frac{k^{-}_{NBC1} + k^{-}_{NBC2}\left(\frac{dd_{NBC}}{cc_{NBC}}\right)}{k^{+}_{NBC1} + k^{+}_{NBC2}\left(\frac{aa_{NBC}}{bb_{NBC}}\right)}} \tag{S.148}$$

$$reg_{NBC} = \left(\frac{[H^{+}_{i}]^{n_{NBC,hi}}}{([H^{+}_{i}]^{n_{NBC,hi}} + K^{n_{NBC,hi}}_{NBC,hi})}\right) * \left(1 - \left(\frac{[H^{+}_{e}]^{n_{NBC,he}}}{([H^{+}_{e}]^{n_{NBC,he}} + K^{n_{NBC,he}}_{NBC,he})}\right)\right) \tag{S.149}$$

$$V_{NBC} = Vol_{myo} * reg_{NBC} * \frac{k^{-}_{NBC1} * s6_{NBC} - k^{+}_{NBC1} * s1_{NBC}}{60,000} \tag{S.150}$$

Chloride-Hydroxide Exhanger

$$aa_{CHE} = 1 + \frac{k_{CHE,oh}}{[OH^{-}_{e}]} + \frac{k_{CHE,oh} * [Cl^{-}_{e}]}{[OH^{-}_{e}] * k_{CHE,cl}} \tag{S.151}$$

$$bb_{CHE} = 1 + \frac{k_{CHE,cl}}{[Cl^{-}_{e}]} + \frac{k_{CHE,cl} * [OH^{-}_{e}]}{[Cl^{-}_{e}] * k_{CHE,oh}} \tag{S.152}$$

$$cc_{CHE} = 1 + \frac{k_{CHE,cl}}{[Cl^{-}_{i}]} + \frac{k_{CHE,cl} * [OH^{-}_{i}]}{[Cl^{-}_{i}] * k_{CHE,oh}} \tag{S.153}$$

$$dd_{CHE} = 1 + \frac{k_{CHE,oh}}{[OH^{-}_{i}]} + \frac{k_{CHE,oh} * [Cl^{-}_{i}]}{[OH^{-}_{i}] * k_{CHE,cl}} \tag{S.154}$$

$$s1_{CHE} = \frac{1}{aa_{CHE} + dd_{CHE} * \frac{k^{+}_{CHE1} + k^{+}_{CHE2}\left(\frac{aa_{CHE}}{bb_{CHE}}\right)}{k^{-}_{CHE1} + k^{-}_{CHE2}\left(\frac{dd_{CHE}}{cc_{CHE}}\right)}} \tag{S.155}$$

$$s6_{CHE} = \frac{1}{dd_{CHE} + aa_{CHE} * \frac{k^{-}_{CHE1} + k^{-}_{CHE2}\left(\frac{dd_{CHE}}{cc_{CHE}}\right)}{k^{+}_{CHE1} + k^{+}_{CHE2}\left(\frac{aa_{CHE}}{bb_{CHE}}\right)}} \tag{S.156}$$

$$V_{CHE} = Vol_{myo} * \frac{k^{-}_{CHE1} * s6_{CHE} - k^{+}_{CHE1} * s1_{CHE}}{60,000} \tag{S.157}$$

Sodium-Hydrogen Exchanger

$$aa_{NHE} = 1 + \frac{k_{NHE,h}}{[H_e^+]} + \frac{k_{NHE,h} * [Na_o^+]}{[H_e^+] * k_{NHE,na}} \tag{S.158}$$

$$bb_{NHE} = 1 + \frac{k_{NHE,na}}{[Na_o^+]} + \frac{k_{NHE,na} * [H_e^+]}{[Na_o^+] * k_{NHE,h}} \tag{S.159}$$

$$cc_{NHE} = 1 + \frac{k_{NHE,na}}{[Na_i^+]} + \frac{k_{NHE,na} * [H_i^+]}{[Na_i^+] * k_{NHE,h}} \tag{S.160}$$

$$dd_{NHE} = 1 + \frac{k_{NHE,h}}{[H_i^+]} + \frac{k_{NHE,h} * [Na_i^+]}{[H_i^+] * k_{NHE,na}} \tag{S.161}$$

$$s1_{NHE} = \frac{1}{aa_{NHE} + dd_{NHE} * \frac{k^+_{NHE1} + k^+_{NHE2}\left(\frac{aa_{NHE}}{bb_{NHE}}\right)}{k^-_{NHE1} + k^-_{NHE2}\left(\frac{dd_{NHE}}{cc_{NHE}}\right)}} \tag{S.162}$$

$$s6_{NHE} = \frac{1}{dd_{NHE} + aa_{NHE} * \frac{k^-_{NHE1} + k^-_{NHE2}\left(\frac{dd_{NHE}}{cc_{NHE}}\right)}{k^+_{NHE1} + k^+_{NHE2}\left(\frac{aa_{NHE}}{bb_{NHE}}\right)}} \tag{S.163}$$

$$reg_{NHE} = \left(\frac{[H_i^+]^{n_{NHE,hi}}}{([H_i^+]^{n_{NHE,hi}} + K_{NHE}^{n_{NHE,hi}})}\right) * \left(1 - \frac{[H_e^+]^{n_{NHE,he}}}{([H_e^+]^{n_{NHE,he}} + K_{NHE}^{n_{NHE,he}})}\right) \tag{S.164}$$

$$V_{NHE} = modifier_{NHE} * Vol_{myo} * reg_{NHE} * \frac{k^-_{NHE1} * s6_{NHE} - k^+_{NHE1} * s1_{NHE}}{60,000} \tag{S.165}$$

Carbon Dioxide

$$V_{CO_2} = 1.17 * Vol_{myo} * ([CO_{2,e}] - [CO_{2,i}]) \tag{S.166}$$

$$V_{hyd,e} = Vol_{external}(k^+_{hyd}[CO_{2,e}] - k^-_{hyd}[HCO^-_{3,e}][H_e^+]) \tag{S.167}$$

$$V_{hyd,i} = (Vol_{myo} + Vol_{sr}) * (k^+_{hyd}[CO_{2,i}] - k^-_{hyd}[HCO^-_{3,i}] * [H_i^+]) \tag{S.168}$$

$$\frac{d[CO_{2,i}]}{dt} = \frac{V_{CO2}}{Vol_{myo}} - \frac{V_{hyd,i}}{Vol_{myo} + Vol_{sr}} \quad (S.169)$$

During pre-ischemia, $[CO_{2,e}]$ is constant:

$$[CO_{2,e}] = ppCO_2 * CO_{2sol} * p_{atm} \quad (S.170)$$

During ischemia:

$$[CO_{2,e}]_t = [CO_{2,e}]_{t\text{-}\Delta t} - \frac{V_{CO2} + V_{hyd,e}}{Vol_{external}} \Delta t \quad (S.171)$$

During reperfusion:

$$[CO_{2,e}]_t = [CO_{2,e}]_{t\text{-}\Delta t} - \frac{[CO_{2,e}]_{t\text{-}\Delta t} - [CO_{2,e}]_{preischemic}}{3.75x10^4} \Delta t \quad (S.172)$$

Bicarbonate

$$\frac{d[HCO^-_{3,i}]}{dt} = \frac{V_{hyd,i}}{Vol_{myo} + Vol_{sr}} + \frac{V_{NBC} - V_{AE}}{Vol_{myo}} \quad (S.173)$$

During pre-ischemia, $[HCO^-_{3,e}]$ is constant. During ischemia:

$$[HCO^-_{3,e}]_t = [HCO^-_{3,e}]_{t\text{-}\Delta t} + \frac{V_{hyd,e} - V_{NBC} + V_{AE}}{Vol_{external}} \Delta t \quad (S.174)$$

During reperfusion:

$$[HCO^-_{3,e}]_t = [HCO^-_{3,e}]_{t\text{-}\Delta t} - \frac{[HCO^-_{3,e}]_{t\text{-}\Delta t} - [HCO^-_{3,e}]_{preischemic}}{3.75x10^4} \Delta t \quad (S.175)$$

pH

$$\beta_e = \log_{10}\left(10^{-pHe} + \frac{[ib_1] * 10^{(pK1\text{-}pHe)}}{(1 + 10^{(pK1\text{-}pHe)})^2} + \frac{[ib_2] * 10^{(pK2\text{-}pHe)}}{(1 + 10^{(pK2\text{-}pHe)})^2}\right) \quad (S.176)$$

$$\beta_i = \log_{10}\left(10^{-pHi} + \frac{[ib_1] * 10^{(pK1\text{-}pHi)}}{(1 + 10^{(pK1\text{-}pHi)})^2} + \frac{[ib_2] * 10^{(pK2\text{-}pHi)}}{(1 + 10^{(pK2\text{-}pHi)})^2}\right) \quad (S.177)$$

During pre-ischemia, pH_e is constant. During ischemia:

$$pH_{e,\ t} = pH_{e,\ t\text{-}\Delta t} - \frac{1}{\beta_e}\left(\frac{V_{NHE} - V_{CHE} + V_{hyd,e}}{Vol_{external}}\right) \Delta t \quad (S.178)$$

During reperfusion:

$$pH_{e,\ t} = pH_{e,\ t\text{-}\Delta t} + \frac{(7.40 - pH_{e,\ t\text{-}\Delta t})}{1.5x10^5} \Delta t \quad (S.179)$$

During pre-ischemia and reperfusion:

$$pH_{i,\ t} = pH_{i,\ t\text{-}\Delta t} - \frac{1}{\beta_i}\left(\frac{-V_{NHE} + V_{CHE}}{Vol_{myo}} + \frac{V_{hyd,i}}{Vol_{myo} + Vol_{sr}}\right) \Delta t \quad (S.180)$$

During ischemia:

$$pH_i = 6.18507 - 0.56697e^{-0.19015t} + 1.5377e^{-0.18462t} \quad (S.181)$$

Ion Concentrations

$$[H_e^+] = 10^{(3\text{-}pH_e)} \tag{S.182}$$

$$[H_i^+] = 10^{(3\text{-}pH_i)} \tag{S.183}$$

$$[OH_e^-] = 10^{(-11+pH_e)} \tag{S.184}$$

$$[OH_i^-] = 10^{(-11+pH_i)} \tag{S.185}$$

$$I_{Na,total} = I_{Na} + I_{Na,b} + I_{Na,l} + I_{Ca(L),Na} + 3 * I_{NaK} + 3 * I_{NaCa} \tag{S.186}$$

$$I_{K,total} = I_{Kr} + I_{Ks} + I_{K1} + I_{Kp} + I_{K(ATP)} + I_{Ca(L),K} - 2 * I_{NaK} \tag{S.187}$$

$$I_{Ca,total} = I_{Ca(L)} + I_{Ca,b} + I_{Ca,t} + I_{p(Ca)} - 2 * I_{NaCa} \tag{S.188}$$

$$I_{Cl,total} = I_{Cl} \tag{S.189}$$

$$\frac{d[Na_i^+]}{dt} = \frac{-I_{Na,total} * C_m * A_{cap}}{F * z_{Na} * Vol_{myo}} + \frac{V_{NHE} + V_{NBC}}{Vol_{myo}} \tag{S.190}$$

$$\frac{d[K_i^+]}{dt} = \frac{-(I_{K,total} + I_{stim}) * C_m * A_{cap}}{F * z_K * Vol_{myo}}, \tag{S.191}$$

$$\beta_{cai} = \beta_{TRPN} + \beta_{CMDN} - 1 \tag{S.192}$$

$$\frac{d[Ca_i^{2+}]}{dt} = -\frac{1}{\beta_{cai}}\left(\frac{I_{Ca,total} * C_m * A_{cap}}{F * z_{Ca} * Vol_{myo}} + \left((V_{SERCA} - I_{leak})\frac{Vol_{nsr}}{Vol_{myo}}\right) - \left((I_{relcicr} + I_{reljsrol})\frac{Vol_{jsr}}{Vol_{myo}}\right)\right) \tag{S.193}$$

$$\frac{d[Cl_i^-]}{dt} = \frac{-I_{Cl,total} * C_m * A_{cap}}{F * z_{Cl} * Vol_{myo}} + \frac{V_{CHE} + V_{AE}}{Vol_{myo}} \tag{S.194}$$

During pre-ischemia, $[Na^+{}_o]$, $[K^+{}_o]$, and $[Ca^{2+}{}_o]$ are constant. During ischemia:

$$[Na_o^+]_t = [Na_o^+]_{t\text{-}\Delta t} + \left(\frac{I_{Na,total} * C_m * A_{cap}}{F * z_{Na} * Vol_{external}} - \frac{V_{NHE} + V_{NBC}}{Vol_{external}}\right)\Delta t \tag{S.195}$$

$$[K_o^+]_t = [K_o^+]_{t\text{-}\Delta t} + \frac{I_{K,total} * C_m * A_{cap}}{F * z_K * Vol_{external}} \Delta t \tag{S.196}$$

$$[Ca_o^{2+}]_t = [Ca_o^{2+}]_{t\text{-}\Delta} t + \frac{I_{Ca,total} * C_m * A_{cap}}{F * z_{Ca} * Vol_{external}} \Delta t \tag{S.197}$$

During reperfusion:

$$[Na_o^+]_t = [Na_o^+]_{t\text{-}\Delta t} - \frac{[Na_o^+]_{t\text{-}\Delta t} - [Na_o^+]_{preischemic}}{3.75x10^4} \Delta t \tag{S.198}$$

$$[K_o^+]_t = [K_o^+]_{t\text{-}\Delta t} - \frac{[K_o^+]_{t\text{-}\Delta t} - [K_o^+]_{preischemic}}{3.75x10^4} \Delta t \tag{S.199}$$

$$[Ca_o^{2+}]_t = [Ca_o^{2+}]_{t\text{-}\Delta t} - \frac{[Ca_o^{2+}]_{t\text{-}\Delta t} - [Ca_o^{2+}]_{preischemic}}{3.75x10^4} \Delta t \tag{S.200}$$

Volume Regulation

During pre-ischemia, $\prod_i$ is constant at 310.0. During ischemia:

$$\prod_i = 310 + t \tag{S.201}$$

During reperfusion:

$$\prod_{i,\ t} = \prod_{i,\ t\text{-}\Delta 1} - \frac{\prod_{i,\ t\text{-}\Delta t} - 310}{3.75x10^4 ms} \Delta t \tag{S.202}$$

Calculated once at beginning of simulation:

$$X_e^{zi\text{-}} = Vol_{external} * \left(\prod_e - [Na_o^+] - [K_o^+] - [Ca_o^{2+}] - [Cl_e^-] \right) \tag{S.203}$$

During ischemia and reperfusion (constant during pre-ishemia):

$$X_i^{zi\text{-}} = Vol_{myo} * \left(\prod_i - [Na_i^+] - [K_i^+] - [Ca_i^{2+}] - [Cl_i^-] \right) \tag{S.204}$$

$$V_{H_2O} = 10 * Lp * R * temp * (([Na_i^+] + [K_i^+] + [Ca_i^{2+}] + [Cl_i^-] + \frac{[X_i^{zi\text{-}}]}{Vol_{myo}}) - ([Na_o^+] + [K_o^+] + [Ca_o^{2+}] + [Cl_e^-] + \frac{[X_e^{zi\text{-}}]}{Vol_{external}})) \tag{S.205}$$

$$dVol_{cell} = A_{cap} * V_{H_2O} * \Delta t \tag{S.206}$$

$$Vol_{myo} = Vol_{cell} * 0.68 \tag{S.207}$$

$$Vol_{sr} = Vol_{cell} * 0.06 \tag{S.208}$$

$$Vol_{nsr} = Vol_{cell} * 0.0552 \tag{S.209}$$

$$Vol_{jsr} = Vol_{cell} * 0.0048 \tag{S.210}$$

$$Vol_{external} = Vol_{total} - Vol_{cell} \tag{S.211}$$

Voltage Calculations

Calculate $\mathrm{Proton_{ref}}$ and $\mathrm{Hib_{ref}}$ at beginning of simulation:

$$[\mathrm{Proton_{ref}}] = 10^{3\text{-}\mathrm{pH_i}} \tag{S.212}$$

$$[\mathrm{Hib_{ref}}] = \frac{[\mathrm{ib_1}] * 10^{\text{-}\mathrm{pH_i}}}{10^{\text{-}\mathrm{pH_i}} + 10^{\text{-}\mathrm{pK1}}} + \frac{[\mathrm{ib_2}] * 10^{\text{-}\mathrm{pH_i}}}{10^{\text{-}\mathrm{pH_i}} + 10^{\text{-}\mathrm{pK2}}} \tag{S.213}$$

$$[\mathrm{Hib_1}] = \frac{[\mathrm{ib_1}] * 10^{\text{-}\mathrm{pHi}}}{10^{\text{-}\mathrm{pHi}} + 10^{\text{-}\mathrm{pK1}}} \tag{S.214}$$

$$[\mathrm{Hib_2}] = \frac{[\mathrm{ib_2}] * 10^{\text{-}\mathrm{pHi}}}{10^{\text{-}\mathrm{pHi}} + 10^{\text{-}\mathrm{pK2}}} \tag{S.215}$$

$$[\mathrm{Anion^{\text{-}}}] = (10^{3\text{-}\mathrm{pHi}} - [\mathrm{Proton_{ref}}]) + ([\mathrm{Hib_1}] + [\mathrm{Hib_2}] - [\mathrm{Hib_{ref}}]) \tag{S.216}$$

$$\begin{aligned} \mathrm{C_0} = &-\frac{\mathrm{V} * \mathrm{A_{cap}} * \mathrm{C_m}}{\mathrm{Vol_{myo}} * \mathrm{F}} + [\mathrm{Na_i^+}] + [\mathrm{K_i^+}] - [\mathrm{Cl_i^{\text{-}}}] - [\mathrm{HCO^{\text{-}}_{3,i}}] + ([\mathrm{Proton_{ref}}] - [\mathrm{Hib_{ref}}]) - [\mathrm{OH_i^{\text{-}}}] - [\mathrm{Anion^{\text{-}}}] + \\ &\mathrm{z_{Ca}} * \left(([\mathrm{Ca_i^{2+}}] + [\mathrm{TRPN}] + [\mathrm{CMDN}]) + [\mathrm{NSR}] * \left(\frac{\mathrm{Vol_{nsr}}}{\mathrm{Vol_{myo}}}\right) + ([\mathrm{JSR} + [\mathrm{CSQN}]) * \left(\frac{\mathrm{Vol_{jsr}}}{\mathrm{Vol_{myo}}}\right)\right) \end{aligned} \tag{S.217}$$

$$\begin{aligned} \mathrm{V} = &\frac{\mathrm{Vol_{myo}} * \mathrm{F}}{\mathrm{A_{cap}} * \mathrm{C_m}} * \Big(- \mathrm{C_0} + [\mathrm{Na_i^+}] + [\mathrm{K_i^+}] - [\mathrm{Cl_i^{\text{-}}}] - [\mathrm{HCO^{\text{-}}_{3,i}}] + ([\mathrm{Proton_{ref}}] - [\mathrm{Hib_{ref}}]) - [\mathrm{OH_i^{\text{-}}}] - [\mathrm{Anion^{\text{-}}}] + \\ &\mathrm{z_{Ca}} * \left(([\mathrm{Ca_i^{2+}}] + [\mathrm{TRPN}] + [\mathrm{CMDN}]) + [\mathrm{NSR}] * \left(\frac{\mathrm{Vol_{nsr}}}{\mathrm{Vol_{myo}}}\right) + ([\mathrm{JSR} + [\mathrm{CSQN}]) * \left(\frac{\mathrm{Vol_{jsr}}}{\mathrm{Vol_{myo}}}\right)\right) \Big) \end{aligned} \tag{S.218}$$

$$\mathrm{dvdtnew} = \frac{\mathrm{V_t} - \mathrm{V_{\Delta t}}}{\Delta \mathrm{t}} \tag{S.219}$$

Phosphometabolites

During pre-ischemia, all metabolites are held at their initial values. During ischemia:

$$[\mathrm{ATP}] = 0.0006549\mathrm{t}^3 - 0.02305\mathrm{t}^2 - 0.104837\mathrm{t} + 7.216 \tag{S.220}$$

$$[\mathrm{PCr}] = -0.01259 + 12.339\mathrm{e}^{\text{-}0.92559\mathrm{t}} + 0.96819\mathrm{e}^{\text{-}0.078496\mathrm{t}} \tag{S.221}$$

During reperfusion:

$$[\mathrm{ATP}]_\mathrm{t} = [\mathrm{ATP}]_{\mathrm{t}\text{-}\Delta\mathrm{t}} - \frac{[\mathrm{ATP}]_{\mathrm{t}\text{-}\Delta\mathrm{t}} - 0.4[\mathrm{ATP_{preischemic}}]}{5\mathrm{x}10^3\mathrm{ms}}\Delta\mathrm{t} \tag{S.222}$$

$$[\mathrm{PCr}]_{\mathrm{t}} = [\mathrm{PCr}]_{\mathrm{t\text{-}\Delta t}} - \frac{[\mathrm{PCr}]_{\mathrm{t\text{-}\Delta t}} - 0.75[\mathrm{ion_{preischemic}}]}{5\mathrm{x}10^3\mathrm{ms}}\Delta\mathrm{t} \tag{S.223}$$

During ischemia and reperfusion:

$$[\mathrm{Cr}] = 22.2 - [\mathrm{PCr}] \tag{S.224}$$

$$[\mathrm{ADP}] = \frac{[\mathrm{ATP}] * [\mathrm{Cr}]}{[\mathrm{PCr}] * [\mathrm{H_i^+}] * 1.66\mathrm{x}10^6} \tag{S.225}$$

$$[\mathrm{AMP}] = \frac{[\mathrm{ADP}] * [\mathrm{ADP}] * 1.05}{[\mathrm{ATP}]} \tag{S.226}$$

$$[\mathrm{Pi_{free}}] = [\mathrm{Pi_{total}} - (3 * [\mathrm{ATP}] + 2 * [\mathrm{ATP}] + [\mathrm{AMP}] + [\mathrm{PCr}]) \tag{S.227}$$

Miscellaneous

$$\mathrm{E_{Na}} = \frac{\mathrm{R} * \mathrm{temp}}{\mathrm{F}} * \log\left(\frac{[\mathrm{Na_o^+}]}{[\mathrm{Na_i^+}]}\right) \tag{S.228}$$

$$\mathrm{E_{Ca}} = \frac{\mathrm{R} * \mathrm{temp}}{2 * \mathrm{F}} * \log\left(\frac{[\mathrm{Ca_o^{2+}}]}{[\mathrm{Ca_i^{2+}}]}\right) \tag{S.229}$$

$$\mathrm{E_{K}} = \frac{\mathrm{R} * \mathrm{temp}}{\mathrm{F}} * \log\left(\frac{[\mathrm{K_o^+}]}{[\mathrm{K_i^+}]}\right) \tag{S.230}$$

$$\mathrm{E_{Ks}} = \frac{\mathrm{R} * \mathrm{temp}}{\mathrm{F}} * \log\left(\frac{[\mathrm{K_o^+}] + \mathrm{prnak} * [\mathrm{Na_o^+}]}{[\mathrm{K_i^+}] + \mathrm{prnak} * [\mathrm{Na_i^+}]}\right) \tag{S.231}$$

If dvdtnew $>$ 10.0 AND $\mathrm{t_{cicr}} > 10.0$ AND flag = true, then set flag = false.

If V $>$ -35.0 AND $\mathrm{dI_{Ca,total\ t}} > \mathrm{dI_{Ca,total\ t\text{-}\Delta t}}$ AND flag = false, then:
set flag = true
set $\mathrm{t_{cicr}} = 0$

If [CSQN] $\geq$ csqnth AND $\mathrm{t_{jsrol}} \geq 50$, then reset $\mathrm{t_{jsrol}} = 0$.

Notes on experimental data used in determination of model parameters

The following pertains to model parameters cited from [1]:

AE, CHE, NBC, and NHE:

[8]: guinea pig papillary muscle and sheep Purkinje fiber, 34-37 degrees C.

[9]: isolated rabbit ventricular myocytes, 35-37 degrees C.

[10]: isolated guinea pig ventricular myocytes, 37 degrees C.

[11]: isolated guinea pig ventricular myocytes, 37 degrees C.

[12]: sheep Purkinje fiber, 37 degrees C.

[7]: sheep Purkinje fiber, 37 degrees C.

pH Dependence of SR Calcium Release:

[13]: canine cardiac muscle SR fractions.

pH Dependence of Na-Ca Exchange:

[14]: guinea pig ventricular cell excised pathches.

pH Dependence of Calcium Binding to Troponin:

[15]: canine cardiac myofibrils, 22 degrees C.

[16]: XL1 cells transformed with mouse Troponin I cDNA.

pH Regulation:

[17]: squid giant axon, 23 degrees C.

[18]: isolated guinea pig ventricular myocytes, 37 degrees C.

[10]: isolated guinea pig ventricular myocytes, 37 degrees C.

The following pertains to model parameters cited from [3]:

NaK Pump:

Parameter values were adjusted to correspond to a temperature of 37 degrees C using a Q_{10} of 2.6.

$rate^{+}_{NaK1}$, $rate^{-}_{NaK1}$, sensitivity to $[Na^{+}_{o}]$ and [ADP] [19]: isolated rat myocytes, 23 degrees C.

$rate^{+}_{NaK2}$, $rate^{-}_{NaK2}$ [20]: rabbit kidney fragments adsorbed on lipid bilayers, 20 degrees C.

$rate^{-}_{NaK3}$ [21]: porcine kidney outer medulla, 37 degrees C.

$rate^{+}_{NaK4}$, $rate^{-}_{NaK4}$ [22]: rabbit kidney outer medulla, 37 degrees C.

$density_{NaK}$ [23]: isolated guinea pig and rat myocytes, 35 degrees C.

Sensitivity to $[Na^{+}_{i}]$ [24]: rabbit ventricular myocytes, 22 degrees C.

Sensitivity to membrane potential and $[K^{+}_{o}]$ [25]: guinea pig membrane excised patches, 36 degrees C.

Sensitivity to changes in [ATP] [26]: guinea pig excised patches.

Other experimental data sets used:

[27–29]: isolated guinea pig ventricular myocytes, 36 degrees C.

$I_{K(ATP)}$:

[30]: guinea pig ventricular myocytes, 36 degrees C.

[31]: guinea pig isolated ventricular myocytes, room temperature.

[32]: COSm6 cells transfected with mouse Kir6.2, 20-22 degrees C.

[33]: COSm6 cells transfected with mouse Kir6.2, 25 degrees C.

[34]: HEK cells transfected with Kir6.2 and SUR1.

[35]: mouse $I_{K(ATP)}$ isolted from pancreatic beta and HIT cells.

[36]: *Xenopus* oocytes transfected with mouse Kir6.2 and rat SUR1.

[37]: COS-7 cells transfected with hamster SUR1, 20-24 degrees C.

Volume Regulation:

[38]: slices of canine left ventricle and papillary muscle, 37 degrees C.

[39]: porcine, transmural biopsies from intact beating heart.

pH and phosphometabolites:

ATP, PCr, and pH [40]: perfused guinea pig hearts, 37 degrees C.

ATP, PCr, and Cr [41]: guinea pig isolated myocytes, atria, and saline perfused hearts, 37 degrees C.

P_i [42]: perfused guinea pig hearts.

The following pertains to model parameters cited from [6]:

Data from three studies were used to constrain model parameters:

[43]: $Ca^{2+}{}_i$ uptake versus pH (Fig. 5); mouse SERCA2a microsomes, experiments performed at 37 degrees C, [ATP] = 3mM, [ADP] = 0mM, [P_i] = 0mM, [$Ca^{2+}{}_i$] = 0mM, [$Ca^{2+}{}_{SR}$] = 0.001mM.

[43]: $Ca^{2+}{}_i$ uptake versus [$Ca^{2+}{}_i$] at different pH (Fig. 4); mouse SERCA2a microsomes, experiments performed at 37 degrees C, [ATP] = 3mM, [ADP] = 0mM, [P_i] = 0mM, [$Ca^{2+}{}_i$] = variable, [$Ca^{2+}{}_{SR}$] = 0mM.

[44]: level of back phosphorylation versus [P_i] (Fig. 6); rabbit SERCA1a nonnative cell experession, 25 degrees C, pH 6, [ATP] = 0mM, [ADP] = 0mM, [P_i] = variable, [$Ca^{2+}{}_i$] = 0mM, [$Ca^{2+}{}_{SR}$] = 0mM.

[45]: ATPase activity versus ATP and ADP (Fig. 7), rabbit SERCA1a solubilized SR fragments, 10 degrees C, pH 7, [ATP] = variable, [ADP] = variable, [P_i] = 0mM, [$Ca^{2+}{}_i$] = 5mM, [$Ca^{2+}{}_{SR}$] = 5mM.

In the absence of reliable Q_{10} data, the authors in [6] chose normalize the data to a maximum cycle rate of 5 s^{-1} under the following "optimal" conditions: pH = 7.2, [ATP] = 5mM, [ADP] = 0mM, [P_i] = 0mM, [$Ca^{2+}{}_i$] = 0mM, [$Ca^{2+}{}_{SR}$] = 10uM.

The following pertains to model parameters cited from [5]:

The experiments used to determine $k_{1/2}$ for ATP binding and the Hill coefficient of 2.6 were performed using isolated guinea pig ventricular cells at a temperature between 35 and 36 degrees C [30].

The following pertains to model parameters cited from [4]:

The data used to inform alterations in sodium conductance and late sodium current came from experiments performed on isolated guinea pig myocytes at 37 degrees C [46].

Summary of Ischemia and Reperfusion Simulation Protocols

During pre-ischemia, extracellular species and pH are held constant, the assumption being that they are in equilibrium with a much larger volume. During ischemia, when the extracellular compartment is assumed to be isolated, concentrations and pH in the extracellular compartment are allowed to vary as described by the equations cited in the table below and in Supplement 2. Also, a variable representing intracellular osmolarity is increased, favoring inward water flux, thereby increasing and decreasing the intra- and extracellular volumes, respectively. During reperfusion, when extracellular fluid is washed out and replaced with "normal" fluid from the surrounding non-ischemia tissue, we return the values representing extracellular species concentration and pH to normal over a specified time. Intracellular osmolarity is returned to normal, as well.

Also, during ischemia a decrease in intracellular pH, as well as the concentrations of ATP and phosphocreatine, are imposed on the system per the equations cited in the table below.

	Ischemia	Reperfusion
pH_i	Prescribed by Eq. S181	Calculated by Eq. S180
pH_e	Calculated by Eq. S178	Prescribed by Eq. S179
$[CO_{2,e}]$	Calculated by Eq. S171	Prescribed by Eq. S172
$[HCO^{-}_{3,e}]$	Calculated by Eq. S174	Prescribed by Eq. S175
$[Na^{+}_{o}]$	Calculated by Eq. S195	Prescribed by Eq. 198
$[K^{+}_{o}]$	Calculated by Eq. S196	Prescribed by Eq. 199
$[Ca^{2+}_{o}]$	Calculated by Eq. S197	Prescribed by Eq. 200
$\prod_i$	Prescribed by Eq. S201	Prescribed by Eq. S202
[ATP]	Prescribed by Eq. S220	Prescribed by Eq. S222
[PCr]	Prescribed by Eq. S221	Prescribed by Eq. S223
$modifier_{Inal}$	0.00018	0.00007
g_{Na}	14.4 mS/uF	16.0 mS/uF

Table S2: Summary of ischemia and reperfusion protocols.

Data from several different experimental sources were cited in the manuscript. Details of the relevant experimental methods are provided below.

The experiments cited in discussions of intracellular pH (including Figure 3A in the manuscript), ATP and PCr [40] were performed with Langendorff-perfused female guinea pig hearts. Experiments are performed at at temperature of 37 degrees Celsius and whole hearts were perfused with standard Krebs-Henseleit buffer. Changes in pH and phosphometabolite concentrations were determined by NMR spectroscopy. Global ischemia was induced by clamping the perfusion line.

Figure 3B in the manuscript cites control data from experiments detailed in [47]. Male guinea pig hearts were used and a temperature of 37 degrees Celsius was maintained during perfusion of whole hearts. pH measurements before, during, and after global ischemia were made using a pH electrode inserted into the left ventricle. Hearts were spontaneously beating and perfused with Krebs-Henseleit solution of the following composition (in mM): NaCl 118, KCl 4.7, $CaCl_2$ 2.5, $MgSO_4$ 1.2, KH_2PO_4 1.2, $NaHCO_3$ 25, and glucose 10. Buffer was aerated with 95 percent oxygen/5 percent CO_2 (pH 7.5)

Figure 3C in the manuscript and discussions of intracellular sodium and calcium cite data from experiments performed with guinea pig hearts [48]. Calcium and sodium measurements using fluorescent dyes were made from the left ventricles of intact hearts. Hearts were perfused at a temperature of 37.2 degrees Celsius with Krebs-Ringer solution equilibrated with 97 percent oxygen/3 percent carbon dioxide (pH 7.39) and composed of the following (in mM): 137 Na^+, 5 K^+, 1.2 Mg^{2+}, 2.5 Ca^{2+}, 134 Cl^-, 15.5 HCO_3^-, 1.2 $H_2PO_4^-$, 11.5 glucose, 2 pyruvate, 16 mannitol, 0.05 EDTA, 0.1 probenecid, and 5U/l insulin.

The porcine experiment cited in the discussion of extracellular pH recovery [49] consisted of using pH-sensitive electrodes inserted into the LAD of hearts in live anesthetized pigs, ventilated to maintain arterial oxygen saturation greater than 95 percent, arterial P_{CO_2} 35-45 mm Hg, and arterial pH at 7.35-7.45. Within the same discussion, canine data was referenced from a study [50] in which pH measurements were made using a blood gas analyzer from coronary venous blood samples. The dogs in this study were anesthetized and the arterial pH, P_{O_2} and P_{CO_2} before the start of experimental protocols were 7.40, 104 mm Hg, and 37.5 mm Hg, respectively.

The estimate of how much time is required for extracellular ions to return to preischemic concentrations comes from measurements of extracellular potassium made in Langendorff-perfused guinea pig hearts [51]. Ischemia was induced by clamping of the aorta. Ion concentrations were made either by flame photometry or an ion-selective probe. The temperature range was 31-33 degrees Celsius, although temperature was reported to remain constant during individual experiments. Perfusion fluid contained washed bovine erythrocytes (hemoglobin concentration of 8 g/100 ml), dextran (mol wt of 70,000, 4 g/100 ml), insulin (1 U/l), heparin (400 U/l) and Tyrode's solution of the following composition (in mM): Na^+ 149, K^+ 4.5, Mg^{2+} 0.49, Ca^{2+} 1.8, Cl^- 145.8, HCO_3^- 11.9, $H_2PO_4^-$ 0.4, and glucose, 20. pH was between 7.34 and 7.37.

Figure Legends

Figure S1.

Analysis of sodium influx under three different conditions. (A) Sodium influx through all seven components in the model quantified as the change in concentration per minute that would occur in the absence of equivalent efflux via the sodium-potassium exchanger. (B) Percentage of total sodium influx through each of the seven components. For each of the three conditions, the model was allowed to reach steady state and sodium current through each of the seven components was analyzed for the last minute of the simulation. The model was either paced continuously at a rate of 1 Hz under pre-ischemic conditions (blue), was left at rest under pre-ischemic conditions (red), or was left at rest with intracellular pH set to 6.9 (green). As was expected, total sodium influx was highest when the cell was paced, and was higher at rest during mild acidosis than normal pH, due in part to increased flux through the sodium-hydrogen exchanger (NHE) and sodium-bicarbonate symporter (NBC)under acidosis. Also, as expected, there was less sodium-calcium exchange (NCX) activity when the cell was at rest, and even less when the resting cell experienced a lower pH. Other abbreviations: fast inward sodium current (INa), background sodium current (INa,b), late sodium current (INa,l), and sodium current through the L-type calcium channel (Ilcana).

Figure S2.

Intracellular sodium concentration profiles from simulations performed with the aim or investigating strategies that may improve $[Na]_i$ overload during reperfusion. The default ischemia-reperfusion simulation is provided as a reference (black). In the simulation shown in blue, ATP concentration was allowed to recover to the preischemic value during reperfusion (using the same constant), as opposed to being restricted to a maximum recovery of 40 percent of the preischemic value. In the simulation shown in green, NaK exchanger activity was doubled, starting at the moment of initial reperfusion. Both strategies markedly reduced the amount of $[Na]_i$ overload during reperfusion.

Figure S3.

Results of a sensitivity analysis of the sodium-potassium exchanger. For each simulation, one of 20 fixed parameters was deviated 10 percent above or below the default value and the model was run until it reached steady state, at which point the intracellular sodium concentration was recorded. Sensitivity values are reported for each of the 20 parameters. The value on the vertical axis represents the percent change in steady state concentration that results when a given parameter is increased by 1 percent. For example, the most sensitive parameter, the binding constant named $K_{\mathrm{dNaK,nai2}}$, would cause a 0.62 percent increase in steady state sodium concentration when increased by 1 percent. Parameter names, values, units, and meanings can be found in Supplement 1: Symbols and Values.

Figure S4.

The relationship between pacing frequency and steady state intracellular sodium concentration in two models and experimental data. Steady state sodium concentrations at different frequencies are compared between the present model (blue), the LRd model of guinea pig cardiomyocyte physiology (red), and data obtained from guinea pig papillary muscle (Wang *et al*, 1998) (black). Numbers for the LRd results and Wang experimental data were extracted from Figure 4 of [52] using DigitizeIt software (share-it!, Denmark).

Figure S5.

Extracellular pH (A) and intracellular sodium concentration (B) during simulated ischemia (gray region) and reperfusion. Identical conditions were simulated with versions of the model that lacked allosteric NHE regulation by extracellular protons (black) and with extracellular regulation implemented (blue). The cell was constantly simulated at a rate of 3 Hz in both simulations. Experimental studies suggest that after 20 minutes of ischemia, extracellular pH decreases to about 6.0 (see Figure 3 in the manuscript). Implementation of extracellular NHE regulation, in which flux is reduced as extracellular pH decreases, improves the extracellular pH profile of our model, reaching approximately 5.8 after 20 minutes of ischemia, (up from approximately 5.4), and exhibiting a less steep decrease in early ischemia, consistent with the data shown in Figure 3. However, at this pacing frequency, the initial accumulation of intracellular sodium during early ischemia is lost as extracellular acidosis develops and NHE flux decreases.

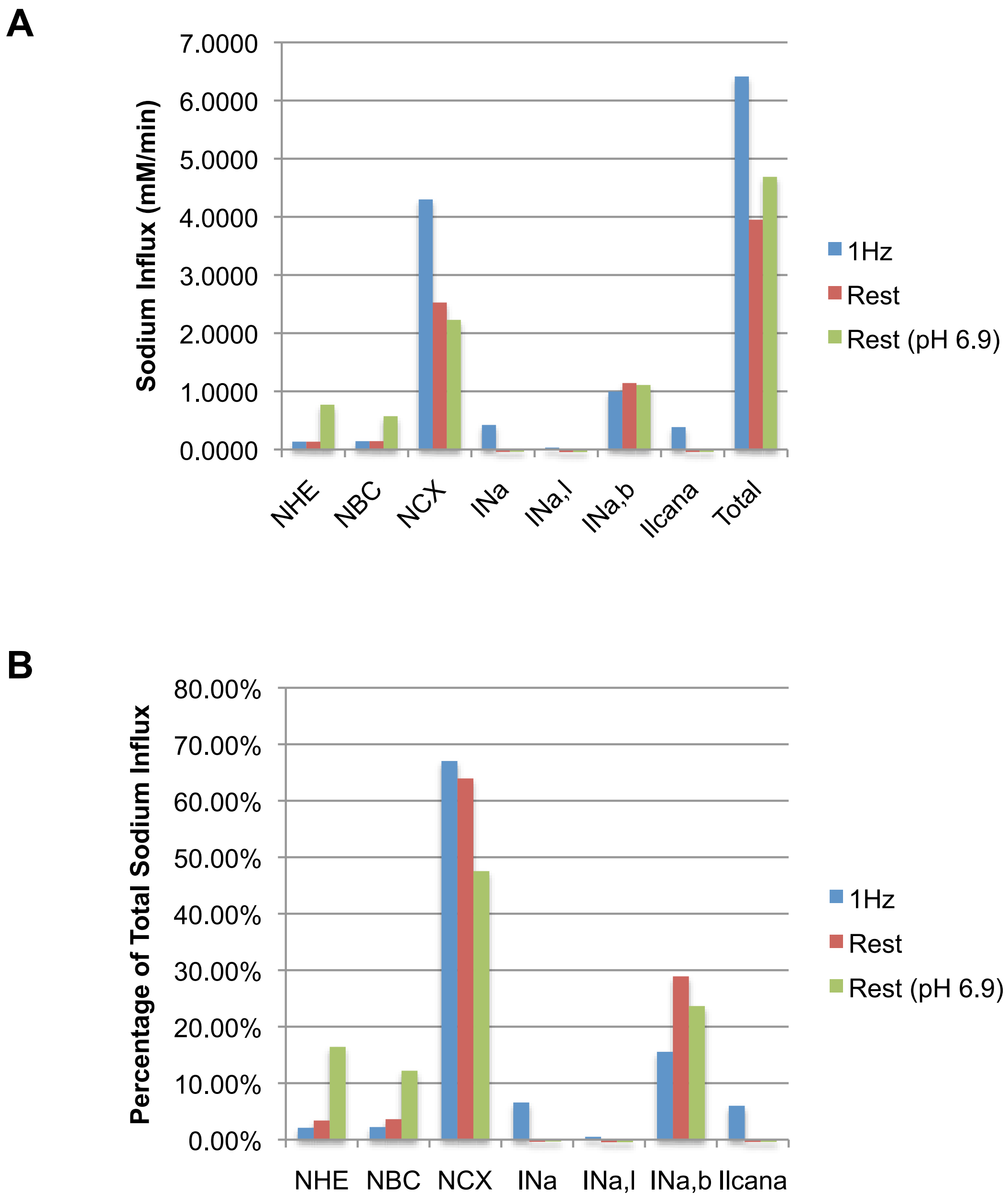
A
7.0000
6.0000
5.0000
4.0000
3.0000
2.0000
1.0000
0.0000
Sodium Influx (mM/min)
NHE
NBC
NCX
INa
INa,l
INa,b
Ilcana
Total
1Hz
Rest
Rest (pH 6.9)
B
80.00%
70.00%
60.00%
50.00%
40.00%
30.00%
20.00%
10.00%
0.00%
Percentage of Total Sodium Influx
NHE
NBC
NCX
INa
INa,l
INa,b
Ilcana
1Hz
Rest
Rest (pH 6.9)

Figure S1:

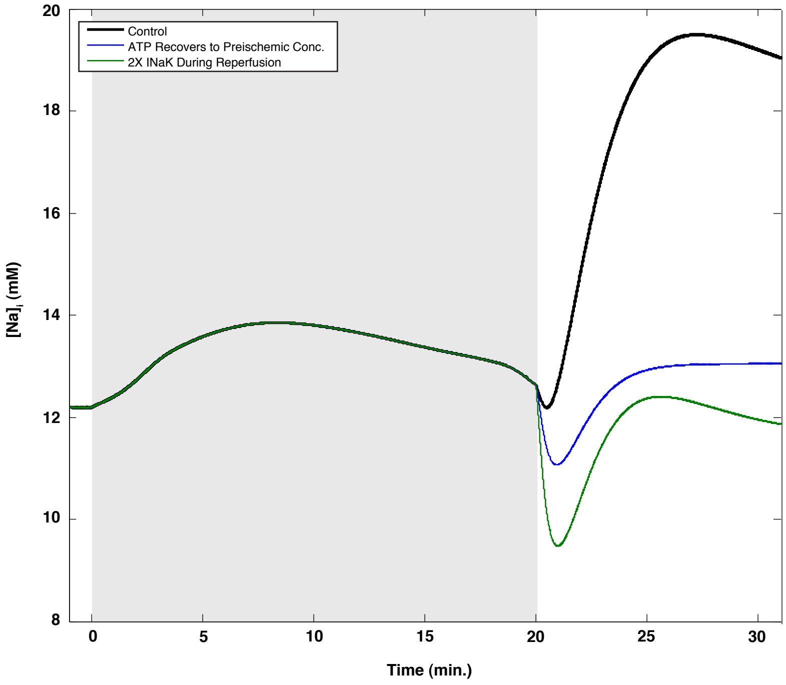
Control
ATP Recovers to Preischemic Conc.
2X INaK During Reperfusion
[Na]i (mM)
Time (min.)
20
18
16
14
12
10
8
0
5
10
15
20
25
30

Figure S2:

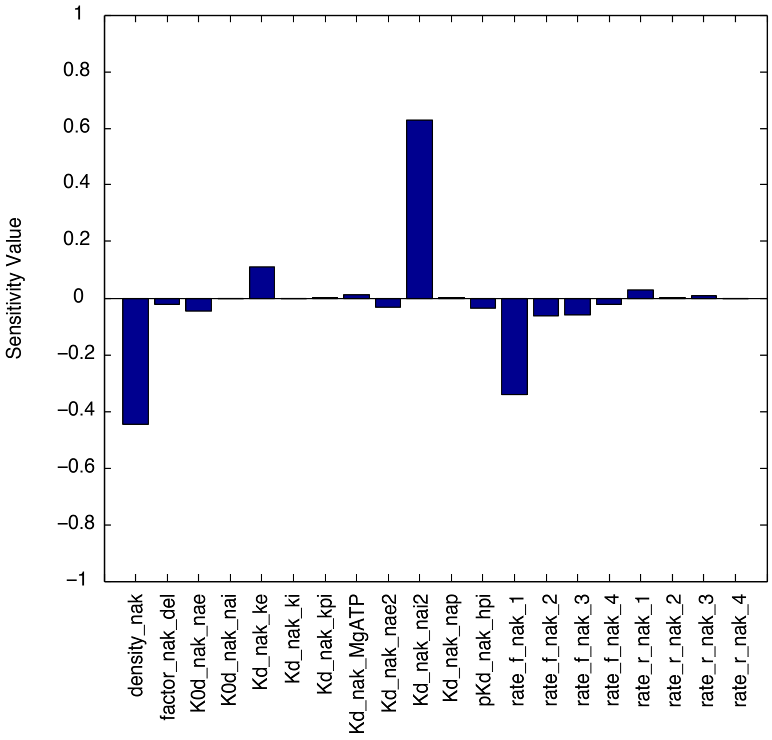
Sensitivity Value
1
0.8
0.6
0.4
0.2
0
−0.2
−0.4
−0.6
−0.8
−1
density_nak
factor_nak_del
K0d_nak_nae
K0d_nak_nai
Kd_nak_ke
Kd_nak_ki
Kd_nak_kpi
Kd_nak_MgATP
Kd_nak_nae2
Kd_nak_nai2
Kd_nak_nap
pKd_nak_hpi
rate_f_nak_1
rate_f_nak_2
rate_f_nak_3
rate_f_nak_4
rate_r_nak_1
rate_r_nak_2
rate_r_nak_3
rate_r_nak_4

Figure S3:

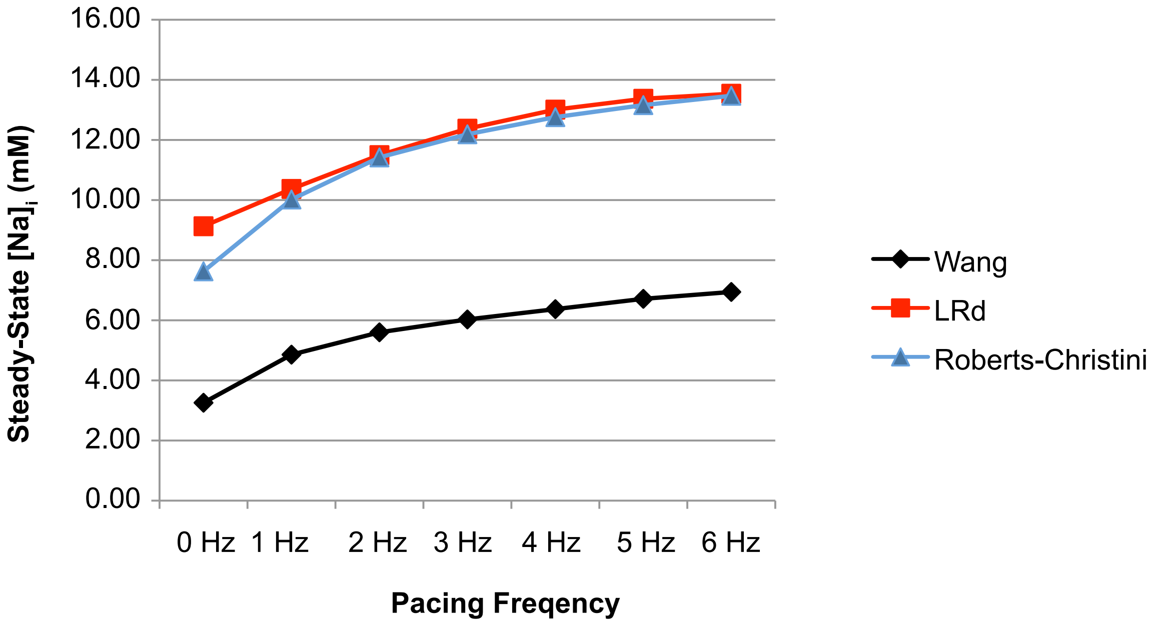
16.00
14.00
12.00
10.00
8.00
6.00
4.00
2.00
0.00
Steady-State [Na]i (mM)
0 Hz
1 Hz
2 Hz
3 Hz
4 Hz
5 Hz
6 Hz
Pacing Freqency
Wang
LRd
Roberts-Christini

Figure S4:

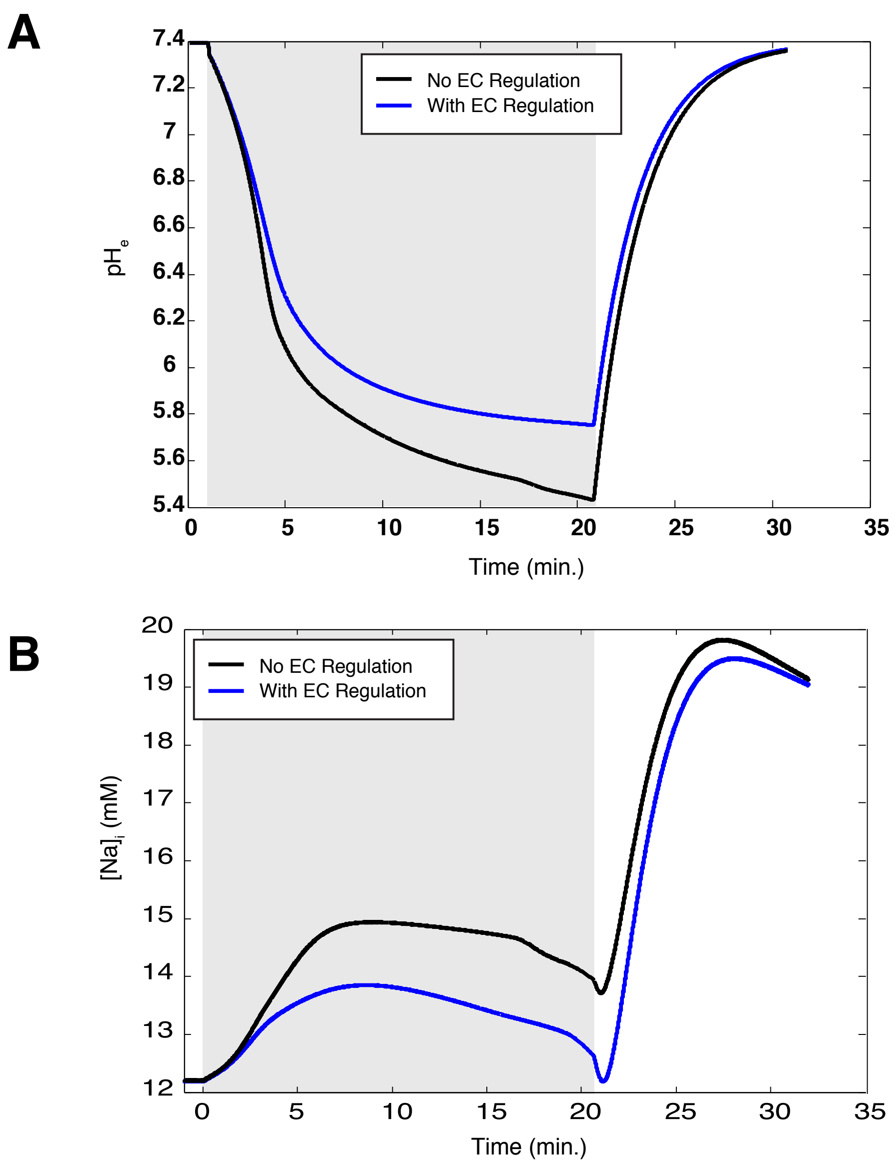
A
7.4
7.2
7
6.8
6.6
6.4
6.2
6
5.8
5.6
5.4
pHe
0
5
10
15
20
25
30
35
Time (min.)
No EC Regulation
With EC Regulation
B
20
19
18
17
16
15
14
13
12
[Na]i (mM)
0
5
10
15
20
25
30
35
Time (min.)
No EC Regulation
With EC Regulation

Figure S5:

References

1. Crampin EJ, Smith NP (2006) A dynamic model of excitation-contraction coupling during acidosis in cardiac ventricular myocytes. Biophys J 90: 3074-90.

2. Shigematsu S, Sato T, Abe T, Saikawa T, Sakata T, et al. (1995) Pharmacological evidence for the persistent activation of atp-sensitive k+ channels in early phase of reperfusion and its protective role against myocardial stunning. Circulation 92: 2266-75.

3. Terkildsen JR, Crampin EJ, Smith NP (2007) The balance between inactivation and activation of the na+-k+ pump underlies the triphasic accumulation of extracellular k+ during myocardial ischemia. Am J Physiol Heart Circ Physiol 293: H3036-45.

4. Gaur N, Rudy Y, Hool L (2009) Contributions of ion channel currents to ventricular action potential changes and induction of early afterdepolarizations during acute hypoxia. Circ Res 105: 1196-203.

5. Michailova A, Saucerman J, Belik ME, McCulloch AD (2005) Modeling regulation of cardiac katp and l-type ca2+ currents by atp, adp, and mg2+. Biophys J 88: 2234-49.

6. Tran K, Smith NP, Loiselle DS, Crampin EJ (2009) A thermodynamic model of the cardiac sarcoplasmic/endoplasmic ca(2+) (serca) pump. Biophys J 96: 2029-42.

7. Vaughan-Jones RD, Wu ML (1990) Extracellular h+ inactivation of na(+)-h+ exchange in the sheep cardiac purkinje fibre. J Physiol 428: 441-66.

8. Bountra C, Vaughan-Jones RD (1989) Effect of intracellular and extracellular ph on contraction in isolated, mammalian cardiac muscle. J Physiol 418: 163-87.

9. van Borren MMGJ, Baartscheer A, Wilders R, Ravesloot JH (2004) Nhe-1 and nbc during pseudo-ischemia/reperfusion in rabbit ventricular myocytes. J Mol Cell Cardiol 37: 567-77.

10. Leem CH, Lagadic-Gossmann D, Vaughan-Jones RD (1999) Characterization of intracellular ph regulation in the guinea-pig ventricular myocyte. J Physiol 517 (Pt 1): 159-80.

11. Sun B, Leem CH, Vaughan-Jones RD (1996) Novel chloride-dependent acid loader in the guinea-pig ventricular myocyte: part of a dual acid-loading mechanism. J Physiol 495 (Pt 1): 65-82.

12. Vaughan-Jones RD (1986) An investigation of chloride-bicarbonate exchange in the sheep cardiac purkinje fibre. J Physiol 379: 377-406.

13. Xu L, Mann G, Meissner G (1996) Regulation of cardiac ca2+ release channel (ryanodine receptor) by ca2+, h+, mg2+, and adenine nucleotides under normal and simulated ischemic conditions. Circ Res 79: 1100-9.

14. Doering AE, Lederer WJ (1994) The action of na+ as a cofactor in the inhibition by cytoplasmic protons of the cardiac na(+)-ca2+ exchanger in the guinea-pig. J Physiol 480 (Pt 1): 9-20.

15. Blanchard EM, Solaro RJ (1984) Inhibition of the activation and troponin calcium binding of dog cardiac myofibrils by acidic ph. Circ Res 55: 382-91.

16. Li G, Martin AF, Solaro JR (2001) Localization of regions of troponin i important in deactivation of cardiac myofilaments by acidic ph. J Mol Cell Cardiol 33: 1309-20.

17. Boron WF, De Weer P (1976) Intracellular ph transients in squid giant axons caused by co2, nh3, and metabolic inhibitors. J Gen Physiol 67: 91-112.

18. Leem CH, Vaughan-Jones RD (1998) Out-of-equilibrium ph transients in the guinea-pig ventricular myocyte. J Physiol 509 (Pt 2): 471-85.

19. Peluffo RD (2004) Effect of adp on na(+)-na(+) exchange reaction kinetics of na,k-atpase. Biophys J 87: 883-98.

20. Wuddel I, Apell HJ (1995) Electrogenicity of the sodium transport pathway in the na,k-atpase probed by charge-pulse experiments. Biophys J 69: 909-21.

21. Campos M, Beaugé L (1994) Na(+)-atpase activity of na(+),k(+)-atpase. reactivity of the e2 form during na(+)-atpase turnover. J Biol Chem 269: 18028-36.

22. Heyse S, Wuddel I, Apell HJ, Stürmer W (1994) Partial reactions of the na,k-atpase: determination of rate constants. J Gen Physiol 104: 197-240.

23. Peluffo RD, Berlin JR (2003) Na,k-pump reaction kinetics at the tip of a patch electrode: derivation of reaction kinetics for electrogenic and electrically silent reactions during ion transport by the na,k-atpase. Ann N Y Acad Sci 986: 141-9.

24. Hansen PS, Buhagiar KA, Kong BY, Clarke RJ, Gray DF, et al. (2002) Dependence of na+-k+ pump current-voltage relationship on intracellular na+, k+, and cs+ in rabbit cardiac myocytes. Am J Physiol Cell Physiol 283: C1511-21.

25. Nakao M, Gadsby DC (1989) [na] and [k] dependence of the na/k pump current-voltage relationship in guinea pig ventricular myocytes. The Journal of General Physiology 94: 539.

26. Friedrich T, Bamberg E, Nagel G (1996) Na+, k (+)-atpase pump currents in giant excised patches activated by an atp concentration jump. Biophysical journal 71: 2486-2500.

27. Mogul DJ, Singer DH, Ten Eick RE (1990) Dependence of na-k pump current on internal na+ in mammalian cardiac myocytes. Am J Physiol 259: H488-96.

28. Bahinski A, Nakao M, Gadsby DC (1988) Potassium translocation by the na+/k+ pump is voltage insensitive. Proc Natl Acad Sci U S A 85: 3412-6.

29. Lee SYY, Lee CO (2005) Inhibition of na+-k+ pump and l-type ca2+ channel by glibenclamide in guinea pig ventricular myocytes. J Pharmacol Exp Ther 312: 61-8.

30. Noma A, Shibasaki T (1985) Membrane current through adenosine-triphosphate-regulated potassium channels in guinea-pig ventricular cells. J Physiol 363: 463-80.

31. Nichols CG, Ripoll C, Lederer WJ (1991) Atp-sensitive potassium channel modulation of the guinea pig ventricular action potential and contraction. Circ Res 68: 280-7.

32. Clement JP, Kunjilwar K, Gonzalez G, Schwanstecher M, Panten U, et al. (1997) Association and stoichiometry of k(atp) channel subunits. Neuron 18: 827-38.

33. Shyng S, Nichols CG (1997) Octameric stoichiometry of the katp channel complex. J Gen Physiol 110: 655-64.

34. Ribalet B, John SA, Weiss JN (2003) Molecular basis for kir6.2 channel inhibition by adenine nucleotides. Biophys J 84: 266-76.

35. Hopkins WF, Fatherazi S, Peter-Riesch B, Corkey BE, Cook DL (1992) Two sites for adenine-nucleotide regulation of atp-sensitive potassium channels in mouse pancreatic beta-cells and hit cells. J Membr Biol 129: 287-95.

36. Gribble FM, Tucker SJ, Ashcroft FM (1997) The essential role of the walker a motifs of sur1 in k-atp channel activation by mg-adp and diazoxide. The EMBO Journal 16: 1145-1152.

37. Ueda K, Inagaki N, Seino S (1997) Mgadp antagonism to mg2+-independent atp binding of the sulfonylurea receptor sur1. J Biol Chem 272: 22983-6.

38. Steenbergen C, Hill ML, Jennings RB (1985) Volume regulation and plasma membrane injury in aerobic, anaerobic, and ischemic myocardium in vitro. effects of osmotic cell swelling on plasma membrane integrity. Circ Res 57: 864-75.

39. Tranum-Jensen J, Janse MJ, Fiolet WT, Krieger WJ, D'Alnoncourt CN, et al. (1981) Tissue osmolality, cell swelling, and reperfusion in acute regional myocardial ischemia in the isolated porcine heart. Circ Res 49: 364-81.

40. Befroy DE, Powell T, Radda GK, Clarke K (1999) Osmotic shock: modulation of contractile function, phi, and ischemic damage in perfused guinea pig heart. Am J Physiol 276: H1236-44.

41. Decking UK, Hartmann M, Rose H, Brückner R, Meil J, et al. (1998) Cardioprotective actions of kc 12291. i. inhibition of voltage-gated na+ channels in ischemia delays myocardial na+ overload. Naunyn Schmiedebergs Arch Pharmacol 358: 547-53.

42. Zweier JL, Jacobus WE (1987) Substrate-induced alterations of high energy phosphate metabolism and contractile function in the perfused heart. J Biol Chem 262: 8015-21.

43. Ji Y, Loukianov E, Loukianova T, Jones LR, Periasamy M (1999) Serca1a can functionally substitute for serca2a in the heart. Am J Physiol 276: H89-97.

44. Dode L, Vilsen B, Van Baelen K, Wuytack F, Clausen JD, et al. (2002) Dissection of the functional differences between sarco(endo)plasmic reticulum ca2+-atpase (serca) 1 and 3 isoforms by steady-state and transient kinetic analyses. J Biol Chem 277: 45579-91.

45. Sakamoto J, Tonomura Y (1980) Order of release of adp and pi from phosphoenzyme with bound adp of ca2+-dependent atpase from sarcoplasmic reticulum and of na+, k+-dependent atpase studied by adp-inhibition patterns. J Biochem 87: 1721-7.

46. Wang W, Ma J, Zhang P, Luo A (2007) Redox reaction modulates transient and persistent sodium current during hypoxia in guinea pig ventricular myocytes. Pflugers Arch 454: 461-75.

47. Sato R, Sakamoto K, Yamazaki J, Nagao T (2002) Differences in protective profiles of diltiazem isomers in ischemic and reperfused guinea pig hearts. Eur J Pharmacol 434: 125-31.

48. An J, Varadarajan SG, Camara A, Chen Q, Novalija E, et al. (2001) Blocking na(+)/h(+) exchange reduces [na(+)](i) and [ca(2+)](i) load after ischemia and improves function in intact hearts. Am J Physiol Heart Circ Physiol 281: H2398-409.

49. Fleet WF, Johnson TA, Graebner CA, Gettes LS (1985) Effect of serial brief ischemic episodes on extracellular k+, ph, and activation in the pig. Circulation 72: 922.

50. Kitakaze M, Takashima S, Funaya H, Minamino T, Node K, et al. (1997) Temporary acidosis during reperfusion limits myocardial infarct size in dogs. Am J Physiol 272: H2071-8.

51. Kleber AG (1983) Resting membrane potential, extracellular potassium activity, and intracellular sodium activity during acute global ischemia in isolated perfused guinea pig hearts. Circ Res 52: 442-450.

52. Faber GM, Rudy Y (2000) Action potential and contractility changes in [na+] i overloaded cardiac myocytes: a simulation study. Biophysical Journal 78: 2392-2404.
